# MASTREE+: Time‐series of plant reproductive effort from six continents

**DOI:** 10.1111/gcb.16130

**Published:** 2022-03-05

**Authors:** Andrew Hacket‐Pain, Jessie J. Foest, Ian S. Pearse, Jalene M. LaMontagne, Walter D. Koenig, Giorgio Vacchiano, Michał Bogdziewicz, Thomas Caignard, Paulina Celebias, Joep van Dormolen, Marcos Fernández‐Martínez, Jose V. Moris, Ciprian Palaghianu, Mario Pesendorfer, Akiko Satake, Eliane Schermer, Andrew J. Tanentzap, Peter A. Thomas, Davide Vecchio, Andreas P. Wion, Thomas Wohlgemuth, Tingting Xue, Katharine Abernethy, Marie‐Claire Aravena Acuña, Marcelo Daniel Barrera, Jessica H. Barton, Stan Boutin, Emma R. Bush, Sergio Donoso Calderón, Felipe S. Carevic, Carolina Volkmer de Castilho, Juan Manuel Cellini, Colin A. Chapman, Hazel Chapman, Francesco Chianucci, Patricia da Costa, Luc Croisé, Andrea Cutini, Ben Dantzer, R. Justin DeRose, Jean‐Thoussaint Dikangadissi, Edmond Dimoto, Fernanda Lopes da Fonseca, Leonardo Gallo, Georg Gratzer, David F. Greene, Martín A. Hadad, Alejandro Huertas Herrera, Kathryn J. Jeffery, Jill F. Johnstone, Urs Kalbitzer, Władysław Kantorowicz, Christie A. Klimas, Jonathan G. A. Lageard, Jeffrey Lane, Katharina Lapin, Mateusz Ledwoń, Abigail C. Leeper, Maria Vanessa Lencinas, Ana Cláudia Lira‐Guedes, Michael C. Lordon, Paula Marchelli, Shealyn Marino, Harald Schmidt Van Marle, Andrew G. McAdam, Ludovic R. W. Momont, Manuel Nicolas, Lúcia Helena de Oliveira Wadt, Parisa Panahi, Guillermo Martínez Pastur, Thomas Patterson, Pablo Luis Peri, Łukasz Piechnik, Mehdi Pourhashemi, Claudia Espinoza Quezada, Fidel A. Roig, Karen Peña Rojas, Yamina Micaela Rosas, Silvio Schueler, Barbara Seget, Rosina Soler, Michael A. Steele, Mónica Toro‐Manríquez, Caroline E. G. Tutin, Tharcisse Ukizintambara, Lee White, Biplang Yadok, John L. Willis, Anita Zolles, Magdalena Żywiec, Davide Ascoli

**Affiliations:** ^1^ 4591 Department of Geography and Planning School of Environmental Sciences University of Liverpool Liverpool UK; ^2^ U.S. Geological Survey Fort Collins Science Center Fort Collins Colorado USA; ^3^ 2453 Department of Biological Sciences DePaul University Chicago Illinois USA; ^4^ 1438 Hastings Reservation University of California Berkeley Carmel Valley California USA; ^5^ 9304 Department of Agricultural and Environmental Sciences University of Milan Milan Italy; ^6^ 49562 Faculty of Biology Institute of Environmental Biology Adam Mickiewicz University Poznań Poland; ^7^ INRAE LESSEM University Grenoble Alpes Grenoble France; ^8^ Univ. Bordeaux INRAE BIOGECO Pessac France; ^9^ Department of Computing University of London London UK; ^10^ 83020 CREAF Barcelona Catalonia Spain; ^11^ Department of Agricultural, Forest and Food Sciences (DISAFA) University of Torino Torino Italy; ^12^ Stefan cel Mare Univ Suceava Forestry Fac Appl Ecol Lab Suceava Romania; ^13^ 27270 Department of Forest and Soil Sciences Institute of Forest Ecology University of Natural Resources and Life Sciences Vienna Vienna Austria; ^14^ 12923 Kyushu University Fukuoka Japan; ^15^ Aix Marseille Univ Avignon Université CNRS IRD IMBE Marseille France; ^16^ 2152 Ecosystems and Global Change Group Department of Plant Sciences University of Cambridge Cambridge UK; ^17^ 4212 School of Life Sciences Keele University Staffordshire UK; ^18^ 3447 Graduate Degree Program in Ecology and The Department of Forest and Rangeland Stewardship Colorado State University Fort Collins Colorado USA; ^19^ Swiss Federal Institute for Forest, Snow and Landscape Research WSL Birmensdorf Switzerland; ^20^ College of Civil and Architecture and Engineering Chuzhou University China; ^21^ Faculty of Natural Sciences University of Stirling Stirling UK; ^22^ Institut de Recherche en Ecologie Tropicale CENAREST Libreville Gabon; ^23^ Facultad de Ciencias Forestales y de la Conservación de la Naturaleza (FCFCN) Universidad de Chile Santiago Chile; ^24^ Universidad Nacional de la Plata (UNLP) Buenos Aires Argentina; ^25^ Department of Biological Sciences University of Alberta Edmonton AB Canada; ^26^ Royal Botanic Garden Edinburgh Edinburgh UK; ^27^ Facultad de Recursos Naturales Renovables Universidad Arturo Prat Iquique Chile; ^28^ Brazilian Agricultural Research Corporation Embrapa Roraima Boa Vista Brazil; ^29^ Wilson Center Washington District of Columbia USA; ^30^ Department of Anthropology George Washington University Washington District of Columbia USA; ^31^ School of Life Sciences University of KwaZulu‐Natal Pietermaritzburg South Africa; ^32^ Shaanxi Key Laboratory for Animal Conservation Northwest University Xi'an China; ^33^ School of Biological Sciences University of Canterbury Canterbury New Zealand; ^34^ Nigerian Montane Forest Project (NMFP) Yelway Village Nigeria; ^35^ CREA—Research Centre for Forestry and Wood Arezzo Italy; ^36^ Brazilian Agricultural Research Corporation Embrapa Meio Ambiente Jaguariúna Brazil; ^37^ Département Recherche‐Développement‐Innovation Office National des Forêts Fontainebleau France; ^38^ Department of Psychology Department of Ecology and Evolutionary Biology University of Michigan Ann Arbor Michigan USA; ^39^ Department of Wildland Resources and Ecology Center Utah State University Logan Utah USA; ^40^ Agence Nationale des Parcs Nationaux (ANPN) Libreville Gabon; ^41^ Brazilian Agricultural Research Corporation Embrapa Acre Rio Branco AC Brazil; ^42^ Instituto de Investigaciones Forestales y Agropecuarias Bariloche (IFAB) (INTA—CONICET Instituto Nacional de Tecnología Agropecuaria—Consejo Nacional de Investigaciones Científicas y Técnicas Bariloche Argentina; ^43^ Department of Forestry and Wildland Resources Humboldt State University Arcata California USA; ^44^ Laboratorio de Dendrocronología de Zonas Áridas CIGEOBIO (CONICET‐UNSJ) Rivadavia Argentina; ^45^ Centro de Investigación en Ecosistemas de la Patagonia (CIEP) Coyhaique Chile; ^46^ Ulterarius Consultores Ambientales y Científicos Ltda Punta Arenas Chile; ^47^ Institute of Arctic Biology University of Alaska Fairbanks Fairbanks Alaska USA; ^48^ Department for the Ecology of Animal Societies Max Planck Institute of Animal Behavior Radolfzell Germany; ^49^ Department of Biology University of Konstanz Konstanz Germany; ^50^ Department of Silviculture and Genetics of Forest Trees Forest Research Institute Raszyn Poland; ^51^ 2453 Environmental Science and Studies Department DePaul University Chicago Illinois USA; ^52^ Department of Natural Sciences Manchester Metropolitan University Manchester UK; ^53^ Department of Biology University of Saskatchewan Saskatoon Saskatchewan Canada; ^54^ Austrian Research Centre for Forests BFW Vienna Austria; ^55^ Institute of Systematics and Evolution of Animals Polish Academy of Sciences Kraków Poland; ^56^ Centro Austral de Investigaciones Científicas (CADIC) Consejo Nacional de Investigaciones Científicas y Técnicas (CONICET) Ushuaia Argentina; ^57^ Brazilian Agricultural Research Corporation Embrapa Amapá Macapá Brazil; ^58^ Department of Biology and Institute of the Environment Wilkes University Wilkes‐Barre Pennsylvania USA; ^59^ Department of Ecology and Evolutionary Biology University of Colorado Boulder Colorado USA; ^60^ Independent researcher Saint‐Maur‐des‐Fossés France; ^61^ Brazilian Agricultural Research Corporation Embrapa Rondônia Porto Velho Brazil; ^62^ Botany Research Division Research Institute of Forests and Rangelands Agricultural Research, Education and Extension Organization Tehran Iran; ^63^ School of Biological, Environmental, and Earth Sciences The University of Southern Mississippi Hattiesburg Mississippi USA; ^64^ Instituto Nacional de Tecnología Agropecuaria (INTA) Universidad Nacional de la Patagonia Austral (UNPA) Consejo Nacional de Investigaciones Científicas y Técnicas (CONICET) Río Gallegos Argentina; ^65^ W. Szafer Institute of Botany Polish Academy of Sciences Kraków Poland; ^66^ Forest Research Division Research Institute of Forests and Rangelands Agricultural Research, Education and Extension Organization Tehran Iran; ^67^ Laboratorio de Dendrocronología e Historia Ambiental IANIGLA—CONICET‐Universidad Nacional de Cuyo Mendoza Argentina; ^68^ Facultad de Ciencias Hémera Centro de Observación de la Tierra Escuela de Ingeniería Forestal Universidad Mayor Santiago Chile; ^69^ Stony Brook University Long Island New York USA; ^70^ Ministère des Eaux, des Forêts, de la Mer, de l'Environnement chargé du Plan Climat, des Objectifs de Development Durable et du Plan d'Affectation des Terres Boulevard Triomphale Libreville Gabon; ^71^ Biosecurity NZ Ministry for Primary Industries Wellington New Zealand; ^72^ USDA Forest Service Auburn Alabama USA

**Keywords:** demography, flowering, general flowering, masting, plant reproduction, recruitment, regeneration

## Abstract

Significant gaps remain in understanding the response of plant reproduction to environmental change. This is partly because measuring reproduction in long‐lived plants requires direct observation over many years and such datasets have rarely been made publicly available. Here we introduce MASTREE+, a data set that collates reproductive time‐series data from across the globe and makes these data freely available to the community. MASTREE+ includes 73,828 georeferenced observations of annual reproduction (e.g. seed and fruit counts) in perennial plant populations worldwide. These observations consist of 5971 population‐level time‐series from 974 species in 66 countries. The mean and median time‐series length is 12.4 and 10 years respectively, and the data set includes 1122 series that extend over at least two decades (≥20 years of observations). For a subset of well‐studied species, MASTREE+ includes extensive replication of time‐series across geographical and climatic gradients. Here we describe the open‐access data set, available as a.csv file, and we introduce an associated web‐based app for data exploration. MASTREE+ will provide the basis for improved understanding of the response of long‐lived plant reproduction to environmental change. Additionally, MASTREE+ will enable investigation of the ecology and evolution of reproductive strategies in perennial plants, and the role of plant reproduction as a driver of ecosystem dynamics.

## Graphical abstract

MASTREE+ is an open‐access data set that collates reproductive time‐series data from across the globe. It includes 73,828 georeferenced observations of annual reproduction (e.g. seed and fruit counts) in perennial plant populations, consisting of 5,971 population‐level time‐series from 974 species. MASTREE+ will provide the basis for improved understanding of the response of plant reproduction to environmental change and can be used to investigate the ecology and evolution of plant reproductive strategies and the role of plant reproduction as a driver of ecosystem dynamics.

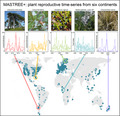

## Resumen

Aún existen importantes vacíos en la comprensión de la respuesta reproductiva de las plantas al cambio medioambiental, en parte, porque su monitoreo en especies de plantas longevas requiere una observación directa durante muchos años, y estos conjuntos de datos rara vez han estado disponibles. Aquí presentamos a MASTREE +, una base de datos que recopila series de tiempo de la reproducción de las plantas de todo el planeta, poniendo a disposición estos datos de libre acceso para la comunidad científica. MASTREE + incluye 73.828 puntos de observación de la reproducción anual georreferenciados (ej. conteos de semillas y frutos) en poblaciones de plantas perennes en todo el mundo. Estas observaciones consisten en 5971 series temporales a nivel de población provenientes de 974 especies en 66 países. La mediana de la duración de las series de tiempo es de 10 años (media = 12.4 años) y el conjunto de datos incluye 1.122 series de al menos dos décadas (≥20 años de observaciones). Para un subconjunto de especies bien estudiadas, MASTREE +incluye un amplio conjunto de series temporales replicadas en gradientes geográficos y climáticos. Describimos el conjunto de datos de acceso abierto disponible como un archivo.csv y presentamos una aplicación web asociada para la exploración de datos. MASTREE+ proporcionará la base para mejorar la comprensión sobre la respuesta reproductiva de plantas longevas al cambio medioambiental. Además, MASTREE+ facilitará los avances en la investigación de la ecología y la evolución de las estrategias reproductivas en plantas perennes y el papel de la reproducción vegetal como determinante de la dinámica de ecosistemas.

## 1 INTRODUCTION

Climate change and other anthropogenic drivers are altering plant demographics, with reported changes in plant mortality, growth and reproduction (Allen et al., 2010; McDowell et al., 2020; Pearse et al., 2017; Senf et al., 2018). These demographic shifts are changing the composition and structure of vegetation, with far‐reaching effects on ecosystem functioning and services, including complex effects on biodiversity and terrestrial carbon sinks (Carnicer et al., 2011; Chen et al., 2019; Clark et al., 2016; Ruiz‐Benito et al., 2017). In most plant species, seed production is a key process limiting sexual reproduction. However, our understanding of climate‐driven changes in seed production lags behind other key demographic processes such as growth and mortality (Clark et al., 2021), where inventory data, tree‐ring networks and remote sensing have transformed understanding of responses to environmental change (Buras et al., 2020; Changenet et al., 2021; Klesse et al., 2020). Reproduction and other processes associated with plant recruitment require direct and intensive field‐based observation over many years. However, there have been few previous attempts to collate, archive and make available original data from long‐term monitoring studies across taxa and wide geographic areas (Ascoli, Maringer, et al., 2017; Koenig & Knops, 2000; Pearse et al., 2020). Consequently, the response of plant reproduction to ongoing environmental change remains poorly understood, and paucity of data compromises the parameterisation of reproduction in models used to predict future vegetation dynamics (Fisher et al., 2018; Vacchiano et al., 2018).

Recent analysis of long‐term data sets indicates that seed production may be sensitive to climate change. Where increases in temperature favour reproduction, warming is linked to increased seed production (Bogdziewicz et al., 2020; Buechling et al., 2016; Caignard et al., 2017), whereas in drought‐limited populations seed production has declined in association with warming (Redmond et al., 2012). Additionally, environmental change may alter the interannual variability and spatial synchrony of reproduction (Hacket‐Pain & Bogdziewicz, 2021; Pearse et al., 2017). These shifts in reproduction have consequences for recruitment and wider ecosystem dynamics (Pau et al., 2018; Redmond et al., 2012; Schupp et al., 2019). For example, long‐term reductions in tropical rainforest fruit production have been linked with declining vitality of herbivorous megafauna (Bush et al., 2020), and low seed availability can limit forest recovery after large‐scale mortality events (Redmond et al., 2018). Beyond changes in mean seed and fruit production, shifts in the spatiotemporal variability of flowering and fruiting (i.e. masting) will also have impacts on key ecosystem services and habitat management (Pearse et al., 2021) including commercial and subsistence food crops (Calama et al., 2011; Ladio & Lozada, 2004; Shelef et al., 2017), seed‐eating animal population dynamics (Touzot et al., 2020), and human health through the trophic interactions that drive vector‐borne zoonotic disease dynamics (Bennett et al., 2010; Bregnard et al., 2020). However, the direction and magnitude of reported changes in masting are inconsistent, and this variability in response remains poorly understood (Hacket‐Pain & Bogdziewicz, 2021).

**FIGURE 1:**
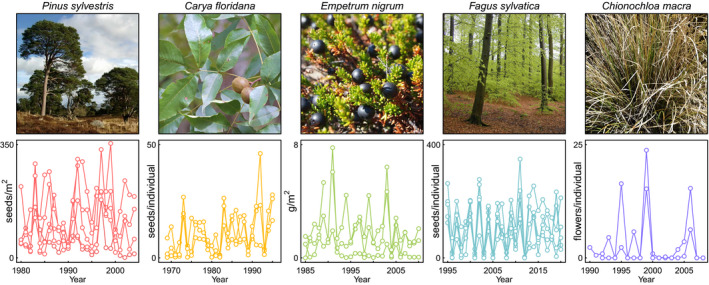
Examples of population‐level time‐series of reproductive effort from MASTREE+. For five diverse plant species, data from several local populations are plotted to illustrate the range of spatiotemporal variation in reproduction that is typical in long‐lived plants. Note that axis scales and units vary between plots

As the magnitude of plant reproduction is highly variable across time and space (Figure 1), multi‐decadal time‐series of plant reproductive effort with high replication and sampling across environmental gradients are needed to derive meaningful inferences and predictions from modelling efforts (Clark et al., 2021; Pearse et al., 2021; Pennekamp et al., 2019; Vacchiano et al., 2018). The availability of such data will enable robust estimates of the response of plant reproduction to recent environmental change, and through identification of the underlying drivers, prediction of future trends. MASTREE+ provides these time‐series of plant reproductive effort, and will enable testing of changes in masting patterns associated with recent environmental change across multiple species and geographical regions (Hacket‐Pain & Bogdziewicz, 2021; LaMontagne et al., 2021; Pearse et al., 2017). Such data sets will also enable new insights into the ecology and evolution of perennial plant reproduction (Dale et al., 2021), and the role of plant reproduction as a driver of other ecological processes including plant recruitment and animal population dynamics (Brumme et al., 2021; Connell & Green, 2000; Curran & Leighton, 2000; Schupp et al., 2019).

## 2 MASTREE+

Here we introduce a project to collate data of perennial plant reproductive time‐series. Time‐series originate from diverse sources, including 17th century European forestry records of seed production (‘mast years’) (Ascoli, Vacchiano, et al., 2017), data from ongoing plant reproductive biology and phenology monitoring programmes (e.g. RENECOFOR, LTER, California Acorn Survey), and projects studying the dynamics of ecosystems including the relationships between seed production and animal demographics (Boutin et al., 2006). Many of these data sets record the number or mass of flowers, seeds, fruits or cones per individual or unit area on a continuous scale. We also include ordinal time‐series, which record annual reproduction output according to an ordered categorical scale (e.g. failure/partial/full crop) which can be successfully used to investigate the variability and synchrony of plant reproduction (Bogdziewicz et al., 2021).

The current version of MASTREE+ currently includes 5971 species‐specific and georeferenced time‐series representing 73,828 annual observations of reproductive effort in perennial plant populations, and the project is designed to continue to assemble and update records (see Sections 4 and 5). Mean and median time‐series length are 12.6 and 10 years respectively. 2846 series are based on continuous measures of reproductive effort, and 3125 are ordinal series. Ordinal series originate mainly from Europe. Importantly, MASTREE+ contains 1122 time‐series ⋝20 years, of which 187 time‐series exceed 40 years of observations. Such records will enable quantification of recent changes in plant reproduction, including mean reproductive effort and spatiotemporal variability, and the identification of key drivers of change.

**FIGURE 2:**
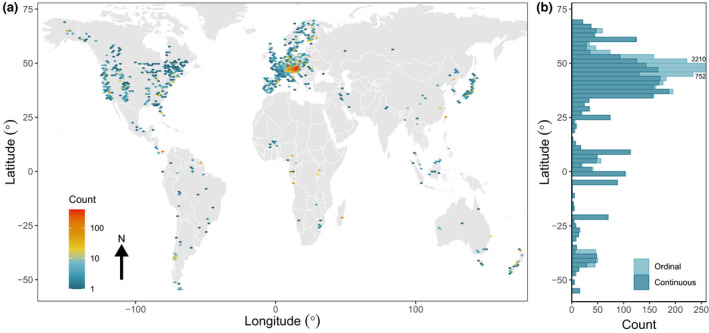
The geographical distribution of time‐series within MASTREE+. The (a) spatial and (b) latitudinal distribution of species‐specific time‐series. For (b), series are stacked and coloured according to the variable type (Continuous, Ordinal). Plotting of counts for ordinal data in the northern mid‐latitudes are truncated due to high sampling intensity in central Europe. Unprojected map, datum = WGS84

**FIGURE 3:**
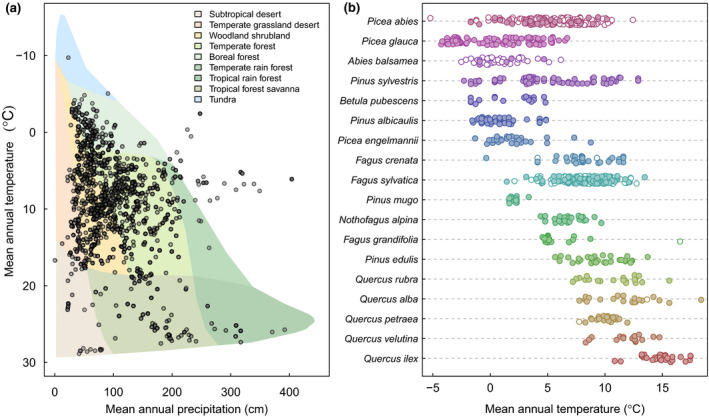
Distribution of time‐series in MASTREE+ according to local climate (Worldclim v2.1, 30 arcsecond resolution, Mosier et al., 2014). Only time‐series representing reproduction at the stand or patch scale are plotted (regional records are excluded, as local climate data based on coordinates may not be representative). (a) Series plotted according to Whittaker biomes (Whittaker, 1970) and (b) Species with high replication (≥20 species‐specific time‐series), plotted according to local mean annual temperature. Species are labelled according to the first three characters of the genus followed by the first three characters of the species name, and species are ordered according to the sample site with the lowest mean annual temperature. Unfilled points represent ordinal time‐series and filled points represent continuous time‐series

In total, 974 species are represented, drawn from 136 families across the plant Tree of Life. This increases species representation by 168% compared with the largest previously available compilation (Pearse et al., 2020), which is incorporated into MASTREE+. This expands the potential to quantify reproductive traits that describe the spatiotemporal variability of reproduction (i.e. masting) with other life‐history traits to better understand the evolution of plant reproductive strategies (Dale et al., 2021; Fernandez‐Martinez et al., 2019; Pesendorfer et al., 2021). For example, we have 67 species overlap with the plant demographic database COMPADRE (Salguero‐Gomez et al., 2015), 442 species overlap with seed mass data from the Kew Seed Information Database (Royal Botanic Gardens Kew, 2021) and 82 species overlap with the seed germination database SylvanSeeds (Fernandez‐Pascual, 2021). Reflecting a bias in sampling to temperate forests, woody species from the genera *Quercus* (60 species), *Nothofagus* (10), *Pinus* (25), *Abies* (13), *Acer* (13) and *Eucalyptus* (15) are highly represented, but other well‐represented genera include *Acacia* (11), *Shorea* (9) and *Chionochloa* (11). We include data from 66 countries, six continents (Figure 2), and from all the major vegetated biomes (Figure 3). Importantly, we increase data representation from regions that have been unrepresented in previous data sets (Ascoli, Maringer, et al., 2017; Pearse et al., 2020), including south and central America, Africa, and Asia, although these regions remain strongly under‐represented.

Sampling intensity varies between species. For example, 71% of species are represented by a single time‐series, but other species have high replication, often covering large parts of their geographical distribution. 51 species are represented by at least 10 location‐specific time‐series. The most replicated species are *Fagus sylvatica* (913 site‐specific time‐series), *Picea abies* (844), *Pinus sylvestris* (419), *Larix decidua* (395), *Abies alba* (393), *Quercus robur* (188), *Quercus petraea* (161), *Pinus cembra* (135) and *Picea glauca* (108). These and other well‐replicated species include data spanning large climatic gradients (Figure 3). These records will enable investigation of intraspecific variation in plant reproduction across climate, space, and time, including trends in the spatiotemporal variability of reproduction. It will also enable comprehensive assessments of intraspecific variability of masting characteristics (i.e. interannual variability, autocorrelation), including variation with environmental conditions that are predicted by theory but have rarely been tested (Pearse et al., 2020; Pesendorfer et al., 2021), and analysis of interspecific variation in spatial synchrony of reproduction (Dale et al., 2021), in functionally diverse plant species.

## 3 APPLICATIONS OF MASTREE+

**FIGURE 4:**
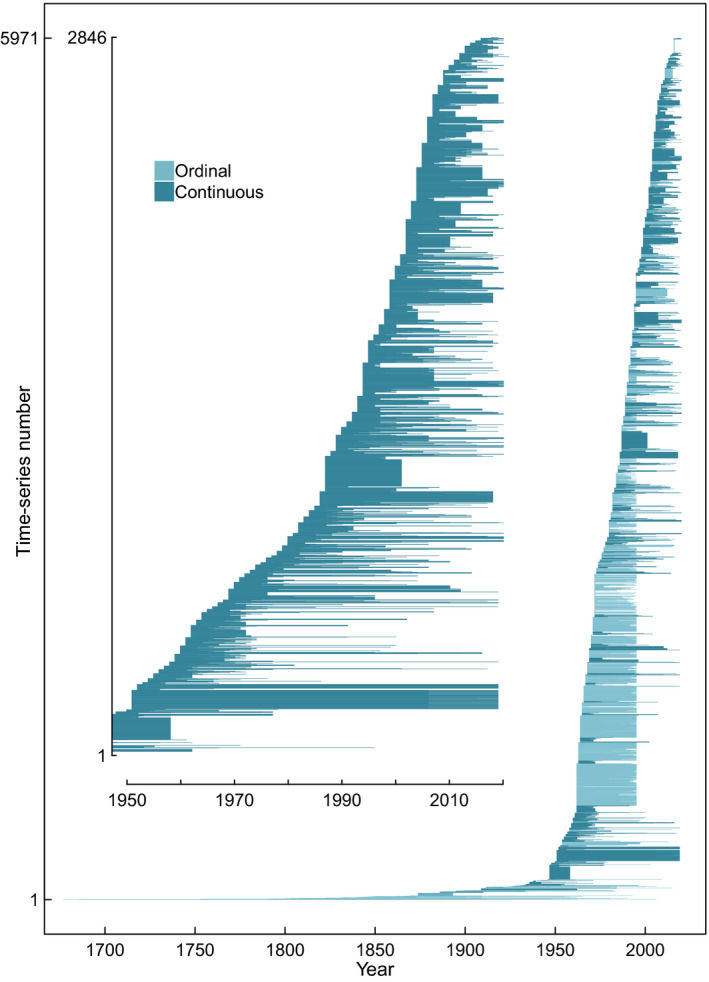
Timespans covered by species‐specific time‐series in MASTREE+, coloured by data class. Inset plot shows continuous data since 1950 when time‐series replication is highest

MASTREE+ provides the data sets to establish how fecundity, and specifically seed masting, responds to environmental change. It includes the high replication of long time‐series required to isolate climate change effects on plant reproductive effort (Hacket‐Pain & Bogdziewicz, 2021; Mundo et al., 2021) (Figure 4), while high spatial replication across environmental gradients (e.g. Figure 3b) provides the opportunity for a complementary space‐for‐time substitution approach (Wion et al., 2020). The expected response of masting to climate change remains unresolved, and MASTREE+ will enable testing of contrasting predictions that masting will be unresponsive to trends in mean temperature (Kelly et al., 2013), or will shift predictably based on climate‐driven changes in resource limitation (Bogdziewicz, 2021). Resolving this uncertainty is a priority because changes in seed masting will impact plant reproductive success, and more widely affect ecosystem services and habitat management (Ida, 2021; Pearse et al., 2021; Touzot et al., 2020).

In systems where seed production limits recruitment, MASTREE+ can be utilised to understand the drivers of plant reproduction and regeneration (Abraham et al., 2018; Manríquez et al., 2016; Oliva et al., 2013). Intraspecific differences in fecundity and masting influence regeneration success, determining species composition and vegetation structure, including during the colonisation of new habitats (Joubert et al., 2013), and after natural and anthropogenic disturbance (Martin‐DeMoor et al., 2010; Mokake et al., 2018; Peters et al., 2005). Masting characteristics of hundreds of species can be investigated using MASTREE+, and integration with plant trait and demographic databases will enable deeper integration of masting and reproductive strategies within life history theory (Salguero‐Gomez et al., 2016). Many ecologically and economically important species show highly variable investment in reproduction between years, and the ability to accurately forecast occasional years of high seed production is a priority for habitat management, with wide ranging applications (Chiavetta & Marzini, 2021; Pearse et al., 2021; Pukkala et al., 2010). Predictive models of masting developed and tested using MASTREE+ data may enable more effective seed collection for afforestation and restoration schemes (Fargione et al., 2021; Kettle et al., 2010), inform wildlife and conservation management (Choquenot & Ruscoe, 2000; Fujiki, 2018; Ida, 2021; O'Donnell & Hoare, 2012), and enable forecasting of periods of elevated infection risk from tick‐borne disease, which predictably follow years of high seed production in many forest ecosystems (Brugger et al., 2018; Cunze et al., 2018; Heyman et al., 2012; Ostfeld et al., 1996).

The availability of seed and fruit production data sets in MASTREE+ will be broadly relevant when paired with existing animal population data sets. The pulses of resources associated with large reproductive events are key drivers of the population dynamics of seed‐eating insects, mammals and birds, with cascading impacts through ecosystems (Bouchard et al., 2018; Kanamori et al., 2017; Selonen et al., 2016). Time‐series in MASTREE+ can be combined with existing long time‐series of animal populations and behaviour to identify the drivers of population dynamics, both in seed‐dependent species and further down the trophic cascade (Kleef & Wijsman, 2015; Lithner & Jönsson, 2002). Where species are well replicated in MASTREE+, the spatial synchrony of masting can also be quantified, allowing researchers to determine where regional estimates of masting can be appropriately used as indicators of local variability in seed or fruit availability. The scale of spatial synchrony of masting appears to be highly variable between species (Bogdziewicz et al., 2019), but this has only been quantified of a handful of species so far (Koenig & Knops, 2013; LaMontagne et al., 2020).

In masting species, highly variable allocation to reproduction has wider effects on plant resource allocation, and carbon and nutrient cycling through ecosystems, but this remains poorly explored (Brumme et al., 2021; Khanna et al., 2009; Muller‐Haubold et al., 2015). Data in MASTREE+ can be combined with existing field and remote‐sensing data sets of plant growth or productivity, and with data sets of whole‐ecosystem or soil carbon and nutrient fluxes to understand how variable allocation to reproduction influences carbon sequestration above and belowground, and how this varies between species and across environmental gradients (Bajocco et al., 2021; Nussbaumer et al., 2021; Oddou‐Muratorio et al., 2021; Zhang et al., 2022). Related work can use MASTREE+ data combined with existing or retrospective sampling (e.g. tree‐rings) to address outstanding question regarding resource allocation between growth, reproduction, and defence, particularly how this varies interspecifically and with environmental stress, and how this may shape species and community responses to environmental change (Lauder et al., 2019; Redmond et al., 2019).

## 4 DATA SOURCES, ACQUISITION AND COMPILATION

We collected species‐specific time‐series of annual reproductive effort for terrestrial perennial plant populations, including trees, shrubs, herbs and grasses. We included data from unmanaged and managed populations, but excluded agricultural crop species subject to selective breeding. Where reproduction was monitored under experimentally manipulated conditions (e.g. fertilisation, warming, rainfall exclusion), we only included data from control plots.

Data were collected for reproductive effort at different stages of the reproductive cycle (e.g. flowers or inflorescences, pollen abundance, number of fruits, cones or seeds), but 90% of data were mature seed, fruit, or cone production. We did not set a minimum time‐series length but prioritised compiling effort on time‐series ≥4 years. All time‐series represent reproductive effort at the population level, ranging from local populations with <10 individuals to regional estimates of reproduction, and we recorded information on the number of monitored individuals in each population and the spatial scale represented by the time‐series (Table 1). We also included information on the original data collection methods, which included litter traps (19.3% of all records), seed, cone and fruit counts (18.3%), other methods including estimates of cone production using cone or fruit scars and categorical classification of seed and fruit crops by wildlife managers or foresters.

**TABLE 1:** Overview of the data variables in the MASTREE+ data set. A more detailed description of the variables is included in Appendix 5

Variable	Description
Alpha_Number	Unique code associated with each original source of data, that is, the publication, report or thesis containing extracted data, or the previously unpublished data set included in MASTREE+
Segment	Temporal segment of a time‐series containing gaps (note that years with no observations are not recorded). Individual time‐series can consist of multiple segments
Site_number	Code to differentiate multiple sites from the same original source (Alpha_Number/Study_ID)
Variable_number	Code to differentiate multiple measures of reproductive output from the same species‐site combination (e.g. where seeds and cones were recorded separately)
Year	Year of observation
Species	Species identifier, standardised to The Plant List nomenclature. ‘spp.’ is used to indicate a record identified to the genus level only. ‘MIXED’ indicates a non‐species‐specific community‐level estimate of annual reproductive effort
Species_code	Six‐character species identifier
Mono_Poly	Monocarpic (semelparous) or Polycarpic (iteroparous) species
Value	The measured value of annual reproductive output
VarType	Continuous or ordinal data. Continuous time‐series are recorded on a continuous scale. Ordinal series are recorded on an ordered categorical scale. All ordinal series are rescaled to start at 1 (lowest reproductive effort) and to contain only integer values
Unit	The unit of measurement, where VarType is continuous
Max_Value	The maximum value in a time‐series
Variable	Categorical classification of the measured variable. Options limited to: cone, flower, fruit, seed, pollen, total reproduction organs
Collection_method	Classification of the method used to measure reproductive effort. Options are limited to: cone count, cone scar count, flower count, fruit count, fruit scar sound, seed count, seed trap, pollen count, lake sediment pollen count, harvest record, visual crop assessment, other quantification, dendrochronological reconstruction
Latitude	Latitude of the record, in decimal degrees
Longitude	Longitude of the record, in decimal degrees
Coordinate_flag	A flag to indicate the precision of the latitude and longitude. A = coordinates provided in the original source B = coordinates estimated by the compiler based on a map or other location information provided in the original source C = coordinates estimated by the compiler as the approximate centre point of the smallest clearly defined geographical unit provided in the original source (e.g. county, state, island), and potentially of low precision
Site	A site name or description, based on information in the original source
Country	The country where the observation was recorded
Elevation	The elevation of the sample site in metres above sea level, where provided in the original source
Spatial_unit	Categorical classification of spatial scale represented by the record, estimated by the compiler based on information provided in the original source. stand = <100 ha patch = 100–10,000 ha region = 10,000–1,000,000 ha super‐region = >1,000,000 ha
No_individuals	Either the number of monitored individual plants, or the number of litter traps. NA indicates no information in the original source, and 9999 indicates that while the number of monitored individuals was not specified, the source indicated to the compiler that the sample size was likely ≥10 individuals or litter traps
Start	The first year of observations for the complete time‐series, including all segments
End	The final year of observations for the complete time‐series, including all segments
Length	The number of years of observations. Note that may not be equal to the number of years between the Start and End of the time‐series, due to gaps in the time‐series.
Reference	Identification for the original source of the data, see Appendix 4 for the complete list of references
Record_type	Categorisation of the original source. Peer‐reviewed = extracted from peer reviewed literature Grey = extracted from grey literature Unpublished = unpublished data
ID_enterer	Identification of the original compiler of the data. AHP, Andrew Hacket‐Pain; ES, Eliane Schermer; JVM, Jose Moris; XTT, Tingting Xue; TC, Thomas Caignard; DV, Davide Vecchio; DA, Davide Ascoli; IP, Ian Pearse; JL, Jalene LaMontagne; JVD, Joep van Dormolen
Date_entry	Date of data entry into MASTREE+ in the format yyyy‐mm‐dd
Note on data location	Notes on the location of the data within the original source, such as page or figure number
Comments	Additional comments
Study_ID	Unique code associated with each source of data. M_ = series extracted from published literature; A_ = series incorporated from Ascoli et al. (2020), Ascoli, Maringer, et al. (2017) and Ascoli, Vacchiano, et al. (2017); PLK_ = series incorporated from Pearse et al. (2017); D_ = unpublished data sets

Data were collected from several sources. We harmonised data from previously published compilations of plant reproductive effort displaying differences in data architecture (Ascoli et al., 2020; Ascoli, Maringer, et al., 2017; Pearse et al., 2020). To identify other time‐series, we searched Google Scholar and Scopus with multiple combinations of search terms (see Appendix 2). Spanish‐ and French‐language searches was used to increase data representation from South America and Africa. An initial screen was based on the title and abstract to exclude irrelevant sources. Then, potential sources were classified based on the inclusion of useful time‐series data of reproductive effort, available as either data tables, figures, descriptions in the text or in supplementary data files or in online data repositories. Finally, we solicited contributions of previously unpublished data sets from our research networks. Time‐series were extracted from the original sources. In the case of values published in tables, in the text, or in online data repositories or supplementary data files, we extracted values directly from the source. In cases where data were contained in figures, we used the WebPlotDigitizer tool (Rohatgi, 2020). Metadata associated with each time‐series was also extracted from the sources, or directly from data set contributors, and copies of original sources were archived.

### 4.1 Data set variables

For each monitored population we recorded annual observations of reproductive effort, the units of measurement, the method used to assess reproductive output and the number of monitored individuals (Table 1). Where multiple measures of reproductive output were recorded for the same population (e.g. where seeds and cones were recorded separately), this was recorded to enable filtering of the data set for pseudoreplicates (Table 1). For ordinal series, we maintained the original number of classes, but we rescaled to integer scales starting at 1 (lowest reproductive output). For continuous series, where possible we converted data into a common unit (e.g. we converted ‘seeds/ha’ to ‘seeds/m^2^’). Years with missing observations are not recorded, and time‐series that would otherwise have gaps consist of a set of segments. The *Start* and *End* year corresponds to the first and last observation year for each time‐series, respectively, including all segments. *Length* is the number of observations within each time‐series, and can therefore be lower than the number of years between the *Start* and *End*. The location (decimal degrees), site name, elevation and country of each time‐series were recorded. The spatial scale represented by the time‐series was estimated on a four‐point scale, from individual stand to region, based on information contained in the original source. Information on the nature of the source, and reference information was also recorded. Full details of data variables are listed in Table 1. Each time‐series can be uniquely defined by combining *Alpha_Number*, *Site_number*, *Variable_number* and *Species_code*.

### 4.2 Technical validation and quality control

A two‐stage approach was adopted to validate time‐series data. Initially, we standardised attribute data and checked for errors and inconsistencies within time‐series. Species names were checked and standardised to The Plant List nomenclature, using the ‘Taxonstand’ package for R (v. 2.3) (Cayuela et al., 2021). Country names were converted to the English short name (ISO3166‐1) using the ‘countrycode’ package for R (v. 1.2.0) (Arel‐Bundock et al., 2018). Automatic checks were performed to ensure that each time‐series was uniquely identified by the identification variables and that time‐series' observations were uniquely identified by *Year*. *Species_code* was assigned by automatically combining the first three characters from the TPL‐standardised genus and species names. Where separate species shared a *Species_code*, a unique combination was manually created. The final character of *Species_code* for populations of a hybrid origin was changed to ‘X’. We ran various automatic checks to ensure all observations in a time‐series had uniform attribute data where such uniformity was expected (i.e. within a time‐series, there was only a single value for variables such as *Unit*). Interrelated variables were checked to ensure consistency, for example that time‐series spatial data (*Latitude*, *Longitude*) fell within the boundaries of the indicated *Country*. Time‐series duration variables (i.e. *Segment*, *Start*, *End*, *Length*) were directly calculated from time‐series.

The second stage involved the detection and removal of duplication problems between time‐series, that is, series added multiple times, including with partial overlap, usually when data were published in more than one source. First, we created ‘potential duplication groups’ that contained sets of time‐series that shared the same study species and approximate location (using a ±0.1 decimal degree buffer between pairs of time‐series). PDGs containing time‐series from multiple sources (*Alpha_Number*) were then inspected further. Suspect pairs of time‐series within PDGs were initially identified based on a correlation test (Spearman's *ρ* > 0.97), and we then inspected manually for duplication using information including location, units, and collection methods to identify possible duplication. To supplement the semi‐automated detection of duplicates, we performed a further manual check, examining groups of time‐series that shared the same country and species. Suspect pairs of series might, for example, share matching spatial references, matching site descriptions and/or matching author names.

Where duplicated series were identified, or where independence could not be confirmed, we selected a single time‐series for inclusion in MASTREE+. Generally, the longest time‐series was prioritised, unless there were clear signs that a shorter time‐series was of higher quality (e.g. the data were directly shared by the author and not extracted from a graph).

## 5 DATA SET AVAILABILITY AND MASTREE+ DATA EXPLORER

**FIGURE 5:**
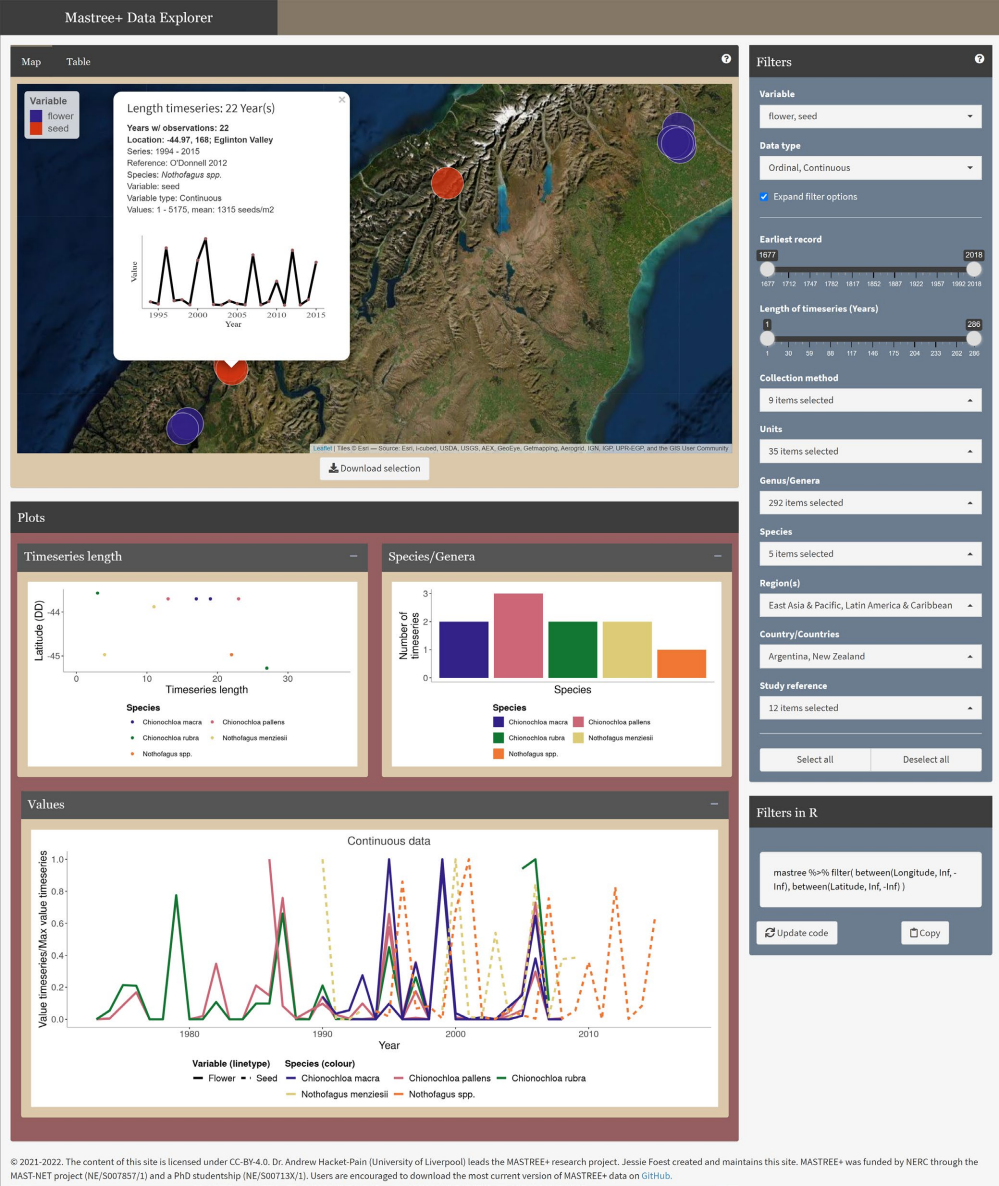
Example of the MASTREE+ Shiny Data Explorer, showing data from the South Island of New Zealand. The Data Explorer allows the user to explore data availability within MASTREE+, and download the full or user‐defined subsets of the data set

The data set is provided as a csv file in the online supporting information (Appendix 1) and is distributed under a CC‐BY‐4.0 licence so that it can be freely used, shared and modified so long as appropriate credit is given. The data set will be expanded and updated over time, so users are encouraged to check for the latest version of the data set on GitHub (https://github.com/JJFoest/MASTREEplus) and via associated updates to the MASTREE+ Data Explorer. The MASTREE+ Data Explorer allows users to explore the MASTREE+ data set and provides an alternative for downloading the data set, including user‐defined subsets thereof. The MASTREE+ Data Explorer was created using the *shiny* package in R (Chang et al., 2021) and can be accessed at https://mastreeplus.shinyapps.io/mastreeplus/. Time‐series are plotted on a zoomable world map, with updating summary plots showing the time‐series lengths and species/genera for the selected region, as well as scaled time‐series for initial visualisation of the data within the selected region of interest (Figure 5). Individual time‐series can be selected on the map to reveal associated meta‐data, including the location, species and original source. Various filter options allow the user to subset the full data set. An R script is provided in Appendix 6 that illustrates how to load, manipulate and visualise the data set.

## 6 CALL FOR DATA

We have increased taxonomic and geographic representation in MASTREE+, but many gaps remain in the coverage of our data set. Our goal is to provide a global platform for sharing data on long‐lived plant reproduction, and we encourage scientists to submit time‐series of annual reproductive effort in perennial plant populations for inclusion in MASTREE+ (Table 2). We will consider all species‐specific time‐series of four or more years, including continuous and ordinal observations. We include time‐series data on flower, seed, fruit and cone production, which are associated with geographical coordinates. We can include data that represent small local populations through to large regional‐scale assessments of reproductive effort. Note that we only record annual reproductive effort. Where data are collected at sub‐annual timesteps, this means that reproduction must be aggregated to annual units (e.g. April–March).

**TABLE 2:** Minimum data requirements for submissions to MASTREE+. For further details see Table 1

Minimum data requirements and metadata
Minimum of four consecutive measurements of annual reproductive output
Measurement at the population level (local population through regional scale estimates acceptable)
Species name according to The Plant List. Records identified to the genus level are acceptable, and measurements of non‐species‐specific community reproductive effort may be included.
Spatial coordinates of the monitored population
Details of the method used to measure reproductive effort (e.g. litter traps, seed counts, visual crop estimate, see Table 1)

Potential contributors of data are encouraged to search the latest version of the data set to check whether the data are already included in MASTREE+, either by downloading the latest version from GitHub, Dryad (Section 5) or via the MASTREE+ Data Explorer. If the data are not already included, potential contributors are encouraged to contact the corresponding author to discuss arrangements for sharing data. The minimum data requirements are included in Table 2.

### 6.1 Data licence

MASTREE+ is published under a CC‐BY‐4.0 licence, which enables users to copy and redistribute, adapt and modify the data set in any medium or format and for any purpose, including commercial. You must give appropriate credit by citing this publication, provide a link to the license and indicate if changes were made (see https://creativecommons.org/licenses/by/4.0/ for further details). Data can be accessed via Github: https://github.com/JJFoest/MASTREEplus, Dryad: https://doi.org/10.5061/dryad.18931zd02, or via the MASTREE+ Siny App. Publications using the RENECOFOR data (Reference = RENECOFOR_2020) are requested to acknowledge the RENECOFOR network, and send copies of publications to manuel.nicolas@onf.fr. Publications using the Lopé data (Reference = Bush_2021) are requested to cite the original data set (http://hdl.handle.net/11667/152), acknowledge The National Parks Agency of Gabon (ANPN) and the University of Stirling, and send copies of any resulting publications to science@parcsgabon.ga and k.a.abernethy@stir.ac.uk.

## CONFLICT OF INTEREST

The authors declare no conflict of interest.

## AUTHOR CONTRIBUTIONS

*Conceptualisation*: Andrew Hacket‐Pain, Ian S. Pearse, Walter D. Koenig, Giorgio Vacchiano, Michał Bogdziewicz, Mario Pesendorfer, Akiko Satake, Andrew J. Tanentzap, Peter A. Thomas, Thomas Wohlgemuth, Davide Ascoli. *Methodology*, *including literature search*, *source classification*, *data extraction and compilation*: Andrew Hacket‐Pain, Jessie J. Foest, Ian S. Pearse, Jalene M. LaMontagne, Walter D. Koenig, Giorgio Vacchiano, Michał Bogdziewicz, Thomas Caignard, Paulina Celebias, Joep van Dormolen, Marcos Fernández‐Martínez, Jose V. Moris, Ciprian Palaghianu, Mario Pesendorfer, Akiko Satake, Eliane Schermer, Andrew J. Tanentzap, Peter A. Thomas, Davide Vecchio, Andreas P. Wion, Thomas Wohlgemuth, Tingting Xue, Davide Ascoli. *Methodology*, *including data validation*: Andrew Hacket‐Pain, Jessie F. Foest. *Data Explorer*: Jessie J. Foest. *Data contribution*: Andrew Hacket‐Pain, Ian S. Pearse, Jalene M. LaMontagne, Walter D. Koenig, Michał Bogdziewicz, Peter A. Thomas, Katharine Abernethy, Marcelo Daniel Barrera, Jessica H. Barton, Stan Boutin, Emma R. Bush, Sergio Donoso Calderón, Felipe S. Carevic, Carolina Volkmer de Castilho, Juan Manuel Cellini, Hazel Chapman, Colin A. Chapman, Francesco Chianucci, Patricia da Costa, Luc Croisé, Andrea Cutini, Ben Dantzer, R. Justin DeRose, Jean‐Thoussaint Dikangadissi, Edmond Dimoto, Fernanda Lopes da Fonseca, Leonardo Gallo, Georg Gratzer, David F. Greene, Martín A. Hadad, Alejandro Huertas Herrera, Kathryn J. Jeffery, Jill F. Johnstone, Urs Kalbitzer, Władysław Kantorowicz, Christie A. Klimas, Jonathan G.A. Lageard, Jeffrey Lane, Katharina Lapin, Mateusz Ledwoń, Abigail Leeper, Maria Vanessa Lencinas, Ana Cláudia Lira‐Guedes, Michael C. Lordon, Paula Marchelli, Shealyn Marino, Harald Schmidt Van Marle, Andrew G. McAdam, Ludovic R.W. Momont, Manuel Nicolas, Lúcia Helena de Oliveira Wadt, Parisa Panahi, Guillermo Martínez Pastur, Thomas Patterson, Pablo Luis Peri, Łukasz Piechnik, Mehdi Pourhashemi, Claudia Espinoza Quezada, Fidel A. Roig, Karen Peña Rojas, Yamina Micaela Rosas, Silvio Schüler, Barbara Seget, Rosina Soler, Michael A. Steele, Mónica Toro‐Manríquez, Caroline E.G. Tutin, Tharcisse Ukizintambara, Lee White, Biplang Yadok, John L. Willis, Anita Zolles, Magdalena Żywiec, Davide Ascoli. *Writing—original draft*: Andrew Hacket‐Pain. *Writing—Review and editing*: All authors. *Supervision and Project administration*: Andrew Hacket‐Pain. *Funding acquisition*: Andrew Hacket‐Pain, Andrew J. Tanentzap, Peter A. Thomas.

## Supporting information

Appendix S1

Appendix S2

Appendix S3

Appendix S4

Appendix S5

Appendix S6

## Data Availability

The data that supports the findings of this study are available in the supplementary material of this article, and are openly available via Github: https://github.com/JJFoest/MASTREEplus, Dryad: https://doi.org/10.5061/dryad.18931zd02, or via the MASTREE+ Data Explorer: https://mastreeplus.shinyapps.io/mastreeplus

## References

[gcb16130-bib-0001] Abraham, E. M. , Sklavou, P. , Loufi, A. , Parissi, Z. M. , & Kyriazopoulos, A. P. (2018). The effect of combined herbivory by wild boar and small ruminants on the regeneration of a deciduous oak forest. Forests, 9(9), 580. 10.3390/f9090580

[gcb16130-bib-0002] Allen, C. D. , Macalady, A. K. , Chenchouni, H. , Bachelet, D. , McDowell, N. , Vennetier, M. , Kitzberger, T. , Rigling, A. , Breshears, D. D. , Hogg, E. H. (Ted) , Gonzalez, P. , Fensham, R. , Zhang, Z. , Castro, J. , Demidova, N. , Lim, J.‐H. , Allard, G. , Running, S. W. , Semerci, A. , & Cobb, N. (2010). A global overview of drought and heat‐induced tree mortality reveals emerging climate change risks for forests. Forest Ecology and Management, 259(4), 660–684. 10.1016/j.foreco.2009.09.001

[gcb16130-bib-0003] Arel‐Bundock, V. , Enevoldsen, N. , & Yetman, C. J. (2018). countrycode: An R package to convert country names and country codes. Journal of Open Source Software, 3(28), 848. 10.21105/joss.00848

[gcb16130-bib-0004] Ascoli, D. , Hacket‐Pain, A. , LaMontagne, J. M. , Cardil, A. , Conedera, M. , Maringer, J. , Motta, R. , Pearse, I. S. , & Vacchiano, G. (2020). Climate teleconnections synchronize Picea glauca masting and fire disturbance: Evidence for a fire‐related form of environmental prediction. Journal of Ecology, 108(3), 1186–1198. 10.1111/1365-2745.13308

[gcb16130-bib-0005] Ascoli, D. , Maringer, J. , Hacket‐Pain, A. , Conedera, M. , Drobyshev, I. , Motta, R. , Cirolli, M. , Kantorowicz, W. , Zang, C. , Schueler, S. , Croisé, L. , Piussi, P. , Berretti, R. , Palaghianu, C. , Westergren, M. , Lageard, J. G. A. , Burkart, A. , Gehrig Bichsel, R. , Thomas, P. A. , … Vacchiano, G. (2017). Two centuries of masting data for European beech and Norway spruce across the European continent. Ecology, 98(5), 1473. 10.1002/ecy.1785 28241388

[gcb16130-bib-0006] Ascoli, D. , Vacchiano, G. , Turco, M. , Conedera, M. , Drobyshev, I. , Maringer, J. , Motta, R. , & Hacket‐Pain, A. (2017). Inter‐annual and decadal changes in teleconnections drive continental‐scale synchronization of tree reproduction. Nature Communications, 8. 10.1038/s41467-017-02348-9 PMC5738406 29263383

[gcb16130-bib-0007] Bajocco, S. , Ferrara, C. , Bascietto, M. , Alivernini, A. , Chirichella, R. , Cutini, A. , & Chianucci, F. (2021). Characterizing the climatic niche of mast seeding in beech: Evidences of trade‐offs between vegetation growth and seed production. Ecological Indicators, 121, 9. 10.1016/j.ecolind.2020.107139

[gcb16130-bib-0008] Bennett, E. , Clement, J. , Sansom, P. , Hall, I. , Leach, S. , & Medlock, J. M. (2010). Environmental and ecological potential for enzootic cycles of *Puumala hantavirus* in Great Britain. Epidemiology and Infection, 138(1), 91–98. 10.1017/s095026880999029x 19563697

[gcb16130-bib-0009] Bogdziewicz, M. (2021). How will global change affect plant reproduction? A framework for mast seeding trends. New Phytologist, Early View. 10.1111/nph.17682 34409608

[gcb16130-bib-0010] Bogdziewicz, M. , Hacket‐Pain, A. , Ascoli, D. , & Szymkowiak, J. (2021). Environmental variation drives continental‐scale synchrony of European beech reproduction. Ecology, 102(7), 10. 10.1002/ecy.3384 33950521

[gcb16130-bib-0011] Bogdziewicz, M. , Kelly, D. , Thomas, P. A. , Lageard, J. G. A. , & Hacket‐Pain, A. (2020). Climate warming disrupts mast seeding and its fitness benefits in European beech. Nature Plants, 6(2), 88–94. 10.1038/s41477-020-0592-8 32042155

[gcb16130-bib-0012] Bogdziewicz, M. , Szymkowiak, J. , Fernández‐Martínez, M. , Peñuelas, J. , & Espelta, J. M. (2019). The effects of local climate on the correlation between weather and seed production differ in two species with contrasting masting habit. Agricultural and Forest Meteorology, 268, 109–115. 10.1016/j.agrformet.2019.01.016

[gcb16130-bib-0013] Bouchard, M. , Regniere, J. , & Therrien, P. (2018). Bottom‐up factors contribute to large‐scale synchrony in spruce budworm populations. Canadian Journal of Forest Research, 48(3), 277–284. 10.1139/cjfr-2017-0051

[gcb16130-bib-0014] Boutin, S. , Wauters, L. A. , McAdam, A. G. , Humphries, M. M. , Tosi, G. , & Dhondt, A. A. (2006). Anticipatory reproduction and population growth in seed predators. Science, 314(5807), 1928–1930. 10.1126/science.1135520 17185600

[gcb16130-bib-0015] Bregnard, C. , Rais, O. , & Voordouw, M. J. (2020). Climate and tree seed production predict the abundance of the European Lyme disease vector over a 15‐year period. Parasites & Vectors, 13(1). 10.1186/s13071-020-04291-z PMC7418309 32778177

[gcb16130-bib-0016] Brugger, K. , Walter, M. , Chitimia‐Dobler, L. , Dobler, G. , & Rubel, F. (2018). Forecasting next season's Ixodes ricinus nymphal density: The example of southern Germany 2018. Experimental and Applied Acarology, 75(3), 281–288. 10.1007/s10493-018-0267-6 29846854 PMC6097749

[gcb16130-bib-0017] Brumme, R. , Ahrends, B. , Block, J. , Schulz, C. , Meesenburg, H. , Klinck, U. , Wagner, M. , & Khanna, P. K. (2021). Cycling and retention of nitrogen in European beech (*Fagus sylvatica* L.) ecosystems under elevated fructification frequency. Biogeosciences, 18(12), 3763–3779. 10.5194/bg-18-3763-2021

[gcb16130-bib-0018] Buechling, A. , Martin, P. H. , Canham, C. D. , Shepperd, W. D. , & Battaglia, M. A. (2016). Climate drivers of seed production in *Picea engelmannii* and response to warming temperatures in the southern Rocky Mountains. Journal of Ecology, 104(4), 1051–1062. 10.1111/1365-2745.12572

[gcb16130-bib-0019] Buras, A. , Rammig, A. , & Zang, C. S. (2020). Quantifying impacts of the 2018 drought on European ecosystems in comparison to 2003. Biogeosciences, 17(6), 1655–1672. 10.5194/bg-17-1655-2020

[gcb16130-bib-0020] Bush, E. R. , Whytock, R. C. , Bahaa‐el‐din, L. , Bourgeois, S. , Bunnefeld, N. , Cardoso, A. W. , Dikangadissi, J. T. , Dimbonda, P. , Dimoto, E. , Edzang Ndong, J. , Jeffery, K. J. , Lehmann, D. , Makaga, L. , Momboua, B. , Momont, L. R. W. , Tutin, C. E. G. , White, L. J. T. , Whittaker, A. , & Abernethy, K. (2020). Long‐term collapse in fruit availability threatens Central African forest megafauna. Science, 370(6521), 1219–1221. 10.1126/science.abc7791 32972990

[gcb16130-bib-0021] Caignard, T. , Kremer, A. , Firmat, C. , Nicolas, M. , Venner, S. , & Delzon, S. (2017). Increasing spring temperatures favor oak seed production in temperate areas. Scientific Reports, 7, 8. 10.1038/s41598-017-09172-7 28819191 PMC5561138

[gcb16130-bib-0022] Calama, R. , Mutke, S. , Tome, J. , Gordo, J. , Montero, G. , & Tome, M. (2011). Modelling spatial and temporal variability in a zero‐inflated variable: The case of stone pine (*Pinus pinea* L.) cone production. Ecological Modelling, 222(3), 606–618. 10.1016/j.ecolmodel.2010.09.020

[gcb16130-bib-0023] Carnicer, J. , Coll, M. , Ninyerola, M. , Pons, X. , Sanchez, G. , & Penuelas, J. (2011). Widespread crown condition decline, food web disruption, and amplified tree mortality with increased climate change‐type drought. Proceedings of the National Academy of Sciences of the United States of America, 108(4), 1474–1478. 10.1073/pnas.1010070108 21220333 PMC3029725

[gcb16130-bib-0024] Cayuela, L. , Macarro, I. , Stein, A. , & Oksanen, J. (2021). Taxonstand: Taxonomic standardization of plant species names. Retrieved From https://CRAN.R‐project.org/package=Taxonstand

[gcb16130-bib-0025] Chang, W. , Cheng, J. , Allaire, J. J. , Sievert, C. , Schloerke, B. , Xie, Y. , … Borges, B. (2021). shiny: Web application framework for R (Version 1.6.0). Retrieved from https://CRAN.R‐project.org/package=shiny

[gcb16130-bib-0026] Changenet, A. , Ruiz‐Benito, P. , Ratcliffe, S. , Fréjaville, T. , Archambeau, J. , Porte, A. J. , Zavala, M. A. , Dahlgren, J. , Lehtonen, A. , & Benito Garzón, M. (2021). Occurrence but not intensity of mortality rises towards the climatic trailing edge of tree species ranges in European forests. Global Ecology and Biogeography, 30(7), 1356–1374. 10.1111/geb.13301

[gcb16130-bib-0027] Chen, J. M. , Ju, W. M. , Ciais, P. , Viovy, N. , Liu, R. G. , Liu, Y. , & Lu, X. H. (2019). Vegetation structural change since 1981 significantly enhanced the terrestrial carbon sink. Nature Communications, 10. 10.1038/s41467-019-12257-8 PMC6751163 31534135

[gcb16130-bib-0028] Chiavetta, U. , & Marzini, S. (2021). foreMast: an R package for predicting beech (*Fagus sylvatica* L.) masting events in European countries. Annals of Forest Science, 78(4), 10. 10.1007/s13595-021-01109-5

[gcb16130-bib-0029] Choquenot, D. , & Ruscoe, W. A. (2000). Mouse population eruptions in New Zealand forests: the role of population density and seedfall. Journal of Animal Ecology, 69(6), 1058–1070. 10.1046/j.1365-2656.2000.00462.x

[gcb16130-bib-0030] Clark, J. S. , Andrus, R. , Aubry‐Kientz, M. , Bergeron, Y. , Bogdziewicz, M. , Bragg, D. C. , Brockway, D. , Cleavitt, N. L. , Cohen, S. , Courbaud, B. , Daley, R. , Das, A. J. , Dietze, M. Fahey, T. J. , Fer, I. , Franklin, J. F. , Gehring, C. A. , Gilbert, G. S. , Greenberg, C. H. , … Zlotin, R. (2021). Continent‐wide tree fecundity driven by indirect climate effects. Nature Communications, 12(1), 1242. 10.1038/s41467-021-22025-2 PMC7902660 33623042

[gcb16130-bib-0031] Clark, J. S. , Iverson, L. , Woodall, C. W. , Allen, C. D. , Bell, D. M. , Bragg, D. C. , D'Amato, A. W. , Davis, F. W. , Hersh, M. H. , Ibanez, I. , Jackson, S. T. , Matthews, S. , Pederson, N. , Peters, M. , Schwartz, M. W. , Waring, K. M. , & Zimmermann, N. E. (2016). The impacts of increasing drought on forest dynamics, structure, and biodiversity in the United States. Global Change Biology, 22(7), 2329–2352. 10.1111/gcb.13160 26898361

[gcb16130-bib-0032] Connell, J. H. , & Green, P. T. (2000). Seedling dynamics over thirty‐two years in a tropical rain forest tree. Ecology, 81(2), 568–584. 10.1890/0012-9658(2000)081%5b0568:sdotty%5d2.0.co;2

[gcb16130-bib-0033] Cunze, S. , Kochmann, J. , Kuhn, T. , Frank, R. , Dorge, D. D. , & Klimpel, S. (2018). Spatial and temporal patterns of human Puumala virus (PUUV) infections in Germany. Peerj, 6, 20. 10.7717/peerj.4255 PMC5797684 29404206

[gcb16130-bib-0034] Curran, L. M. , & Leighton, M. (2000). Vertebrate responses to spatiotemporal variation in seed production of mast‐fruiting Dipterocarpaceae. Ecological Monographs, 70(1), 101–128. 10.1890/0012-9615(2000)070%5b0101:VRTSVI%5d2.0.CO;2

[gcb16130-bib-0035] Dale, E. E. , Foest, J. J. , Hacket‐Pain, A. , Bogdziewicz, M. , & Tanentzap, A. J. (2021). Macroevolutionary consequences of mast seeding. Philosophical Transactions of the Royal Society, 376, 2020372. 10.1098/rstb.2020.0372 PMC8520783 34657467

[gcb16130-bib-0036] Fargione, J. , Haase, D. L. , Burney, O. T. , Kildisheva, O. A. , Edge, G. , Cook‐Patton, S. C. , Chapman, T. , Rempel, A. , Hurteau, M. D. , Davis, K. T. , Dobrowski, S. , Enebak, S. , De La Torre, R. , Bhuta, A. A. R. , Cubbage, F. , Kittler, B. , Zhang, D. , & Guldin, R. W. (2021). Challenges to the reforestation pipeline in the United States. Frontiers in Forests and Global Change, 4, 18. 10.3389/ffgc.2021.629198

[gcb16130-bib-0037] Fernández‐Martínez, M. , Pearse, I. , Sardans, J. , Sayol, F. , Koenig, W. D. , LaMontagne, J. M. , Bogdziewicz, M. , Collalti, A. , Hacket‐Pain, A. , Vacchiano, G. , Espelta, J. M. , Peñuelas, J. , & Janssens, I. A. (2019). Nutrient scarcity as a selective pressure for mast seeding. Nature Plants, 5(12), 1222. 10.1038/s41477-019-0549-y 31792395

[gcb16130-bib-0038] Fernandez‐Pascual, E. (2021). SylvanSeeds, a seed germination database for temperate deciduous forests. Journal of Vegetation Science, 32, e12960. 10.1111/jvs.12960

[gcb16130-bib-0039] Fisher, R. A. , Koven, C. D. , Anderegg, W. R. L. , Christoffersen, B. O. , Dietze, M. C. , Farrior, C. E. , Holm, J. A. , Hurtt, G. C. , Knox, R. G. , Lawrence, P. J. , Lichstein, J. W. , Longo, M. , Matheny, A. M. , Medvigy, D. , Muller‐Landau, H. C. , Powell, T. L. , Serbin, S. P. , Sato, H. , Shuman, J. K. , … Moorcroft, P. R. (2018). Vegetation demographics in Earth System Models: A review of progress and priorities. Global Change Biology, 24(1), 35–54. 10.1111/gcb.13910 28921829

[gcb16130-bib-0040] Fujiki, D. (2018). Can frequent occurrence of Asiatic black bears around residential areas be predicted by a model‐based mast production in multiple Fagaceae species? Journal of Forest Research, 23(5), 260–269. 10.1080/13416979.2018.1488653

[gcb16130-bib-0041] Hacket‐Pain, A. , & Bogdziewicz, M. (2021). Climate change and plant reproduction: trends and drivers of mast seeding change. Philosophical Transactions of the Royal Society B, 376, 20200379. 10.1098/rstb.2020.0379 PMC8520772 34657461

[gcb16130-bib-0042] Heyman, P. , Thoma, B. R. , Marié, J.‐L. , Cochez, C. , & Essbauer, S. S. (2012). In search for factors that drive hantavirus epidemics. Frontiers in Physiology, 3, 237. 10.3389/fphys.2012.00237 22934002 PMC3429022

[gcb16130-bib-0043] Ida, H. (2021). A 15‐year study on the relationship between beech (*Fagus crenata*) reproductive‐organ production and the numbers of nuisance Japanese black bears (*Ursus thibetanus japonicus*) killed in a snowy rural region in central Japan. Landscape and Ecological Engineering, 17(4), 507–514. 10.1007/s11355-021-00472-9

[gcb16130-bib-0044] Joubert, D. F. , Smit, G. N. , & Hoffman, M. T. (2013). The influence of rainfall, competition and predation on seed production, germination and establishment of an encroaching Acacia in an arid Namibian savanna. Journal of Arid Environments, 91, 7–13. 10.1016/j.jaridenv.2012.11.001

[gcb16130-bib-0045] Kanamori, T. , Kuze, N. , Bernard, H. , Malim, T. P. , & Kohshima, S. (2017). Fluctuations of population density in Bornean orangutans (*Pongo pygmaeus morio*) related to fruit availability in the Danum Valley, Sabah, Malaysia: A 10‐year record including two mast fruitings and three other peak fruitings. Primates, 58(1), 225–235. 10.1007/s10329-016-0584-5 27848156

[gcb16130-bib-0046] Kelly, D. , Geldenhuis, A. , James, A. , Penelope Holland, E. , Plank, M. J. , Brockie, R. E. , Cowan, P. E. , Harper, G. A. , Lee, W. G. , Maitland, M. J. , Mark, A. F. , Mills, J. A. , Wilson, P. R. , & Byrom, A. E. (2013). Of mast and mean: Differential‐temperature cue makes mast seeding insensitive to climate change. Ecology Letters, 16(1), 90–98. 10.1111/ele.12020 23113938

[gcb16130-bib-0047] Kettle, C. J. , Ghazoul, J. , Ashton, P. S. , Cannon, C. H. , Chong, L. , Diway, B. , … Hollingsworth, P. (2010). Mass fruiting in Borneo: A missed opportunity. Science, 330(6004), 584. 10.1126/science.330.6004.584-a 21030629

[gcb16130-bib-0048] Khanna, P. K. , Fortmann, H. , Meesenburg, H. , Eichhorn, J. , & Meiwes, K. J. (2009). Biomass and element content of foliage and aboveground litterfall on the three long‐term experimental beech sites: Dynamics and significance. In R. Brumme & P. K. Khanna (Eds.), Functioning and management of European beech ecosystems (Vol. 208, pp. 183–205). Springer.

[gcb16130-bib-0049] Kleef, H. L. , & Wijsman, H. (2015). Mast, mice and pine marten (*Martes martes*): the pine marten's reproductive response to wood mouse (*Apodemus sylvaticus*) fluctuations in the Netherlands. Lutra, 58, 23–33.

[gcb16130-bib-0050] Klesse, S. , DeRose, R. J. , Babst, F. , Black, B. A. , Anderegg, L. D. L. , Axelson, J. , Ettinger, A. , Griesbauer, H. , Guiterman, C. H. , Harley, G. , Harvey, J. E. , Lo, Y.‐H. , Lynch, A. M. , O'Connor, C. , Restaino, C. , Sauchyn, D. , Shaw, J. D. , Smith, D. J. , Wood, L. , … Evans, M. E. K. (2020). Continental‐scale tree‐ring‐based projection of Douglas‐fir growth: Testing the limits of space‐for‐time substitution. Global Change Biology, 26(9), 5146–5163. 10.1111/gcb.15170 32433807

[gcb16130-bib-0051] Koenig, W. D. , & Knops, J. M. H. (2000). Patterns of annual seed production by northern hemisphere trees: A global perspective. The American Naturalist, 155(1), 59–69. 10.1086/303302 10657177

[gcb16130-bib-0052] Koenig, W. D. , & Knops, J. M. H. (2013). Large‐scale spatial synchrony and cross‐synchrony in acorn production by two California oaks. Ecology, 94(1), 83–93. 10.1890/12-0940.1 23600243

[gcb16130-bib-0053] Ladio, A. H. , & Lozada, M. (2004). Patterns of use and knowledge of wild edible plants in distinct ecological environments: A case study of a Mapuche community from northwestern Patagonia. Biodiversity and Conservation, 13(6), 1153–1173. 10.1023/b:bioc.0000018150.79156.50

[gcb16130-bib-0054] LaMontagne, J. M. , Pearse, I. S. , Greene, D. F. , & Koenig, W. D. (2020). Mast seeding patterns are asynchronous at a continental scale. Nature Plants, 6(5), 460. 10.1038/s41477-020-0647-x 32341539

[gcb16130-bib-0055] LaMontagne, J. M. , Redmond, M. , Greene, D. , & Koenig, W. D. (2021). An assessment of temporal variability in mast seeding of North American Pinaceae. Philosophical Transactions of the Royal Society B, 376, 20200373. 10.1098/rstb.2020.0373 PMC8520784 34657469

[gcb16130-bib-0056] Lauder, J. D. , Moran, E. V. , & Hart, S. C. (2019). Fight or flight? Potential tradeoffs between drought defense and reproduction in conifers. Tree Physiology, 39(7), 1071–1085. 10.1093/treephys/tpz031 30924877

[gcb16130-bib-0057] Lithner, S. , & Jönsson, I. (2002). Abundance of owls and Bramblings *Fringilla montifringilla* in relation to mast seeding in south‐eastern Sweden. Ornis Svecica, 12(1), 35–45.

[gcb16130-bib-0058] Manríquez, M. T. , Mestre, L. , Lencinas, M. V. , Promis, Á. , Pastur, G. M. , & Soler, R. (2016). Flowering and seeding patterns in pure and mixed Nothofagus forests in Southern Patagonia. Ecological Processes, 5(1), 21. 10.1186/s13717-016-0065-1

[gcb16130-bib-0059] Martin‐DeMoor, J. , Lieffers, V. J. , & Macdonald, S. E. (2010). Natural regeneration of white spruce in aspen‐dominated boreal mixedwoods following harvesting. Canadian Journal of Forest Research‐Revue Canadienne De Recherche Forestiere, 40(3), 585–594. 10.1139/x10-016

[gcb16130-bib-0060] McDowell, N. G. , Allen, C. D. , Anderson‐Teixeira, K. , Aukema, B. H. , Bond‐Lamberty, B. , Chini, L. , Clark, J. S. , Dietze, M. , Grossiord, C. , Hanbury‐Brown, A. , Hurtt, G. C. , Jackson, R. B. , Johnson, D. J. , Kueppers, L. , Lichstein, J. W. , Ogle, K. , Poulter, B. , Pugh, T. A. M. , Seidl, R. , … Xu, C. (2020). Pervasive shifts in forest dynamics in a changing world. Science, 368(6494), 964. 10.1126/science.aaz9463 32467364

[gcb16130-bib-0061] Mokake, S. E. , Chuyong, G. B. , Egbe, A. E. , Tabot, P. T. , Jumbam, B. , Biyon, B. J. N. , & Dibong, S. D. (2018). Plant reproductive phenology following selective logging in a semideciduous tropical forest in the East Region of Cameroon. Journal of Applied Biosciences, 128, 12901–12919. 10.4314/jab.v128i1.4

[gcb16130-bib-0062] Mosier, T. M. , Hill, D. F. , & Sharp, K. V. (2014). 30‐Arcsecond monthly climate surfaces with global land coverage. International Journal of Climatology, 34(7), 2175–2188. 10.1002/joc.3829

[gcb16130-bib-0063] Muller‐Haubold, H. , Hertel, D. , & Leuschner, C. (2015). Climatic drivers of mast fruiting in European beech and resulting C and N allocation shifts. Ecosystems, 18(6), 1083–1100. 10.1007/s10021-015-9885-6

[gcb16130-bib-0064] Mundo, I. A. , Sanguinetti, J. , & Kitzberger, T. (2021). Multi‐centennial phase‐locking between reproduction of a South American conifer and large‐scale drivers of climate. Nature Plants, 7(12), 1560. 10.1038/s41477-021-01038-1 34907311

[gcb16130-bib-0065] Nussbaumer, A. , Gessler, A. , Benham, S. , de Cinti, B. , Etzold, S. , Ingerslev, M. , Jacob, F. , Lebourgeois, F. , Levanic, T. , Marjanović, H. , Nicolas, M. , Ostrogović Sever, M. Z. , Priwitzer, T. , Rautio, P. , Roskams, P. , Sanders, T. G. M. , Schmitt, M. , Šrámek, V. , Thimonier, A. , … Rigling, A. (2021). Contrasting resource dynamics in mast years for European beech and oak—A continental scale analysis. Frontiers in Forests and Global Change, 4, 17. 10.3389/ffgc.2021.689836

[gcb16130-bib-0066] Oddou‐Muratorio, S. , Petit‐Cailleux, C. , Journé, V. , Lingrand, M. , Magdalou, J.‐A. , Hurson, C. , Garrigue, J. , Davi, H. , & Magnanou, E. (2021). Crown defoliation decreases reproduction and wood growth in a marginal European beech population. Annals of Botany, 128(2), 193–204. 10.1093/aob/mcab054 33928352 PMC8324029

[gcb16130-bib-0067] O'Donnell, C. F. J. , & Hoare, J. M. (2012). Quantifying the benefits of long‐term integrated pest control for forest bird populations in a New Zealand temperate rainforest. New Zealand Journal of Ecology, 36(2), 131–140.

[gcb16130-bib-0068] Oliva, G. , Collantes, M. , & Humano, G. (2013). Reproductive effort and seed establishment in grazed tussock grass populations of Patagonia. Rangeland Ecology & Management, 66(2), 164–173. 10.2111/REM-D-11-00121.1

[gcb16130-bib-0069] Ostfeld, R. S. , Jones, C. G. , & Wolff, J. O. (1996). Of mice and mast. BioScience, 46(5), 323–330. 10.2307/1312946

[gcb16130-bib-0070] Pau, S. , Okamoto, D. K. , Calderon, O. , & Wright, S. J. (2018). Long‐term increases in tropical flowering activity across growth forms in response to rising CO_2_ and climate change. Global Change Biology, 24(5), 2105–2116. 10.1111/gcb.14004 29265499

[gcb16130-bib-0071] Pearse, I. S. , LaMontagne, J. M. , & Koenig, W. D. (2017). Inter‐annual variation in seed production has increased over time (1900–2014). Proceedings of the Royal Society B‐Biological Sciences, 284(1868), 1900–2014. 10.1098/rspb.2017.1666 PMC5740272 29212721

[gcb16130-bib-0072] Pearse, I. S. , LaMontagne, J. M. , Lordon, M. , Hipp, A. L. , & Koenig, W. D. (2020). Biogeography and phylogeny of masting: Do global patterns fit functional hypotheses? New Phytologist, 227(5), 1557–1567. 10.1111/nph.16617 32315447

[gcb16130-bib-0073] Pearse, I. S. , Wion, A. , Gonzalez, A. , & Pesendorfer, M. B. (2021). Understanding masting for conservation and land management. Philosophical Transactions of the Royal Society B, 376, 20200383. 10.1098/rstb.2020.0383 PMC8520776 34657466

[gcb16130-bib-0074] Pennekamp, F. , Iles, A. C. , Garland, J. , Brennan, G. , Brose, U. , Gaedke, U. , Jacob, U. , Kratina, P. , Matthews, B. , Munch, S. , Novak, M. , Palamara, G. M. , Rall, B. C. , Rosenbaum, B. , Tabi, A. , Ward, C. , Williams, R. , Ye, H. , & Petchey, O. L. (2019). The intrinsic predictability of ecological time series and its potential to guide forecasting. Ecological Monographs, 89(2). 10.1002/ecm.1359

[gcb16130-bib-0075] Pesendorfer, M. B. , Ascoli, D. , Bogdziewicz, M. , Hacket‐Pain, A. , Pearse, I. S. , & Vacchiano, G. (2021). The ecology and evolution of synchronized reproduction in long‐lived plants. Philosophical Transactions of the Royal Society B, 376, 20200369. 10.1098/rstb.2020.0369 PMC8520778 34657462

[gcb16130-bib-0076] Peters, V. S. , MacDonald, S. E. , & Dale, M. R. T. (2005). The interaction between masting and fire is key to white spruce regeneration. Ecology, 86(7), 1744–1750. 10.1890/03-0656

[gcb16130-bib-0077] Pukkala, T. , Hokkanen, T. , & Nikkanen, T. (2010). Prediction models for the annual seed crop of Norway spruce and scots pine in Finland. Silva Fennica, 44(4), 629–642. 10.14214/sf.131

[gcb16130-bib-0078] Redmond, M. D. , Davis, T. S. , Ferrenberg, S. , & Wion, A. P. (2019). Resource allocation trade‐offs in a mast‐seeding conifer: Pinon pine prioritizes reproduction over defence. Aob Plants, 11(6), plz070. 10.1093/aobpla/plz070

[gcb16130-bib-0079] Redmond, M. D. , Forcella, F. , & Barger, N. N. (2012). Declines in pinyon pine cone production associated with regional warming. Ecosphere, 3(12), 14. 10.1890/es12-00306.1

[gcb16130-bib-0080] Redmond, M. D. , Weisberg, P. J. , Cobb, N. S. , & Clifford, M. J. (2018). Woodland resilience to regional drought: Dominant controls on tree regeneration following overstorey mortality. Journal of Ecology, 106(2), 625–639. 10.1111/1365-2745.12880

[gcb16130-bib-0081] Rohatgi, A. (2020). WebPlotDigitizer (Version 4.4). Retrieved from http://arohatgi.info/WebPlotDigitizer

[gcb16130-bib-0082] Royal Botanic Gardens Kew . (2021). *Seed Information Database (SID)* .

[gcb16130-bib-0083] Ruiz‐Benito, P. , Ratcliffe, S. , Zavala, M. A. , Martínez‐Vilalta, J. , Vilà‐Cabrera, A. , Lloret, F. , Madrigal‐González, J. , Wirth, C. , Greenwood, S. , Kändler, G. , Lehtonen, A. , Kattge, J. , Dahlgren, J. , & Jump, A. S. (2017). Climate‐ and successional‐related changes in functional composition of European forests are strongly driven by tree mortality. Global Change Biology, 23(10), 4162–4176. 10.1111/gcb.13728 28418105

[gcb16130-bib-0084] Salguero‐Gomez, R. , Jones, O. R. , Archer, C. R. , Buckley, Y. M. , Che‐Castaldo, J. , Caswell, H. , Hodgson, D. , Scheuerlein, A. , Conde, D. A. , Brinks, E. , de Buhr, H. , Farack, C. , Gottschalk, F. Hartmann, A. , Henning, A. , Hoppe, G. , Römer, G. , Runge, J. , Ruoff, T. , … Vaupel, J. W. (2015). The COMPADRE Plant Matrix Database: An open online repository for plant demography. Journal of Ecology, 103(1), 202–218. 10.1111/1365-2745.12334

[gcb16130-bib-0085] Salguero‐Gómez, R. , Jones, O. R. , Jongejans, E. , Blomberg, S. P. , Hodgson, D. J. , Mbeau‐Ache, C. , Zuidema, P. A. , de Kroon, H. , & Buckley, Y. M. (2016). Fast‐slow continuum and reproductive strategies structure plant life‐history variation worldwide. Proceedings of the National Academy of Sciences of the United States of America, 113(1), 230–235. 10.1073/pnas.1506215112 26699477 PMC4711876

[gcb16130-bib-0086] Schupp, E. W. , Zwolak, R. , Jones, L. R. , Snell, R. S. , Beckman, N. G. , Aslan, C. , Cavazos, B. R. , Effiom, E. , Fricke, E. C. , Montaño‐Centellas, F. , Poulsen, J. , Razafindratsima, O. H. , Sandor, M. E. , & Shea, K. (2019). Intrinsic and extrinsic drivers of intraspecific variation in seed dispersal are diverse and pervasive. Aob Plants, 11(6), 20. 10.1093/aobpla/plz067 PMC6914678 31857875

[gcb16130-bib-0087] Selonen, V. , Wistbacka, R. , & Korpimaki, E. (2016). Food abundance and weather modify reproduction of two arboreal squirrel species. Journal of Mammalogy, 97(5), 1376–1384. 10.1093/jmammal/gyw096

[gcb16130-bib-0088] Senf, C. , Pflugmacher, D. , Zhiqiang, Y. , Sebald, J. , Knorn, J. , Neumann, M. , Hostert, P. , & Seidl, R. (2018). Canopy mortality has doubled in Europe's temperate forests over the last three decades. Nature Communications, 9. 10.1038/s41467-018-07539-6 PMC6255806 30478255

[gcb16130-bib-0089] Shelef, O. , Weisberg, P. J. , & Provenza, F. D. (2017). The value of native plants and local production in an era of global agriculture. Frontiers in Plant Science, 8. 10.3389/fpls.2017.02069 PMC5723411 29259614

[gcb16130-bib-0090] Touzot, L. , Schermer, É. , Venner, S. , Delzon, S. , Rousset, C. , Baubet, É. , Gaillard, J.‐M. , & Gamelon, M. (2020). How does increasing mast seeding frequency affect population dynamics of seed consumers? Wild boar as a case study. Ecological Applications, 30(6). 10.1002/eap.2134 32299142

[gcb16130-bib-0091] Vacchiano, G. , Ascoli, D. , Berzaghi, F. , Lucas‐Borja, M. E. , Caignard, T. , Collalti, A. , Mairota, P. , Palaghianu, C. , Reyer, C. P. O. , Sanders, T. G. M. , Schermer, E. , Wohlgemuth, T. , & Hacket‐Pain, A. (2018). Reproducing reproduction: How to simulate mast seeding in forest models. Ecological Modelling, 376, 40–53. 10.1016/j.ecolmodel.2018.03.004

[gcb16130-bib-0092] Whittaker, R. (1970). Communities and ecosystems. Macmillan.

[gcb16130-bib-0093] Wion, A. P. , Weisberg, P. J. , Pearse, I. S. , & Redmond, M. D. (2020). Aridity drives spatiotemporal patterns of masting across the latitudinal range of a dryland conifer. Ecography, 43(4), 569–580. 10.1111/ecog.04856

[gcb16130-bib-0094] Zhang, W. Y. , Wang, Y. , Xiao, J. Y. , & Lyu, L. X. (2022). Species‐specific coupling of tree‐ring width and litter production in a temperate mixed forest. Forest Ecology and Management, 504, 9. 10.1016/j.foreco.2021.119831

