# Supplementary material for: MASTREE+: Time‐series of plant reproductive effort from six continents
Source: Glob Chang Biol. 2022 Mar 5;28(9):3066–82. doi: 10.1111/gcb.16130 (PMC9314730; doi:10.1111/gcb.16130)
Supplement: Supplementary file 2 — Appendix S2 [file GCB-28-3066-s004.docx]

Appendix 2: Literature search for potential sources of masting data

**Identifying potential data sources**

To identify literature containing potential sources of data for MASTREE+ we performed literature searches using Google Scholar and Scopus. Google Scholar was used as it includes more comprehensive coverage of the grey literature (e.g. conference proceedings, theses, reports) which we discovered was a rich source of unpublished data on plant reproductive effort. Various combinations of search terms were used (Table A1). Efforts focused on sources published since 2015 as an earlier compilation effort by (Pearse, LaMontagne, and Koenig 2017) had been conducted on earlier records. For Google Scholar, search results were initially screened for relevance using the title and the opening words of the abstract presented on the search results page. This initial screen only excluded clearly irrelevant sources. The combined Google Scholar and Scopus searches resulted in an initial list of 2,476 potential sources of data.

Papers were allocated to a group of co-authors for classification based on their potential to contribute data to MASTREE+. PDF copies of all sources were saved by reviewing co-authors in a central SharePoint repository, and classified based on the system outlined in Table A2.

Data on the annual reproductive output for all perennial species was included, except for agricultural crops subject to selective breeding. All stages of reproductive output were included (e.g. flowers, mature fruits), and we included all potential measures of reproductive output, including direct measures (e.g. fruit counts, seed counts), and reconstructions (e.g. cone scars, dendrochronological reconstructions).

Data at the stand-, population- or regional-scale were included – i.e. assessments of annual reproductive output averaged across multiple individuals. Where data was provided at the individual plant level, we averaged to create a population mean.

Data for plants that were clearly identified as reproductively immature were excluded. We also excluded data from manipulation treats in experimental studies, although data from control treatments was included.

Other potential sources of data were added during the compilation basis on an ad-hoc basis, including from citations and sources of data referenced in manuscripts read during the review process, manuscripts published after the literature searches were conducted, and based on recommendations from colleagues.

Additionally, we compiled new data from a database of sources created by c-author Davide Ascoli during the compilation of MASTREE, which only published data for *Fagus sylvatica* and *Picea abies* (Ascoli et al. 2017).

**References**Ascoli, D., J. Maringer, A. Hacket-Pain, M. Conedera, I. Drobyshev, R. Motta, M. Cirolli, W. Kantorowicz, C. Zang, S. Schueler, L. Croise, P. Piussi, R. Berretti, C. Palaghianu, M. Westergren, J. G. A. Lageard, A. Burkart, R. G. Bichsel, P. A. Thomas, B. Beudert, R. Overgaard, and G. Vacchiano. 2017. "Two centuries of masting data for European beech and Norway spruce across the European continent." Ecology 98 (5):1473-+. doi: 10.1002/ecy.1785.

Pearse, I. S., J. M. LaMontagne, and W. D. Koenig. 2017. "Inter-annual variation in seed production has increased over time (1900-2014)." Proceedings of the Royal Society B-Biological Sciences 284 (1868). doi: 10.1098/rspb.2017.1666.

Table A1. Google Scholar and Scopus search terms

| **Google Scholar** | | | | |
| --- | --- | --- | --- | --- |
| **Search term (exact)** | **Date** | **Limitations** | **Results** | **Notes** |
| masting seed production | 21/01/2019 | Since 2018 | ~486 | Checked all results, 220 selected based on title and mini-abstract |
| masting AND "seed production" | 31/01/2019 | 2013-2017 | ~2460 | Checked first 1000, sorted by "relevance", very few additional potential sources so stopped |
| "hard mast" | 22/01/2019 | Since 2018 | ~78 | Checked all results |
| fruit production AND forest AND tree | 22/01/2019 | Since 2018 | ~2,070 | Checked first 300, sorted by "relevance", very few additional potential sources so stopped |
| tropical AND mast AND tree | 22/01/2019 | Since 2015 | ~6,100 | Checked first 600, sorted by "relevance", very few additional potential sources so stopped |
| "variable seed production" | 22/01/2019 | Since 2015 | 73 | Checked all results |
| "cone scar" OR "cone scars" | 22/01/2019 | All years | 114 | Checked all results |
| masting AND africa | 23/01//2019 | All years | ~2360 | Checked first 700, sorted by "relevance", very few additional potential sources so stopped |
| masting AND africa | 23/01//2019 | Since 2015 | ~539 | Checked all results |
| "mass seeding" | 23/01//2019 | All years | 288 | Checked all results |
| "mass fruiting" | 23/01//2019 | All years | 289 | Checked all results |
| "periodic flowering" | 23/01//2019 | All years | 339 | Checked all results |
| seed production AND forest | 24/01/2019 | Since 2014 | ~17,100 | Checked first 1000, sorted by "relevance", very few additional potential sources so stopped |
| cone crop | 24/01/2019 | Since 2014 | 326 | Checked all results |
| bumper AND seed AND tree | 28/01/2019 | Since 2014 | ~3070 | Checked first 220, sorted by "relevance", very few additional potential sources so stopped |
| "seed set" variability | 29/01/2019 | Since 2014 | ~7520 | Checked first 220, sorted by "relevance", very few additional potential sources so stopped |
| "sporadic seeding" OR "sporadic fruiting" OR "sporadic seed" OR "sporadic fruit" | 29/01/2019 | All years | ~174 | Checked all results |
| "seed trap" | 30/01/2019 | Since 2014 | ~445 | Checked all results |
| "seed trap" | 30/01/2019 | before 2014 | ~1750 | Checked first 600, sorted by "relevance", very few additional potential sources so stopped |
| seed trap AND mast | 30/01/2019 | All years | 385 | Checked all results |
| "seed rain" OR "fruit rain" AND forest OR tree OR grass | 31/01/2019 | Since 2014 | ~4160 | Checked first 600, sorted by "relevance", very few additional potential sources so stopped |
| mast event OR "mast events" | 13/02/2019 | All years | 566 | Checked first 300, sorted by "relevance", very few additional potential sources so stopped |
| **Scopus** | | | | |
| masting AND seed OR fruit | 31/01/2019 | All years | 509 | All imported, not pre-screened |
| TITLE-ABS-KEY ( *"inter-annual vari*"*  OR  *"interannual vari*"*  AND  *seed* )  AND  ( LIMIT-TO ( PUBYEAR ,  *2018* )  OR  LIMIT-TO ( PUBYEAR ,  *2017* )  OR  LIMIT-TO ( PUBYEAR ,  *2016* )  OR  LIMIT-TO ( PUBYEAR ,  *2015* )  OR  LIMIT-TO ( PUBYEAR ,  *2014* )  OR  LIMIT-TO ( PUBYEAR ,  *2013* ) ) | 31/01/2019 | Since 2013 | 92 | All imported, not pre-screened |
| TITLE-ABS-KEY ( *"inter-annual vari*"*  OR  *"interannual vari*"*  AND  *cone* )  AND  ( LIMIT-TO ( PUBYEAR ,  *2018* )  OR  LIMIT-TO ( PUBYEAR ,  *2017* )  OR  LIMIT-TO ( PUBYEAR ,  *2016* )  OR  LIMIT-TO ( PUBYEAR ,  *2015* )  OR  LIMIT-TO ( PUBYEAR ,  *2014* )  OR  LIMIT-TO ( PUBYEAR ,  *2013* ) ) | 31/01/2019 | Since 2013 | 8 | All imported, not pre-screened |

Table A2. Classification categories for potential sources of data for MASTREE+

| **Classification category** | **Code** |  |
| --- | --- | --- |
| **Useful** | **U** | The paper contained data which could be extracted from the PDF (Tables, Figures, text). This included annual assessments of seeds, fruits, inflorescences, flowers, cones or pollen, or any other measure of reproductive effort. Data could be continuous or ordinal, including references to “mast years”, e.g. “2005 was a heavy seed crop”.  Only data for observed reproductive effort were included, not predicted/expected/anticipated effort. |
| **Data online** | **O** | Useful data was available as an online supplement to the source, or in an online repository with a link in the paper. |
| **Data not provided** | **D** | Valuable data was discussed or analysed, but was not available in the source for extraction. Depending on the potential of the data, the MASTREE+ leading team contacted the authors directly to request data. |
| **Potentially useful references** | **R** | The source contained no useful data, but pointed to potentially other useful data sources. These were usually other references, particularly in the grey literature (unpublished theses, government reports etc.) |
| **No use** | **X** | The source was not useful, containing nothing useful for MASTREE+ |
| **Unavailable** | **N** | The source could not be accessed, and was therefore not checked during the initial screen. This included sources that are in a language that the reviewer could not read. The MASTREE+ leading team subsequently checked these sources. |
