# Supplementary material for: MASTREE+: Time‐series of plant reproductive effort from six continents
Source: Glob Chang Biol. 2022 Mar 5;28(9):3066–82. doi: 10.1111/gcb.16130 (PMC9314730; doi:10.1111/gcb.16130)
Supplement: Supplementary file 3 — Appendix S3 [file GCB-28-3066-s006.docx]

Appendix 3: Data cleaning and removal of duplicated time-series

A two-stage approach was adopted to validate compiled time-series data before their final inclusion in MASTREE+.

**Stage 1: Standardisation of attribute data and checking for errors and inconsistencies *within* time-series**

During the first stage, we standardised attribute data and checked for errors and inconsistencies *within* time-series.

To improve MASTREE+ wide consistency in attribute data, we standardised plant species names, country names, and reproductive output units. Species names were standardised to The Plant List (TPL) nomenclature, using the “Taxonstand” package (v. 2.3) (Cayuela et al. 2021). Country names were converted to the English short name (ISO3166-1), using the “countrycode” package (v. 1.2.0) (Arel-Bundock, Enevoldsen, and Yetman 2018). We converted the reproductive output (Value) of time-series with related Unit values into a common Unit type (e.g., we converted “seeds/ha” to “seeds/m2”).

For all variables (i.e., columns), we corrected spreadsheet autocompletion mistakes and erroneous missing values.

The core MASTREE+ variables, which include the identification (“Alpha_Number”, “Site_number”, “Variable_number”, “Species_code”) and the time-series variables (“Year”, “Value”), were subjected to various checks. We ensured time-series were uniquely identified by the identification variables, and that time-series’ observations were uniquely identified by “Year”. To ascertain that the appropriate “Species_code” was assigned, we automatically combined the first three characters from the TPL-standardised genus and species names, except for those instances where multiple species would fall under the same “Species_code” (in which case the final three characters of “Species_code” were manually changed to a unique combination) or where the plant population was of a hybrid origin (in which case the final character was changed to “X”). We inspected irregularities in the reproductive output variable “Value”. Manual checks were conducted on time-series where “Value” or its standard deviation fell below or equalled 0, respectively. For each time-series, the highest and lowest reproductive output values (0.05% < “Value” > 99.95% of the time-series’ reproductive output values) were inspected to help identify and correct data-entry related outliers.

We ensured all observations in a time-series had uniform attribute data where such uniformity was expected (i.e., within a time-series, there is only a single value for variables such as “Unit”). Uniformity was not required for: “No_indivs” and “Date_entry”. Interrelated variables were checked to ensure consistency; for example, the time-series spatial reference (“Latitude”, “Longitude”) was checked to ensure that it was located within the boundaries of the indicated “Country”. Time-series duration variables (i.e., “Segment”, “Start”, “End”, “Length”) were directly calculated from time-series.

**Stage 2: Detection and removal of duplicated time-series**

The second stage involved the detection and removal of duplication problems *between* time-series.

We first created ‘potential duplication groups’ (PDG). Each PDG contained a set of time-series that shared the same study species and approximate location. To determine if the spatial proximity requirement was met, we created spatial buffers (± 0.1 decimal degree) around the spatial reference coordinates for each time series and checked for overlap in coordinate ranges. This was initially completed with IRanges v 2.22.2 (Lawrence et al. 2013) (functions: IRanges, findOverlaps, and reduce), then, for all time-series within groups a pairwise check of coordinate overlap was conducted. This two-step procedure was required because IRanges can “chain” overlapping ranges together. PDGs which did not contain time-series from multiple sources (“Alpha_Number”) were not inspected further. Time-series pairs within each PDG which had fewer than 3 overlapping years were also filtered out. Similarly, if a pair of time-series within a PDG were not highly correlated (Spearman's ρ < 0.97), the pair was not treated as a potential duplicate. After the PDGs were filtered based on above criteria, the resulting set of PDGs were manually checked for duplication. Where necessary, the original data source of time-series was examined to identify signs of duplication (e.g., the methods used for both series matched exactly, or the other source was referenced as the data contributor).

To supplement the automated detection of duplicates, we also examined a version of the dataset which contained only a limited set of variables, namely “Species”, “Country”, “Reference”, “Site”, “Latitude”, “Longitude”, as well as the range of “Year” values present in each time-series. The rows of this version were sorted by “Country”, “Species”, “Latitude” and “Longitude”, in that order. Each section of rows that shared their values for “Country” and “Species” was inspected for signs of duplication. Such signs would include exactly matching spatial references, matching site descriptions, and matching author names.

Upon identifying duplicates, we selected only a single time-series for inclusion in MASTREE+. Generally, the longest time-series was prioritised, unless there were clear signs that a shorter time-series was of higher quality (e.g., the data was directly shared by the author and not extracted from a graph), or of higher research value (e.g., the time-series’ Value unit was continuous, not ordinal).

**References**

Arel-Bundock, V., N. Enevoldsen, and C.J. Yetman. 2018. "countrycode: An R package to convert country names and country codes." Journal of Open Source Software 3 (28):848. doi: <https://doi.org/10.21105/joss.00848>

Cayuela, L., Macarro, I., Stein, A., Oksanen, J. 2021. Taxonstand: Taxonomic Standardization of Plant Species Names. https://CRAN.R-project.org/package=Taxonstand.

Lawrence, M., W. Huber, H. Pages, P. Aboyoun, M. Carlson, R. Gentleman, M. T. Morgan, and V. J. Carey. 2013. "Software for Computing and Annotating Genomic Ranges." Plos Computational Biology 9 (8). doi: 10.1371/journal.pcbi.1003118.
