# Supplementary material for: MASTREE+: Time‐series of plant reproductive effort from six continents
Source: Glob Chang Biol. 2022 Mar 5;28(9):3066–82. doi: 10.1111/gcb.16130 (PMC9314730; doi:10.1111/gcb.16130)
Supplement: Supplementary file 4 — Appendix S4 [file GCB-28-3066-s003.docx]

**Appendix 4: List of references included in the MASTREE+ dataset**

| **Alpha_Number** | **Reference** |
| --- | --- |
| 0001 | Abbas, F., N. Morellet, A. J. M. Hewison, J. Merlet, B. Cargnelutti, B. Lourtet, J. M. Angibault, T. Daufresne, S. Aulagnier, and H. Verheyden. 2011. Landscape fragmentation generates spatial variation of diet composition and quality in a generalist herbivore. Oecologia 167:401-411. |
| 0003 | Abe, M., H. Miguchi, A. Honda, A. Makita, and T. Nakashizuka. 2005. Short-term changes affecting regeneration of Fagus crenata after the simultaneous death of Sasa kurilensis. Journal of Vegetation Science 16:49-56. |
| 0004 | Abe, Y., and S. Shibata. 2014. Flower and seed production in a series of flowerings from sporadic events before to after mass flowering of the dwarf bamboo Sasa veitchii var. hirsuta. Journal of forest research 19:268-275. |
| 0005 | Abe, T., Y. Tachiki, H. Kon, A. Nagasaka, K. Onodera, K. Minamino, Q. M. Han, and A. Satake. 2016. Parameterisation and validation of a resource budget model for masting using spatiotemporal flowering data of individual trees. Ecology Letters 19:1129-1139. |
| 0006 | Abe, Y., and S. Shibata. 2012. Spatial and temporal flowering patterns of the monocarpic dwarf bamboo Sasa veitchii var. hirsuta. Ecological research 27:625-632. |
| 0009 | Abraham, E. M., P. Sklavou, A. Loufi, Z. M. Parissi, and A. P. Kyriazopoulos. 2018. The Effect of Combined Herbivory by Wild Boar and Small Ruminants on the Regeneration of a Deciduous Oak Forest. Forests 9:10. |
| 0013 | Abrams, M. D., and M. S. Scheibel. 2013. A Five-year Record Mast Production and Climate in Contrasting Mixed-oak-hickory Forests on the Mashomack Preserve, Long Island, New York, USA. Natural Areas Journal 33:99-104. |
| 0023 | Akashi, N. 1997. Dispersion pattern and mortality of seeds and seedlings of Fagus crenata Blume in a cool temperate forest in western Japan. Ecological Research 12:159-165. |
| 0025 | Akita, T., K. Sakai, Y. Iwabuchi, Y. Hoshino, and X. Ye. 2008. Spatial autocorrelation in masting phenomena of Quercus serrata detected by multi-spectral imaging. Ecological Modelling 215:217-224. |
| 0026 | Alatalo, J. M., and C. J. Little. 2014. Simulated global change: contrasting short and medium term growth and reproductive responses of a common alpine/Arctic cushion plant to experimental warming and nutrient enhancement. SpringerPlus 3:157. |
| 0030 | Alejano, R., R. Tapias, M. Fernández, E. Torres, J. Alaejos, and J. Domingo. 2008. Influence of pruning and the climatic conditions on acorn production in holm oak (Quercus ilex L.) dehesas in SW Spain. Annals of Forest Science 65. |
| 0035 | Alfonso-Corrado, C., R. Clark-Tapia, and A. Mendoza. 2007. Demography and management of two clonal oaks: Quercus eduardii and Q-potosina (Fagaceae) in central Mexico. Forest Ecology and Management 251:129-141. |
| 0046 | Alvarez-Aquino, C., and G. Williams-Linera. 2002. Seedling bank dynamics of Fagus grandifolia var. mexicana before and after a mast year in a Mexican cloud forest. Journal of Vegetation Science 13:179-184. |
| 0089 | Azad, S., T. Wactor, and D. Jachowski. 2017. Relationship of Acorn Mast Production to Black Bear Population Growth Rates and Human-Bear Interactions in Northwestern South Carolina. Southeastern Naturalist 16:235-251. |
| 0106 | Barker, P. C. J. 1995. PHYLLOCLADUS ASPLENIIFOLIUS - PHENOLOGY, GERMINATION, AND SEEDLING SURVIVAL. New Zealand Journal of Botany 33:325-337. |
| 0111 | Bebber, D. P., N. D. Brown, and M. R. Speight. 2004. Dipterocarp seedling population dynamics in Bornean primary lowland forest during the 1997-8 El Nino-Southern Oscillation. Journal of Tropical Ecology 20:11-19. |
| 0112 | Beckage, B., J. S. Clark, B. D. Clinton, and B. L. Haines. 2000. A long-term study of tree seedling recruitment in southern Appalachian forests: the effects of canopy gaps and shrub understories. Canadian Journal of Forest Research 30:1617-1631. |
| 0125 | Berjano, R., M. Arista, P. Ortiz, and S. Talavera. 2011. Persistently low fruiting success in the Mediterranean pipevine Aristolochia baetica (Aristolochiaceae): a multi‐year study. Plant Biology 13:109-117. |
| 0152 | Bogdziewicz, M., J. M. Espelta, A. Munoz, J. M. Aparicio, and R. Bonal. 2018. Effectiveness of predator satiation in masting oaks is negatively affected by conspecific density. Oecologia 186:983-993. |
| 0162 | Boieiro, M. R. C. 2014. Spatio-temporal variation in seed production in three Euphorbia species and the role of animals on seed fate. |
| 0171 | Bondé, L., O. Ouédraogo, I. Ouédraogo, A. Thiombiano, and J. I. Boussim. 2018. Variability and estimating in fruiting of shea tree (Vitellaria paradoxa CF Gaertn) associated to climatic conditions in West Africa: implications for sustainable management and development. Plant Production Science:1-16. |
| 0187 | Boulanger‐Lapointe, N., A. Järvinen, R. Partanen, and T. M. Herrmann. 2017. Climate and herbivore influence on Vaccinium myrtillus over the last 40 years in northwest Lapland, Finland. Ecosphere 8. |
| 0196 | Brandl, R., E. Bezzel, J. Reichholf, and W. Volkl. 1991. POPULATION-DYNAMICS OF THE RED SQUIRREL IN BAVARIA. Zeitschrift Fur Saugetierkunde-International Journal of Mammalian Biology 56:10-18. |
| 0201 | Brearley, F. Q., J. Proctor, Suriantata, L. Nagy, G. Dalrymple, and B. C. Voysey. 2007. Reproductive phenology over a 10-year period in a lowland evergreen rain forest of central Borneo. Journal of Ecology 95:828-839. |
| 0212 | Brooke, J. M., P. S. Basinger, J. L. Birckhead, M. A. Lashley, J. M. McCord, J. S. Nanney, and C. A. Harper. 2019. Effects of fertilization and crown release on white oak (Quercus alba) masting and acorn quality. Forest Ecology and Management 433:305-312. |
| 0213 | Brookes, R. H., and L. K. Jesson. 2007. No evidence for simultaneous pollen and resource limitation in Aciphylla squarrosa: A long-lived, masting herb. Austral Ecology 32:370-377. |
| 0219 | Brown, K., D. Zobel, and J. Zasada. 1988. Seed dispersal, seedling emergence, and early survival of Larix laricina (DuRoi) K. Koch in the Tanana Valley, Alaska. Canadian Journal of Forest Research 18:306-314. |
| 0223 | Brugger, K., M. Walter, L. Chitimia-Dobler, G. Dobler, and F. Rubel. 2018. Forecasting next season’s Ixodes ricinus nymphal density: the example of southern Germany 2018. Experimental and Applied Acarology:1-8. |
| 0225 | Bruinderink, G., and E. Hazebroek. 1995. Modelling carrying capacity for wild boar Sus scrofa scrofa in a forest/heathland ecosystem. Wildlife Biology 1:81-87. |
| 0234 | Buechling, A., P. H. Martin, C. D. Canham, W. D. Shepperd, and M. A. Battaglia. 2016. Climate drivers of seed production in Picea engelmannii and response to warming temperatures in the southern Rocky Mountains. Journal of Ecology 104:1051-1062. |
| 0251 | Caignard, T., S. Delzon, C. Bodénès, B. Dencausse, and A. Kremer. 2019. Heritability and genetic architecture of reproduction-related traits in a temperate oak species. Tree genetics & genomes 15:1. |
| 0253 | Calama, R., M. Fortin, M. Pardos, and R. Manso. 2017. Modelling spatiotemporal dynamics of Pinus pinea cone infestation by Dioryctria mendacella. Forest Ecology and Management 389:136-148. |
| 0254 | Calama, R., R. Manso, I. Barbeito, G. Madrigal, E. Garriga, F. J. Gordo, G. Montero, I. Cañellas, and M. Pardos. 2015. Do inter-specific differences in seed size determine natural regeneration traits in pinus pinea and pinus sylvestris? Applied Ecology and Environmental Research 13:387-404. |
| 0266 | Campbell, A. 1981. Flowering records for Chionochloa, Aciphylla, and Celmisia species in the Craigieburn Range, South Island, New Zealand. New Zealand journal of botany 19:97-103. |
| 0268 | Cañadas-López, Á., D. Rade-Loor, J. M. Domínguez-Andrade, J. J. Vargas-Hernández, C. Molina-Hidrovo, C. Macías-Loor, and C. Wehenkel. 2017. Variation in seed production of Jatropha curcas L. accessions under tropical dry forest conditions in Ecuador. New Forests 48:785-799. |
| 0273 | Cao, Y., Y. Xiao, H. Huang, J. Xu, W. Hu, and N. Wang. 2016. Simulated warming shifts the flowering phenology and sexual reproduction of Cardamine hirsuta under different planting densities. Scientific reports 6:27835. |
| 0275 | Cao, Y., Y. Xiao, H. Huang, J. Xu, W. Hu, and N. Wang. 2016. Simulated warming shifts the flowering phenology and sexual reproduction of Cardamine hirsuta under different planting densities. Scientific reports 6:27835. |
| 0285 | Caritat, A., E. Garcia-Berthou, R. Lapena, and L. Vilar. 2006. Litter production in a Quercus suber forest of Montseny (NE Spain) and its relationship to meteorological conditions. Annals of Forest Science 63:791-800. |
| 0300 | Cestari, C., and C. J. Bernardi. 2011. Predation of the Buffy-fronted seedeater Sporophila frontalis (Aves: Emberizidae) on Merostachys neesii (Poaceae: Babusoideae) seeds during a masting event in the atlantic forest. Biota Neotropica 11:407-411. |
| 0317 | CHAROENSUK, A., M. JAROENSUTASINEE, and K. JAROENSUTASINEE. 2017. Seed Production and Seedling Establishment of Parah Trees in Khao Nan National Park, Thailand. Walailak Journal of Science and Technology (WJST) 15:213-223. |
| 0338 | Chidumayo, E. N. 1997. Fruit production and seed predation in two miombo woodland trees in Zambia. Biotropica 29:452-458. |
| 0345 | Chong, K. Y., R. Chong, L. W. A. Tan, A. T. K. Yee, M. A. H. Chua, K. M. Wong, and H. T. W. Tan. 2016. Seed production and survival of four dipterocarp species in degraded forests in Singapore. Plant Ecology and Diversity 9:483-490. |
| 0347 | Choquenot, D., and W. A. Ruscoe. 2000. Mouse population eruptions in New Zealand forests: the role of population density and seedfall. Journal of Animal Ecology 69:1058-1070. |
| 0348 | Chrisman, A. B. 1980. Retrieving data. Wait a few seconds and try to cut or copy again. |
| 0352 | Christie, J. E., P. R. Wilson, R. H. Taylor, and G. Elliott. 2017. How elevation affects ship rat (Rattus rattus) capture patterns, Mt Misery, New Zealand. New Zealand Journal of Ecology 41:113-119. |
| 0358 | Clair, S. B. S., and J. Hoines. 2018. Reproductive ecology and stand structure of Joshua tree forests across climate gradients of the Mojave Desert. PloS one 13:e0193248. |
| 0362 | Cleavitt, N. L., and T. J. Fahey. 2017. Seed production of sugar maple and American beech in northern hardwood forests, New Hampshire, USA. Canadian Journal of Forest Research 47:985-990. |
| 0364 | Clevenger, A. P., F. J. Purroy, and M. R. Pelton. 1992. FOOD-HABITS OF BROWN BEARS (URSUS-ARCTOS) IN THE CANTABRIAN MOUNTAINS, SPAIN. Journal of Mammalogy 73:415-421. |
| 0366 | Clink, D. J., C. Dillis, K. L. Feilen, L. Beaudrot, and A. J. Marshall. 2017. Dietary diversity, feeding selectivity, and responses to fruit scarcity of two sympatric Bornean primates (Hylobates albibarbis and Presbytis rubicunda rubida). Plos One 12:23. |
| 0369 | Cockrem, J. F. 2006. The timing of breeding in the kakapo (Strigops habroptilus). Notornis 53:153-159. |
| 0378 | Conrod, C. A., and L. Reitsma. 2015. Demographic Responses of Myomorph Rodents to Mast Production in a Beech- and Birch-dominated Northern Hardwood Forest. Northeastern Naturalist 22:746-761. |
| 0413 | Cunze, S., J. Kochmann, T. Kuhn, R. Frank, D. D. Dorge, and S. Klimpel. 2018. Spatial and temporal patterns of human Puumala virus (PUUV) infections in Germany. Peerj 6:20. |
| 0417 | Curran, L. M., I. Caniago, G. D. Paoli, D. Astianti, M. Kusneti, M. Leighton, C. E. Nirarita, and H. Haeruman. 1999. Impact of El Nino and logging on canopy tree recruitment in Borneo. Science 286:2184-2188. |
| 0424 | Dąbrowska-Zapart, K., K. Chłopek, and T. Niedźwiedź. 2018. The impact of meteorological conditions on the concentration of alder pollen in Sosnowiec (Poland) in the years 1997–2017. Aerobiologia 34:469-485. |
| 0432 | Dalke, P. D. 1953. YIELDS OF SEEDS AND MAST IN 2ND GROWTH HARDWOOD FOREST, SOUTHCENTRALMISSOURI. Journal of Wildlife Management 17:378-380. |
| 0445 | de Andrés, E. G., J. J. Camarero, I. Martínez, and L. Coll. 2014. Uncoupled spatiotemporal patterns of seed dispersal and regeneration in Pyrenean silver fir populations. Forest Ecology and Management 319:18-28. |
| 0467 | DePrenger-Levin, M. E., J. M. R. Neale, T. A. Grant, C. Dawson, and Y. E. Baytok. 2013. Life History and Demography of Astragalus microcymbus Barneby (Fabaceae). Natural areas journal 33:264-276. |
| 0486 | Dimitri, L. A., W. S. Longland, K. C. Tonkel, B. G. Rector, and V. S. Kirchoff. 2018. Impacts of granivorous and frugivorous arthropods on pre-dispersal seed production of western juniper (Juniperus occidentalis). Arthropod-Plant Interactions 12:465-476. |
| 0488 | Din, H. H. M., N. B. Bakiri, R. S. Sukri, and F. H. Metali. 2018. Assessment Of Seedling Abundance, Survival And Growth Of Two Dipterocarp Species In Peat Swamp Forests Of Brunei Darussalam. BIOTROPIA-The Southeast Asian Journal of Tropical Biology 25:148-154. |
| 0492 | Dixon, A., and J. P. Haffield. 2013. Seed availability and timing of breeding of Common Crossbills Loxia curvirostra at Sitka Spruce Picea sitchensis dominated forestry plantations. Ardea 101:33-39. |
| 0510 | Du, X., Q. Guo, X. Gao, and K. Ma. 2007. Seed rain, soil seed bank, seed loss and regeneration of Castanopsis fargesii (Fagaceae) in a subtropical evergreen broad-leaved forest. Forest Ecology and Management 238:212-219. |
| 0511 | Du, Y. J., S. A. Queenborough, L. Chen, Y. Q. Wang, X. C. Mi, K. P. Ma, and L. S. Comita. 2017. Intraspecific and phylogenetic density-dependent seedling recruitment in a subtropical evergreen forest. Oecologia 184:193-203. |
| 0566 | Fedriani, J. M., T. Wiegand, G. Calvo, A. Suárez‐Esteban, M. Jácome, M. Żywiec, and M. Delibes. 2015. Unravelling conflicting density‐and distance‐dependent effects on plant reproduction using a spatially explicit approach. Journal of Ecology 103:1344-1353. |
| 0584 | Fitzgerald, B. M., M. G. Efford, and B. J. Karl. 2004. Breeding of house mice and the mast seeding of southern beeches in the Orongorongo Valley, New Zealand. New Zealand Journal of Zoology 31:167-184. |
| 0591 | Flo, V., J. Bosch, X. Arnan, C. Primante, A. M. M. González, H. Barril-Graells, and A. Rodrigo. 2018. Yearly fluctuations of flower landscape in a Mediterranean scrubland: Consequences for floral resource availability. PloS one 13:e0191268. |
| 0610 | Fredriksson, G. M., S. A. Wich, and Trisno. 2006. Frugivory in sun bears (Helarctos malayanus) is linked to El Nino related fluctuations in fruiting phenology, East Kalimantan, Indonesia. Biological Journal of the Linnean Society 89:489-508. |
| 0621 | Fujiki, D. 2018. Can frequent occurrence of Asiatic black bears around residential areas be predicted by a model-based mast production in multiple Fagaceae species? Journal of Forest Research 23:260-269. |
| 0626 | Fukumoto, H., and H. Kajimura. 2011. Effects of asynchronous acorn production by co-occurring Quercus trees on resource utilization by acorn-feeding insects. Journal of Forest Research 16:62-67. |
| 0638 | Gao, S., J. Wang, Z. Zhang, G. Dong, and J. Guo. 2012. Seed production, mass, germinability, and subsequent seedling growth responses to parental warming environment in Leymus chinensis. Crop and Pasture Science 63:87-94. |
| 0655 | Gartner, B., F. Chapin Iii, and G. Shaver. 1986. Reproduction of Eriophorum vaginatum by seed in Alaskan tussock tundra. The Journal of Ecology:1-18. |
| 0703 | Govindan, B. N., and R. K. Swihart. 2015. Community structure of acorn weevils (Curculio): inferences from multispecies occupancy models. Canadian Journal of Zoology 93:31-39. |
| 0706 | Graignic, N., F. Tremblay, and Y. Bergeron. 2014. Geographical variation in reproductive capacity of sugar maple (Acer saccharum Marshall) northern peripheral populations. Journal of Biogeography 41:145-157. |
| 0714 | Greenberg, C. H., and S. J. Zarnoch. 2018. A test of the predator satiation hypothesis, acorn predator size, and acorn preference. Canadian Journal of Forest Research 48:237-245. |
| 0719 | C. H. Greenberg, D. J. Levey and D. L. Loftis. 2007. Fruit production in mature and recently regenerated forests of the Appalachians. Journal of Wildlife Management, 71, 321-335 |
| 0747 | P. HAASE. 1986. Flowering records of some subalpine trees and shrubs at Arthur's Pass, New Zealand. New Zealand journal of ecology |
| 0752 | Hanley, M. E., B. I. Cook, and M. Fenner. 2018. Climate variation, reproductive frequency and acorn yield in English Oaks. Journal of Plant Ecology. |
| 0758 | Hamann, A. 2004. Flowering and fruiting phenology of a Philippine submontane rain forest: climatic factors as proximate and ultimate causes. Journal of Ecology 92:24-31. |
| 0780 | Harper, G. A. 2005. Heavy rimu (Dacrydium cupressinum) mast seeding and rat (Rattus spp.) population eruptions on Stewart Island/Rakiura. New Zealand Journal of Zoology 32:155-162. |
| 0789 | Hashimoto, Y., M. Kaji, H. Sawada, and S. Takatsuki. 2003. Five-year study on the autumn food habits of the Asiatic black bear in relation to nut production. Ecological Research 18:485-492. |
| 0797 | Haymes, K. L., and G. A. Fox. 2012. Variation among individuals in cone production in Pinus palustris (Pinaceae). American Journal of Botany 99:640-645. |
| 0805 | Heinrich, B. 2014. American chestnut seed dispersal and regeneration. Northeastern Naturalist 21:619-630. |
| 0806 | Heinrichs, S., H. Dierschke, T. Kompa, and W. Schmidt. 2018. Effect of phenology, nutrient availability and windthrow on flowering of Allium ursinum - results from long-term monitoring and experiments. Tuexenia:111-134. |
| 0807 | excelsa Bonpl., Lecythidaceae) em florestas nativas de Roraima. Revista Árvore. Viçosa, MG. 38: 133-144 |
| 0813 | Hernandez-Pedrero, R., and T. Valverde. 2017. The use of periodic matrices to model the population dynamics of the long-lived semelparous Furcraea parmentieri (Asparagaceae) in a temperate forest in central Mexico. Population Ecology 59:3-16. |
| 0815 | Herrera, C. M., P. Jordano, L. Lopezsoria, and J. A. Amat. 1994. RECRUITMENT OF A MAST-FRUITING, BIRD-DISPERSED TREE - BRIDGING FRUGIVORE ACTIVITY AND SEEDLING ESTABLISHMENT. Ecological Monographs 64:315-344. |
| 0818 | Herrero, J., I. Irizar, N. A. Laskurain, A. Garcia-Serrano, and R. Garcia-Gonzalez. 2005. Fruits and roots: wild boar foods during the cold season in the southwestern Pyrenees. Italian Journal of Zoology 72:49-52. |
| 0825 | Higaki, M. 2016. Prolonged diapause and seed predation by the acorn weevil, Curculio robustus, in relation to masting of the deciduous oak Quercus acutissima. Entomologia Experimentalis et Applicata 159:338-346. |
| 0826 | Hilasvuori, E., P. Hari, T. Aakala, E. Pulliainen, and J. Grace. 2014. changes in nitrogen content and isotopic composition in subarctic Empetrum nigrum seeds in the period 1976–2010. |
| 0830 | Hirayama, D., T. Fujii, S. Nanami, A. Itoh, and T. Yamakura. 2012. Two-year cycles of synchronous acorn and leaf production in biennial-fruiting evergreen oaks of subgenus Cyclobalanopsis (Quercus, Fagaceae). Ecological Research 27:1059-1068. |
| 0831 | Hirayama, D., S. Nanami, A. Itoh, and T. Yamakura. 2008. Individual resource allocation to vegetative growth and reproduction in subgenus Cyclobalanopsis (Quercus, Fagaceae) trees. Ecological Research 23:451-458. |
| 0847 | Homsher, R. B. 2012. Growth and Reproduction of Oaks in Southeastern Ohio. Ohio University. |
| 0855 | Hoshino, Y., H. Yonenobu, K. Yasue, Y. Nobori, and T. Mitsutani. 2008. On the radial-growth variations of Japanese beech (Fagus crenata) on the northernmost part of Honshu Island, Japan. Journal of Wood Science 54:183-188. |
| 0858 | Hossain, S. M. Y., J. P. Caspersen, and S. C. Thomas. 2017. Reproductive costs in Acer saccharum: exploring size-dependent relations between seed production and branch extension. Trees-Structure and Function 31:1179-1188. |
| 0866 | Ickes, K. 2001. Hyper-abundance of native wild pigs (Sus scrofa) in a lowland dipterocarp rain forest of peninsular Malaysia. Biotropica 33:682-690. |
| 0870 | Hudson, I. L., S. W. Kim, and M. R. Keatley. 2010. Climatic influences on the flowering phenology of four Eucalypts: a GAMLSS approach. Pages 209-228 Phenological research. Springer. |
| 0881 | Ichie, T., S. Igarashi, S. Yoshida, T. Kenzo, T. Masaki, and I. Tayasu. 2013. Are stored carbohydrates necessary for seed production in temperate deciduous trees? Journal of Ecology 101:525-531. |
| 0891 | Lio, A. 2013. Masting changes canopy structure, light interception, and photosynthesis in Fagus crenata |
| 0893 | Iku, A., T. Itioka, K. Kishimoto-Yamada, U. Shimizu-kaya, F. B. Mohammad, M. Y. Hossman, A. Bunyok, M. Y. A. Rahman, S. Sakai, and P. Meleng. 2017. Increased seed predation in the second fruiting event during an exceptionally long period of community-level masting in Borneo. Ecological Research 32:537-545. |
| 0901 | Ishihara, M. I., and K. Kikuzawa. 2009. Annual and spatial variation in shoot demography associated with masting in Betula grossa: comparison between mature trees and saplings. Annals of Botany 104:1195-1205. |
| 0913 | Izquierdo, L. P., and F. Pulido. 2013. Spatiotemporal variation in acorn production and damage in a Spanish holm oak (Quercus ilex) dehesa. Forest Systems 22:106-113. |
| 0941 | Joshi, B., and A. Tewari. 2009. Irregularity in frequency of mast seed years in Quercus floribunda a late successional species of Central Himalaya. Russian Journal of Ecology 40:482-485. |
| 0943 | Joubert, D. F., G. N. Smit, and M. T. Hoffman. 2013. The influence of rainfall, competition and predation on seed production, germination and establishment of an encroaching Acacia in an arid Namibian savanna. Journal of Arid Environments 91:7-13. |
| 0944 | In D. Greenland, D. Goodin, & R. Smith (Eds.), Climate variability and ecosystem response at long term ecological research (LTER) sites (pp. |
| 0952 | Kager, T., and J. Fietz. 2009. Food availability in spring influences reproductive output in the seed-preying edible dormouse (Glis glis). Canadian Journal of Zoology 87:555-565. |
| 0965 | Kanamori, T., N. Kuze, H. Bernard, T. P. Malim, and S. Kohshima. 2017. Fluctuations of population density in Bornean orangutans (Pongo pygmaeus morio) related to fruit availability in the Danum Valley, Sabah, Malaysia: a 10-year record including two mast fruitings and three other peak fruitings. Primates 58:225-235. |
| 0970 | Kashian, D. M. 2016. Sprouting and seed production may promote persistence of green ash in the presence of the emerald ash borer. Ecosphere 7:15. |
| 0977 | Kearvell, J. C., J. R. Young, and A. D. Grant. 2002. Comparative ecology of sympatric orange-fronted parakeets (Cyanoramphus malherbi) and yellow-crowned parakeets (C auriceps), South Island, New Zealand. New Zealand Journal of Ecology 26:139-148. |
| 0982 | Kellner, K. F., J. K. Riegel, N. I. Lichti, and R. K. Swihart. 2013a. Oak mast production and animal impacts on acorn survival in the Central Hardwoods. The Hardwood Ecosystem Experiment: a framework for studying responses to forest management Edited by RK Swihart, MR Saunders, RA Kalb, S. Haulton, and CH Michler. US Dep. Agric. For. Serv. Gen. Tech. Rep. No. NRSP-108:176-190. |
| 0983 | Kellner, K. F., N. A. Urban, and R. K. Swihart. 2013b. Short-Term Responses of Small Mammals to Timber Harvest in the United States Central Hardwood Forest Region. Journal of Wildlife Management 77:1650-1663. |
| 1010 | Khanduri, V. P. 2014. Annual variation in floral phenology and pollen production in Lagerstroemia speciosa: an entomophilous tropical tree. Songklanakarin Journal of Science & Technology 36. |
| 1020 | Jonczak, J. 2011. Structure, dynamics and properties of litterfall in a 110-year-old beech stand with admixture of pine and spruce. Sylwan 155:760-768. |
| 1022 | Khuu, B. 2017. The Effects of Climate on Singleleaf Pinyon Pine Cone Production across an Elevational Gradient. |
| 1086 | Kon, H., T. Noda, K. Terazawa, H. Koyama, and M. Yasaka. 2005. Proximate factors causing mast seeding in Fagus crenata: the effects of resource level and weather cues. Canadian Journal of Botany-Revue Canadienne De Botanique 83:1402-1409. |
| 1087 | Kon, H., and H. Saito. 2015. Test of the temperature difference model predicting masting behavior. Canadian Journal of Forest Research 45:1835-1844. |
| 1107 | Kroiss, S. J., J. Hillerislambers, and A. W. D'Amato. 2015. Recruitment limitation of long-lived conifers: Implications for climate change responses. Ecology 96:1286-1297. |
| 1120 | Kurten, E. L., S. Bunyavejchewin, and S. J. Davies. 2018. Phenology of a dipterocarp forest with seasonal drought: Insights into the origin of general flowering. Journal of Ecology 106:126-136. |
| 1139 | Langer, F., N. Havenstein, and J. Fietz. 2018. Flexibility is the key: metabolic and thermoregulatory behaviour in a small endotherm. Journal of Comparative Physiology B 188:553-563. |
| 1147 | Lavoie, C., K. Marcoux, A. Saint-Louis, and J. S. Price. 2005. The dynamics of a cotton-grass (Eriophorum vaginatum L.) cover expansion in a vacuum-mined peatland, southern Québec, Canada. Wetlands 25:64. |
| 1166 | Lehman, C. P., M. A. Rumble, and L. D. Flake. 2007. Winter habitat selection patterns of Merriam's turkeys in the southern Black Hills, South Dakota. Western North American Naturalist 67:278-291. |
| 1183 | Liu, W. Y., J. E. D. Fox, and Z. F. Xu. 2003. Litterfall and nutrient dynamics in a montane moist evergreen broad-leaved forest in Ailao Mountains, SW China. Plant Ecology 164:157-170. |
| 1185 | Rogers, M. J., L. K. Halls, and J. G. Dickson. 1990. DEER HABITAT IN THE OZARK FORESTS OF ARKANSAS. Usda Forest Service Southern Forest Experiment Station Research Paper:1-&. |
| 1212 | Liu, W. Y., J. E. D. Fox, and Z. F. Xu. 2003. Litterfall and nutrient dynamics in a montane moist evergreen broad-leaved forest in Ailao Mountains, SW China. Plant Ecology 164:157-170. |
| 1234 | Lovett, G. M., M. A. Arthur, K. C. Weathers, R. D. Fitzhugh, and P. H. Templer. 2013. Nitrogen Addition Increases Carbon Storage in Soils, But Not in Trees, in an Eastern US Deciduous Forest. Ecosystems 16:980-1001. |
| 1236 | Lowry, W. P. 1966. Apparent meteorological requirements for abundant cone crop in Douglas-fir. Forest Science 12:185-192. |
| 1242 | Lucas-Borja, M. E., and G. Vacchiano. 2018. Interactions between climate, growth and seed production in Spanish black pine (Pinus nigra Arn. ssp salzmannii) forests in Cuenca Mountains (Spain). New Forests 49:399-414. |
| 1250 | Macmillan, J., and L. W. Aarssen. 2017. Recruitment Success for Mast Year Cohorts of Sugar Maple (Acer saccharum) over Three Decades of Heavy Deer Browsing. American Midland Naturalist 178:36-46. |
| 1285 | Martin, D., J. Vazquez-Pique, F. S. Carevic, M. Fernandez, and R. Alejano. 2015a. Trade-off between stem growth and acorn production in holm oak. Trees-Structure and Function 29:825-834. |
| 1287 | Martin, P., J. Wishart, G. McDougall, and R. Brennan. 2015b. Fruit production and polyphenol content of salal (Gaultheria shallon Pursh), a potential new fruit for northern maritime regions. Fruits 70:377-383. |
| 1294 | Martinez-Alonso, C., F. Valladares, J. J. Camarero, M. L. Arias, M. Serrano, and J. A. Rodriguez. 2007. The uncoupling of secondary growth, cone and litter production by intradecadal climatic variability in a mediterranean scots pine forest. Forest Ecology and Management 253:19-29. |
| 1298 | Martinik, A., M. Kneifl, J. Kadavy, and R. Knott. 2017. Effect of thinning on acorn production of old sprout-origin sessile oaks (Quercus petraea /Matt./ Liebl.). Austrian Journal of Forest Science 134:163-180. |
| 1314 | Mattson, W. J. 1978. The role of insects in the dynamics of cone production of red pine. Oecologia 33:327-349. |
| 1318 | Mazur, R., A. P. Klimley, and K. Folger. 2013. Implications of the variable availability of seasonal foods on the home ranges of black bears, Ursus americanus, in the Sierra Nevada of California. Animal Biotelemetry 1. |
| 1322 | McCallum, D. A. 1990. Variable cone crops, migration, and dynamics of a population of mountain chickadees (Parus gambeli). Pages 103-116 Population Biology of Passerine Birds. Springer. |
| 1338 | McNulty, S. A., and R. D. Masters. 2005. Changes to the Adirondack Forest: Implications of beech bark disease on forest structure and seed production. Usda Forest Service, Newtown Square. |
| 1363 | Messier, G. D., D. Garant, P. Bergeron, and D. Réale. 2012. Environmental conditions affect spatial genetic structures and dispersal patterns in a solitary rodent. Molecular Ecology 21:5363-5373. |
| 1370 | Mezquida, E. T., and J. M. Olano. 2013. What makes a good neighborhood? Interaction of spatial scale and fruit density in the predator satiation dynamics of a masting juniper tree. Oecologia 173:483-492. |
| 1386 | Minor, D. M., and R. K. Kobe. 2017. Masting synchrony in northern hardwood forests: super-producers govern population fruit production. Journal of Ecology 105:987-998. |
| 1393 | Miyazaki, Y., N. Ohnishi, H. Takafumi, and T. Hiura. 2009a. Genets of dwarf bamboo do not die after one flowering event: evidence from genetic structure and flowering pattern. Journal of plant research 122:523-528. |
| 1394 | Miyazaki, Y., T. Osawa, and Y. Waguchi. 2009b. Resource level as a proximate factor influencing fluctuations in male flower production in Cryptomeria japonica D. Don. Journal of Forest Research 14:358-364. |
| 1403 | Mokake, S. E., G. B. Chuyong, A. E. Egbe, P. T. Tabot, B. Jumbam, B. J. N. Biyon, and S. D. Dibong. 2018. Plant reproductive phenology following selective logging in a semideciduous tropical forest in the East Region of Cameroon. Journal of Applied Biosciences 128:12901-12919. |
| 1404 | Molau, U. 2010. Long-term impacts of observed and induced climate change on tussock tundra near its southern limit in northern Sweden. Plant Ecology & Diversity 3:29-34. |
| 1416 | Montesinos, D., P. García-Fayos, and M. Verdú. 2012. Masting uncoupling: Mast seeding does not follow all mast flowering episodes in a dioecious juniper tree. Oikos 121:1725-1736. |
| 1429 | Moreira, X., L. Abdala-Roberts, R. Zas, E. Merlo, M. J. Lombardero, L. Sampedro, and K. A. Mooney. 2016. Masting behaviour in a Mediterranean pine tree alters seed predator selection on reproductive output. Plant Biology 18:973-980. |
| 1430 | Moreira, X., I. M. Pérez-Ramos, L. Abdala-Roberts, and K. A. Mooney. 2017. Functional responses of contrasting seed predator guilds to masting in two Mediterranean oak species. Oikos 126:1042-1050. |
| 1432 | D. Moreno Fernández. 2013. Effects of the first thinning on the growth and cone production of stone pine (Pinus pinea L.) stands in the Northern Plateau (Spain) |
| 1436 | Morgan, P., and S. C. Bunting. 1992. Using cone scars to estimate past cone crops of whitebark pine. Western Journal of Applied Forestry 7:71-73. |
| 1440 | Mori, T., R. Sugiura, M. Kato, H. Kato, and Y. Niizuma. 2018. A seven-year longitudinal study on the food habits of the Asiatic black bear (Ursus thibetanus) in relation to mast production in Shirakawa Village, Gifu Prefecture, Japan. Mammal Study 43:81-90. |
| 1448 | Moupela, C., J.-L. Doucet, K. Daïnou, Y. Brostaux, A. Fayolle, and C. Vermeulen. 2014. Reproductive ecology of Coula edulis Baill., source of a valuable nontimber forest product. Tropical Ecology 55. |
| 1450 | Moyer, M. A., J. W. McCown, and M. K. Oli. 2007. Factors influencing home-range size of female Florida black bears. Journal of Mammalogy 88:468-476. |
| 1458 | Muller-Haubold, H., D. Hertel, D. Seidel, F. Knutzen, and C. Leuschner. 2013. Climate Responses of Aboveground Productivity and Allocation in Fagus sylvatica: A Transect Study in Mature Forests. Ecosystems 16:1498-1516. |
| 1465 | Murphy, E. C. 1992. THE EFFECTS OF A NATURAL INCREASE IN FOOD-SUPPLY ON A WILD POPULATION OF HOUSE MICE. New Zealand Journal of Ecology 16:33-40. |
| 1470 | Murua, R., and M. Briones. 2005. Abundance of the sigmodont mouse Oligoryzomys longicaudatus and patterns of tree seeding in Chilean temperate forest. Mammalian Biology 70:321-326. |
| 1471 | Mutke, S., J. Gordo, and L. Gil. 2005a. Cone yield characterization of a stone pine (Pinus pinea L.) clone bank. Silvae Genetica 54:189-197. |
| 1472 | Mutke, S., J. Gordo, and L. Gil. 2005b. Variability of Mediterranean Stone pine cone production: Yield loss as response to climate change. Agricultural and Forest Meteorology 132:263-272. |
| 1486 | Nakagawa, M., M. Matsushita, H. Kurokawa, H. Samejima, Y. Takeuchi, M. Aiba, A. Katayama, Y. Tokumoto, T. Kume, N. Yoshifuji, K. Kuraji, H. Nagamasu, S. Sakai, and T. Nakashizuka. 2012. Possible Negative Effect of General Flowering on Tree Growth and Aboveground Biomass Increment in a Bornean Tropical Rain Forest. Biotropica 44:715-719. |
| 1492 | Nakajima, H. 2015. Estimating sound seedfall density of Fagus crenata using a visual survey. Journal of Forest Research 20:94-103. |
| 1495 | Nakamura, M., R. Hirata, K. Oishi, T. Arakaki, N. Takamatsu, K. Hata, and K. Sone. 2013. Determinant factors in the seedling establishment of Pasania edulis (Makino) Makino. Ecological Research 28:811-820. |
| 1499 | Nakashinden, I. 1995. Fruit years and form of cone production by the Japanese stone pine (Pinus Regel) estimated by the cone scars method. Japanese Journal of Ecology. |
| 1500 | Nangolo, E. M. 2016. Fruiting and seed production of producer and poor-producer baobab trees and on different land use types in Northern Venda, South Africa. |
| 1503 | Nault, A., and D. Gagnon. 1993. RAMET DEMOGRAPHY OF ALLIUM-TRICOCCUM, A SPRING EPHEMERAL, PERENNIAL FOREST HERB. Journal of Ecology 81:101-119. |
| 1516 | Nguyen, T. T., D. T. Tai, P. Zhang, M. Razaq, and H. L. Shen. 2019. Effect of thinning intensity on tree growth and temporal variation of seed and cone production in a Pinus koraiensis plantation. Journal of Forestry Research 30:835-845. |
| 1526 | Noguchi, S., Y. Kosugi, S. Takanashi, M. Tani, K. Niiyama, S. S. Aisah, and M. Lion. 2016. LONG-TERM VARIATION IN SOIL MOISTURE IN PASOH FOREST RESERVE, A LOWLAND TROPICAL RAINFOREST IN MALAYSIA. Journal of Tropical Forest Science:324-333. |
| 1560 | S. Oddou-Muratorio, C. Petit, V. Journe, M. Lingrand, J.-A. Magdalou, C. Hurson, J. Garrigue, H. Davi and E. Magnanou. 2018. Crown defoliation decreases reproduction and wood growth in a marginal European beech population. bioRxiv, 474874 |
| 1561 | O'Donnell, C. F. J., and J. M. Hoare. 2012a. Quantifying the benefits of long-term integrated pest control for forest bird populations in a New Zealand temperate rainforest. New Zealand Journal of Ecology 36:131-140. |
| 1562 | Odonnell, C. F. J., and S. M. Phillipson. 1996. Predicting the incidence of mohua predation from the seedfall, mouse, and predator fluctuations in beech forests. New Zealand Journal of Zoology 23:287-293. |
| 1563 | O'Donnell, C. F. J., and J. M. Hoare. 2012b. Quantifying the benefits of long-term integrated pest control for forest bird populations in a New Zealand temperate rainforest. New Zealand Journal of Ecology 36:131-140. |
| 1567 | Ogawa, R., A. Mortelliti, J. W. Witham, and M. L. Hunter. 2017. Demographic mechanisms linking tree seeds and rodent population fluctuations: insights from a 33-year study. Journal of Mammalogy 98:419-427. |
| 1573 | Oliva, G., M. Collantes, and G. Humano. 2013. Reproductive effort and seed establishment in grazed tussock grass populations of Patagonia. Rangeland ecology & management 66:164-173. |
| 1583 | Onodera, R., Y. Akimoto, T. Shimada, and T. Saitoh. 2017. Different population responses of three sympatric rodent species to acorn masting—the role of tannin tolerance. Population Ecology 59:29-43. |
| 1590 | Osumi, K., and S. Sakurai. 1997. Seedling emergence of Betula maximowicziana following human disturbance and the role of buried viable seeds. Forest Ecology and Management 93:235-243. |
| 1605 | Paluch, J. G. 2011. Ground seed density patterns under conditions of strongly overlapping seed shadows in Abies alba Mill. stands. European journal of forest research 130:1009-1022. |
| 1656 | Pérez-Ramos, I. M., Y. García-De La Cruz, and L. Gómez-Aparicio. 2017. Contrasting responses of insects and vertebrates as seed consumers of two neotropical oak species: The interactive effects of individual crop size and seed mass. Forest ecology and management 401:99-106. |
| 1674 | Peters, D. P., J. Yao, D. Browning, and A. Rango. 2014. Mechanisms of grass response in grasslands and shrublands during dry or wet periods. Oecologia 174:1323-1334. |
| 1698 | Pillay, R., F. Y. Hua, B. A. Loiselle, H. Bernard, and R. J. Fletcher. 2018. Multiple stages of tree seedling recruitment are altered in tropical forests degraded by selective logging. Ecology and Evolution 8:8231-8242. |
| 1708 | Pol, R. G., G. I. Pirk, and L. Marone. 2010. Grass seed production in the central Monte desert during successive wet and dry years. Plant Ecology 208:65-75. |
| 1736 | Purdey, D. C., C. M. King, and B. Lawrence. 2004. Age structure, dispersion and diet of a population of stoats (Mustela erminea) in southern Fiordland during the decline phase of the beech mast cycle. New Zealand Journal of Zoology 31:205-225. |
| 1753 | E. Ranta, J. Lindstrom, V. Kaitala, E. Crone, P. Lundberg, T. Hokkanen and E. Kubin. 2010. Life History Mediated Responses to Weather, Phenology and Large-Scale Population Patterns. Phenological Research: Methods for Environmental and Climate Change Analysis. 321-338 |
| 1754 | Ranta, H., and P. Satri. 2007. Synchronized inter-annual fluctuation of flowering intensity affects the exposure to allergenic tree pollen in North Europe. Grana 46:274-284. |
| 1755 | Rapp, J. M., and E. E. Crone. 2015. Maple syrup production declines following masting. Forest Ecology and Management 335:249-254. |
| 1759 | Ratiarison, S., and P. M. Forget. 2011. Fruit availability, frugivore satiation and seed removal in 2 primate-dispersed tree species. Integrative Zoology 6:178-194. |
| 1761 | Rayamajhi, M. B., P. D. Pratt, P. W. Tipping, T. D. Center, J. G. Leidi, and L. Rodgers. 2018. Natural-enemies affect the seed and litter fall dynamics of Melaleuca quinquenervia in the wetlands, and influence long-term species diversity in leaf-litter. Wetlands Ecology and Management:1-15. |
| 1787 | Riba‐Hernández, P., J. L. Segura, and J. Muñoz‐Valverde. 2016. Female fruit production depends on female flower production and crown size rather than male density in a continuous population of a tropical dioecious tree (Virola surinamensis). American Journal of Botany 103:1990-1999. |
| 1794 | Rive, A. C. 2010. Enhancing natural regeneration of white spruce (Picea glauca) via synchronization of a mast year with site manipulations in Abitibi, Quebec. Concordia University. |
| 1798 | Rocha, O. J., C. Gómez, J. L. Hamrick, D. W. Trapnell, P. E. Smouse, and G. Macaya. 2018. Reproductive consequences of variation in flowering phenology in the dry forest tree Enterolobium cyclocarpum in Guanacaste, Costa Rica. American journal of botany 105:2037-2050. |
| 1799 | Rocha, O. J., B. Vílchez, and A. L. Araya Anchetta. 2006. A mast fruiting episode of the tropical tree Peltogyne purpurea (Caesalpinaceae) in the Osa Peninsula, Costa Rica. Revista de Biologia Tropical 54:1151-1155. |
| 1813 | Rodriguez-Ramirez, E. C., T. Terrazas, and I. Luna-Vega. 2019. The influence of climate on the masting behavior of Mexican beech: growth rings and xylem anatomy. Trees-Structure and Function 33:23-35. |
| 1814 | Roeser, J. 1941. Some aspects of flower and cone production in ponderosa pine. Journal of Forestry 39:534-536. |
| 1822 | Rong, K., H. Yang, J. Z. Ma, C. Zong, and T. J. Cai. 2013. Food Availability and Animal Space Use Both Determine Cache Density of Eurasian Red Squirrels. Plos One 8:10. |
| 1856 | Calama, R., R. Manso, I. Barbeito, G. Madrigal, E. Garriga, F. J. Gordo, G. Montero, I. Cañellas, and M. Pardos. 2015. Do inter-specific differences in seed size determine natural regeneration traits in pinus pinea and pinus sylvestris? Applied Ecology and Environmental Research 13:387-404. |
| 1863 | Sala, A., K. Hopping, E. J. B. McIntire, S. Delzon, and E. E. Crone. 2012. Masting in whitebark pine (Pinus albicaulis) depletes stored nutrients. New Phytologist 196:189-199. |
| 1870 | Sanguinetti, J. 2014. Araucaria araucana (Molina) K. Koch seed production during 15 years at different populations in Lanín National Park (Neuquén-Argentina). Ecología Austral 24:265-275. |
| 1881 | Sarfati, M. 2008. Diapause by seed predators and parasitoids in Chionochloa mast seeding communities. |
| 1893 | Satake, A., Y. Y. Chen, C. Fletcher, and Y. Kosugi. 2019. Drought and cool temperature cue general flowering synergistically in the aseasonal tropical forests of Southeast Asia. Ecological Research. |
| 1897 | Sato, T., H. Tanouchi, and K. Takeshita. 1994. INITIAL REGENERATIVE PROCESSES OF DISTYLIUM-RACEMOSUM AND PERSEA-THUNBERGII IN AN EVERGREEN BROAD-LEAVED FOREST. Journal of Plant Research 107:331-337. |
| 1899 | Scarlett, T. L. 2004. Acorn production and winter reproduction in white-footed mice (Peromyscus leucopus) in a southern Piedmont forest. Southeastern Naturalist 3:483-494. |
| 1913 | Schnurr, J. L., R. S. Ostfeld, and C. D. Canham. 2002. Direct and indirect effects of masting on rodent populations and tree seed survival. Oikos 96:402-410. |
| 1917 | Schöning, C., X. Espadaler, I. Hensen, and F. Roces. 2004. Seed predation of the tussock-grass Stipa tenacissima L. by ants (Messor spp.) in south-eastern Spain: the adaptive value of trypanocarpy. Journal of Arid Environments 56:43-61. |
| 1918 | Schooley, H. O. 1978. Effects of spruce budworm on cone production by balsam fir. The Forestry Chronicle 54:298-301. |
| 1919 | Schooley, R. L., C. R. McLaughlin, G. J. Matula, and W. B. Krohn. 1994. DENNING CHRONOLOGY OF FEMALE BLACK BEARS - EFFECTS OF FOOD, WEATHER, AND REPRODUCTION. Journal of Mammalogy 75:466-477. |
| 1956 | Selonen, V., R. Wistbacka, and E. Korpimaki. 2016. Food abundance and weather modify reproduction of two arboreal squirrel species. Journal of Mammalogy 97:1376-1384. |
| 1986 | Shibata, E. 2007. Effects of deer debarking on masting and trunk radial growth of Hondo spruce, Picea jewensis var. hondoensis, in a subalpine forest of central Japan. Forest Ecology and Management 252:159-164. |
| 2000 | Simón, Latorre and Rotundo. 2018. Study of the reproductive phenology of Araucaria angustifolia in two environments of Argentina: Its application to the management of a species at risk. Global Ecology and Conservation, 16, e00483 |
| 2005 | Gao, X. M., and S. C. Sun. 2005. Effects of the small forest carnivores on the recruitment and survival of Liaodong oak (Quercus wutaishanica) seedlings. Forest Ecology and Management 206:283-292. |
| 2007 | Sirois, L. 2000. Spatiotemporal variation in black spruce cone and seed crops along a boreal forest-tree line transect. Canadian Journal of Forest Research 30:900-909. |
| 2022 | Snook, L. K., L. Cámara-Cabrales, and M. J. Kelty. 2005. Six years of fruit production by mahogany trees (Swietenia macrophylla King): Patterns of variation and implications for sustainability. Forest Ecology and Management 206:221-235. |
| 2026 | Soler, R., J. M. Espelta, M. V. Lencinas, P. L. Peri, and G. Martínez Pastur. 2017. Masting has different effects on seed predation by insects and birds in antarctic beech forests with no influence of forest management. Forest Ecology and Management 400:173-180. |
| 2034 | Souza, M. L., and M. Fagundes. 2017. Seed predation of Copaifera langsdorffii (F abaceae): a tropical tree with supra‐annual fruiting. Plant species biology 32:66-73. |
| 2037 | Spector, D. A. 1999. The influence of forest structure on cone production in whitebark pine throughout the Greater Yellowstone Ecosystem. Montana State University-Bozeman, College of Letters & Science. |
| 2056 | Stiell, W. 1988. Consistency of cone production in individual red pine. The Forestry Chronicle 64:480-484. |
| 2059 | Stone, Z. L., B. Burns, R. Moorhouse, and M. N. Clout. 2017. Kakapo habitat selection on Hauturu-O-Toi in relation to plant phenology. New Zealand Journal of Ecology 41. |
| 2065 | Straub, J. N., R. M. Kaminski, A. G. Leach, A. W. Ezell, and T. Leininger. 2015. Acorn yield and masting traits of red oaks in the lower mississippi river alluvial valley. Forest Science 62:18-27. |
| 2081 | Sullivan, T. P., D. S. Sullivan, R. Boonstra, C. J. Krebs, and A. Vyse. 2017. Mechanisms of population limitation in the southern red-backed vole in conifer forests of western North America: insights from a long-term study. Journal of Mammalogy 98:1367-1378. |
| 2096 | Sweeney, J., and D. T. Quiring. 1998. Oviposition site selection and intraspecific competition influence larval survival and pupal weight of Strobilomyia neanthracina (Diptera: Anthomyiidae) in white spruce. Ecoscience 5:454-462. |
| 2114 | Tamrakar, R., M. B. Rayment, F. Moyano, M. Mund, and A. Knohl. 2018. Implications of structural diversity for seasonal and annual carbon dioxide fluxes in two temperate deciduous forests. Agricultural and Forest Meteorology 263:465-476. |
| 2119 | Tang, W. 1990. Reproduction in the cycad Zamia pumila in a fire-climax habitat: an eight-year study. Bulletin of the Torrey Botanical Club:368-374. |
| 2132 | Taylor Jr, O. R., and D. W. Inouye. 1985. Synchrony and periodicity of flowering in Frasera speciosa (Gentianaceae). Ecology 66:521-527. |
| 2134 | Te Wong, S., C. Servheen, L. Ambu, and A. Norhayati. 2005. Impacts of fruit production cycles on Malayan sun bears and bearded pigs in lowland tropical forest of Sabah, Malaysian Borneo. Journal of Tropical Ecology 21:627-639. |
| 2155 | Tochigi, K., T. Masaki, A. Nakajima, K. Yamazaki, A. Inagaki, and S. Koike. 2018. Detection of arboreal feeding signs by Asiatic black bears: effects of hard mast production at individual tree and regional scales. Journal of Zoology 305:223-231. |
| 2159 | Tompkins, D. M., A. E. Byrom, and R. P. Pech. 2013. Predicted responses of invasive mammal communities to climate-related changes in mast frequency in forest ecosystems. Ecological Applications 23:1075-1085. |
| 2162 | Tormo-Molina, R., J.-M. Maya-Manzano, I. Silva-Palacios, S. Fernández-Rodríguez, and Á. Gonzalo-Garijo. 2015. Flower production and phenology in Dactylis glomerata. Aerobiologia 31:469-479. |
| 2163 | Tornberg, R., P. Helle, and E. Korpimäki. 2011. Vulnerability of black grouse hens to goshawk predation: Result of food supply or predation facilitation? Oecologia 166:577-584. |
| 2176 | Tseng, Y.-T., S. Kawashima, S. Kobayashi, S. Takeuchi, and K. Nakamura. 2018. Algorithm for forecasting the total amount of airborne birch pollen from meteorological conditions of previous years. Agricultural and Forest Meteorology 249:35-43. |
| 2190 | Tutin, C. E., and M. Fernandez. 1993. Relationships between minimum temperature and fruit production in some tropical forest trees in Gabon. Journal of Tropical Ecology 9:241-248. |
| 2214 | Van Mantgem, P. J., N. L. Stephenson, and J. E. Keeley. 2006. Forest reproduction along a climatic gradient in the Sierra Nevada, California. Forest Ecology and Management 225:391-399. |
| 2215 | Van Schaik, C. 1986. Phenological changes in a Sumatran rain forest. Journal of Tropical Ecology 2:327-347. |
| 2230 | Vargas, I. N., and P. R. Stevenson. 2013. Seed and establishment limitation: Effects on plant diversity in an Amazonian rain forest. Biotropica 45:737-746. |
| 2243 | Venturas, M., P. Fuentes-Utrilla, R. López, R. Perea, V. Fernández, A. Gascó, P. Guzmán, M. Li, J. Rodríguez-Calcerrada, E. Miranda, J. Domínguez, G. González-Gordaliza, E. Zafra, M. Fajardo-Alcántara, J. A. Martín, R. Ennos, N. Nanos, J. J. Lucena, S. Iglesias, C. Collada, and L. Gil. 2014. Ulmus laevis in the iberian peninsula: A review of its ecology and conservation. IForest 8:135-142. |
| 2248 | Verma, A., S. Shah, and A. Tewari. 2015. Survival problem in regeneration of high altitude kharsu oak (Qurecus semecarpifolia Smith.) forests in Central Himalaya. International Journal of Bioassays vol 4 (03):3689-3692. |
| 2249 | Vessey, S. H., and K. B. Vessey. 2007. Linking behavior, life history and food supply with the population dynamics of white-footed mice (Peromyscus leucopus). Integrative Zoology 2:123-130. |
| 2253 | Vilà-Cabrera, A., J. Martínez-Vilalta, and J. Retana. 2014. Variation in reproduction and growth in declining Scots pine populations. Perspectives in Plant Ecology, Evolution and Systematics 16:111-120. |
| 2279 | Wang, Y. Y., J. Zhang, J. M. LaMontagne, F. Lin, B. H. Li, J. Ye, Z. Q. Yuan, X. G. Wang, and Z. Q. Hao. 2017b. Variation and synchrony of tree species mast seeding in an old-growth temperate forest. Journal of Vegetation Science 28:413-423. |
| 2280 | Wells, K., and R. Bagchi. 2005. Eat in or take away - Seed predation and removal by rats (muridae) during a fruiting event in a dipterocarp rainforest. Raffles Bulletin of Zoology 53:281-286. |
| 2284 | Wang, J., B. Zhang, X. Hou, X. N. Chen, N. Han, and G. Chang. 2017a. Effects of mast seeding and rodent abundance on seed predation and dispersal of Quercus aliena (Fagaceae) in Qinling Mountains, Central China. Plant Ecology 218:855-865. |
| 2290 | Webster, C. R., M. A. Jenkins, and A. J. Poznanovic. 2015. Spatial patterning and floral synchrony among trillium populations with contrasting histories of herbivory. PeerJ 3:e782. |
| 2294 | Wells, K., and R. Bagchi. 2005. Eat in or take away - Seed predation and removal by rats (muridae) during a fruiting event in a dipterocarp rainforest. Raffles Bulletin of Zoology 53:281-286. |
| 2298 | Wesolowski, T., P. Rowinski, and M. Maziarz. 2015. Interannual variation in tree seed production in a primeval temperate forest: does masting prevail? European Journal of Forest Research 134:99-112. |
| 2306 | White, E. 1975. An investigation and survey of insect damage affecting Chionochloa seed production in some alpine tussock grasslands. New Zealand journal of agricultural research 18:163-178. |
| 2332 | Winarni, N. L., D. R. Kurniasari, D. Hartiningtias, M. Nusalawo, and N. Sakuntaladewi. 2016. Phenology, Climate, and Adaptation: How Does Dipterocarps Respond to Climate? Indonesian Journal of Forestry Research 3:129-141. |
| 2342 | Wood, C. M., J. W. Witham, and M. L. Hunter Jr. 2016. Climate‐driven range shifts are stochastic processes at a local level: two flying squirrel species in Maine. Ecosphere 7:e01240. |
| 2366 | Wrobel, A., and R. Zwolak. 2019. Habitat-dependent seed dispersal of an introduced tree species by native rodents. Forest Ecology and Management 433:563-568. |
| 2371 | Xiao, Z. S., Z. B. Zhang, and C. J. Krebs. 2013b. Long-term seed survival and dispersal dynamics in a rodent-dispersed tree: testing the predator satiation hypothesis and the predator dispersal hypothesis. Journal of Ecology 101:1256-1264. |
| 2373 | Xiao, Z., X. Gao, and Z. Zhang. 2013a. Sensitivity to Seed Germination Schedule by Scatter-Hoarding Pére David's Rock Squirrels During Mast and Non-Mast Years. Ethology 119:472-479. |
| 2385 | Yamaji, T., M. Aizawa, F. Komai, T. Ohoka, and T. Ohkubo. 2016. Masting of beech (Fagus crenata) results in satiation of a nut predator, the dominant micromoth, Pseudopammene fagivora, in a beech forest near the Pacific Ocean, Japan. Nihon Ringakkai Shi/Journal of the Japanese Forestry Society 98:26-30. |
| 2431 | Zalewski, A. 1996. Choice of age classes of bank voles Clethrionomys glareolus by pine marten Martes martes and tawny owl Strix aluco in Bialowieza National Park. Acta Oecologica-International Journal of Ecology 17:233-244. |
| 2443 | H. Zhang, Y. Wang and Z. Zhang. 2009. Domestic goat grazing disturbance enhances tree seed removal and caching by small rodents in a warm-temperate deciduous forest in China. Wildlife Research. 36, 610-616 |
| 2447 | Y. Zhang, A. W. Bartlow, Z. Wang and X. Yi. 2018. Effects of tannins on population dynamics of sympatric seed-eating rodents: the potential role of gut tannin-degrading bacteria. Oecologia. 187, 667-678. |
| 2462 | Zong, C., L. A. Wauters, S. Van Dongen, V. Mari, C. Romeo, A. Martinoli, D. Preatoni, and G. Tosi. 2010. Annual variation in predation and dispersal of Arolla pine (Pinus cembra L.) seeds by Eurasian red squirrels and other seed-eaters. Forest Ecology and Management 260:587-594. |
| 2477 | Zagt, R.J. 1997. Pre-dispersal and early post-dispersal demography, and reproductive litter production, in the subtropical tree Dicymbe altsonii in Guyana. Journal of Tropical Ecology, 13, 511-526 |
| 2492 | Henkel, T. W., and J. R. Mayor. 2019. Implications of a long-term mast seeding cycle for climatic entrainment, seedling establishment and persistent monodominance in a Neotropical, ectomycorrhizal canopy tree. Ecological Research 34:472-484. |
| 2494 | Clark, D.A., Clark, D.B. 1987. Temporal and Environmental Patterns of Reproduction in Zamia Skinneri, A Tropical Rain Forest Cycad. Journal of Ecology, 75, 135-149 |
| 2500 | Askeyev, O. V., D. Tischin, T. H. Sparks, and I. V. Askeyev. 2005. The effect of climate on the phenology, acorn crop and radial increment of pedunculate oak (Quercus robur) in the middle Volga region, Tatarstan, Russia. International Journal of Biometeorology 49:262-266. |
| 2501 | Camargo, M. G. G., R. M. Souza, P. Reys, and L. P. C. Morellato. 2011. Effects of environmental conditions associated to the cardinal orientation on the reproductive phenology of the cerrado savanna tree Xylopia aromatica (Annonaceae). Anais Da Academia Brasileira De Ciencias 83:1007-1019. |
| 2503 | Daskalakou, E. N., K. Koutsovoulou, K. Ioannidis, P. P. Koulelis, P. Ganatsas, and C. A. Thanos. 2019. Masting and regeneration dynamics of Abies cephalonica, the Greek endemic silver fir. Seed Science Research 29:227-237. |
| 2504 | Contreras Arribas, E., Prades Lopez, C., Martin de Almagro, R. 2009. Análisis del ciclo de producción de piña de Pinus pinea L. en la comarca de Villaviciosa de Córdoba y de la influencia de los factores climáticos en la variación interanual. 5º Congreso Forestal Español |
| 2505 | Carbonero, M.D., Fernández-Rebollo, P. 2014. Dehesas de encinas. Influencia de la meteorología en la producción de bellotas. Ecosistemas, 23, 55-63 |
| 2508 | Di Pierro, E., A. Ghisla, L. A. Wauters, A. Molinari, A. Martinoli, J. Gurnell, and G. Tosi. 2011. The effects of seed availability on habitat use by a specialist seed predator. European Journal of Wildlife Research 57:585-595. |
| 2509 | Eastham, A. M., and M. J. Jull. 1999. Factors affecting natural regeneration of Abies lasiocarpa and Picea engelmannii in a subalpine silvicultural systems trial. Canadian Journal of Forest Research-Revue Canadienne De Recherche Forestiere 29:1847-1855. |
| 2510 | Falls, J. B., E. A. Falls, and J. M. Fryxell. 2007. Fluctuations of deer mice in Ontario in relation to seed crops. Ecological Monographs 77:19-32. |
| 2511 | Klisz, M., R. Puchalka, S. Wilczynski, W. Kantorowicz, T. Jablonski, and J. Kowalczyk. 2019. The Effect of Insect Defoliations and Seed Production on the Dynamics of Radial Growth Synchrony among Scots Pine Pinus sylvestris L. Provenances. Forests 10:21. |
| 2512 | Klemola, T., S. Hanhimaki, K. Ruohomaki, J. Senn, M. Tanhuanpaa, P. Kaitaniemi, H. Ranta, and E. Haukioja. 2003. Performance of the cyclic autumnal moth, Epirrita autumnata, in relation to birch mast seeding. Oecologia 135:354-361. |
| 2513 | Crawley, M. J., and C. R. Long. 1995. ALTERNATE BEARING, PREDATOR SATIATION AND SEEDLING RECRUITMENT IN QUERRCUS-ROBUR L. Journal of Ecology 83:683-696. |
| 2514 | Čepelka, L., Šipoš, J., Suchomel, J., Heroldová, M. 2020. Can we detect response differences among dominant rodent species to climate and acorn crop in a Central European forest environment? European Journal of Forest Research. https://doi.org/10.1007/s10342-020-01267-7 |
| 2515 | Tissier, M. L., D. Reale, D. Garant, and P. Bergeron. 2020. Consumption of red maple in anticipation of beech mast-seeding drives reproduction in eastern chipmunks. Journal of Animal Ecology 89:1190-1201. |
| 2516 | Jeynes, B. 2018. Counting rimu fruit on kākāpō islands. Unpublished data, Deaprtment of Conservation Blog. https://blog.doc.govt.nz/2018/03/16/counting-rimu-fruit/ |
| 2519 | Polansky, L., and C. Boesch. 2013. Long‐term changes in fruit phenology in a West African lowland tropical rain forest are not explained by rainfall. Biotropica 45:434-440. |
| 2523 | Mendoza, I., R. S. Condit, S. J. Wright, A. Caubère, P. Châtelet, I. Hardy, and P. M. Forget. 2018. Inter-annual variability of fruit timing and quantity at Nouragues (French Guiana): insights from hierarchical Bayesian analyses. Biotropica 50:431-441. |
| 2526 | Sakio, H., Kubo, M. 2020. Coexistence of Tree Canopy Species. In: Sakio H. (eds) Long-Term Ecosystem Changes in Riparian Forests. Ecological Research Monographs. Springer, Singapore. https://link.springer.com/chapter/10.1007/978-981-15-3009-8_2 |
| 2529 | Sambo, O., O. Oumarou, T. Adjima, and B. I. Joseph. 2020. Fruit production in Balanites aegyptiaca is highly variable across tropical arid zones of West Africa: implications for sustainability. Journal of Horticultural Science & Biotechnology 95:211-221. |
| 2531 | McDonald, P. M. 1992. ESTIMATING SEED CROPS OF CONIFER AND HARDWOOD SPECIES. Canadian Journal of Forest Research-Revue Canadienne De Recherche Forestiere 22:832-838. |
| 2534 | Meserve, P.L., Kelt, D.A., Milstead, W.B., Gutierrez, J.R. 2003. Thirteen Years of Shifting Top-Down and Bottom-Up Control. BioScience, 53, 633-646 |
| 2535 | Murcia Nova, M.A. 2019. ESTRUCTURA POBLACIONAL Y PRODUCCIÓN DE FRUTOS DE LA PALMA Dictyocaryum lamarckianum COMO ESTRATEGIA DE CONSERVACIÓN DEL LORO OREJIAMARILLO Ognorhynchus icterotis. Unpublished PhD thesis |
| 2536 | Artega, L.L. 2005. Fenologia y produccion de semillas de especies arboreas maderables en un bosque humedo montano de Bolivia. Revista boliviana de ecología y conservación ambiental, 21, 57-68 |
| 2538 | Debandi, G., Rossi, B.E., Villagra, P.E., Giantomasi, M.A., Mantovan., N.G. 2538. Sincronización espacial y temporal de los eventos fenológicos de Prosopis flexuosa en el Desierto del Monte Central. Revista de la Facultad de Ciencias Agrarias de la Universidad Nacional de Cuyo |
| 2538 | Donoso, C., Hernandez, M., Navarro, C. 1993. Valores de producción de semillas y hojarasca de diferentes especies del tipo forestal siempreverde de la Cordillera de la Costa de Valdivia obtenidos durante un período de 10 años. Bosque, 14, 65-84 |
| 2539 | Donoso, C., Maureira, C., Zuniga, A., Castro, H. 1999. Producción de semillas y hojarasca en renovales de canelo (Drimys winteri Forst.) en la Cordillera de la Costa de Valdivia, Chile. Bosque, 20, 65-78 |
| 2540 | Murua, R., Gonzalez, L.A. 1985. PRODUCCION DE SEMILLAS DE ESPECIES ARBOREAS EN LA PLUVISELVA VALDIVIANA. Bosque, 6, 15-23 |
| 2541 | Donoso, C. 1993. Producción de semillas y hojarasca de las especies del tipo forestal alerce (Fitzroya cupressoides) de la Cordillera de la Costa de Valdivia, Chile. Revista Chilena de Historia Natural, 66, 53-64 |
| 2542 | Sakai, S., Itioka, T. 2016. Long-term monitoring of plant reproductive phenology and observation of general flowering in Lambir Hills, Sarawak. Proceedings of the symposium "Frontier in tropical forest research: progress in joint projects between the Forest Department Sarawak and the Japan Research Consortium for Tropical Forests in Sarawak". http://hdl.handle.net/2433/227104 |
| 2543 | Cools, N., Verstraeten A., Sioen G., Neirynck J., Roskams P., Louette G., Hoffmann M. 2016. LTER-Belgium - Results of long-term, large-scale and intensive monitoring at the Flemish forest condition monitoring sites within the LTER-Belgium network. Rapporten van het Instituut voor Natuur- en Bosonderzoek 2016 (INBO.R.2016.11433903). Instituut voor Natuur- en Bosonderzoek, Brussel |
| 2544 | Barrere, J., V. Boulanger, C. Collet, E. Walker, V. Siat, L. Henry, and S. Said. 2020. How does oak mast seeding affect the feeding behavior of sympatric red and roe deer? Basic and Applied Ecology 47:83-94. |
| 2545 | Zhang, H., C. Yan, S. Wu, J. Si, X. Yi, H. Li, and Z. Zhang. 2020. Effects of masting on seedling establishment of a rodent-dispersed tree species in a warm-temperate region, northern China. Integrative Zoology. |
| 2546 | Greenberg, C. H. 2021. Oak growth and acorn production in southern Appalachian mature forests and shelterwood with reserves regeneration harvests. Forest Ecology and Management 481:7. |
| 2547 | Jin, X. J., F. R. Li, T. Pukkala, and L. H. Dong. 2020. Modelling the cone yields of Korean pine. Forest Ecology and Management 464:9. |
| 2548 | Leung, C., B. Angers, and P. Bergeron. 2020. Epigenetic anticipation for food and reproduction. Environmental Epigenetics 6:8. |
| 2549 | Lazaro, A., A. Traveset, and M. Mendez. 2006. Masting in Buxus balearica: assessing fruiting patterns and processes at a large spatial scale. Oikos 115:229-240. |
| 2551 | Yang, X. F., C. Yan, H. F. Gu, and Z. B. Zhang. 2020. Interspecific synchrony of seed rain shapes rodent-mediated indirect seed-seed interactions of sympatric tree species in a subtropical forest. Ecology Letters 23:45-54. |
| 2552 | Šenfeldr, M., Treml, V. 2020. Which generative reproduction characteristics determine successful establishment of the subalpine shrub Pinus mugo? Journal of Vegetation Science, 31, 403-415 |
| 2553 | Wright, M. C., P. van Mantgem, N. L. Stephenson, A. J. Das, and J. E. Keeley. 2021. Seed production patterns of surviving Sierra Nevada conifers show minimal change following drought. Forest Ecology and Management 480:21. |
| 2554 | Masaki, T., S. Abe, S. Naoe, S. Koike, A. Nakajima, Y. Nemoto, and K. Yamazaki. 2020. Horizontal and elevational patterns of masting across multiple species in a steep montane landscape from the perspective of forest mammal management. Journal of Forest Research 25:92-100. |
| 2555 | Satake, A., T. L. Yao, Y. Kosugi, and Y. Y. Chen. 2021. Testing the environmental prediction hypothesis for community-wide mass flowering in South-East Asia. Biotropica 53:608-618. |
| 2556 | Law, B., C. Mackowski, L. Schoer, and T. Tweedie. 2000. Flowering phenology of myrtaceous trees and their relation to climatic, environmental and disturbance variables in northern New South Wales. Austral Ecology 25:160-178. |
| 2557 | Wion, A. P., P. J. Weisberg, I. S. Pearse, and M. D. Redmond. 2020. Aridity drives spatiotemporal patterns of masting across the latitudinal range of a dryland conifer. Ecography 43:569-580. |
| 2558 | Bouchard, M., and C. Pernot. 2021. Climate and size of previous cone crops contribute to large-scale synchronous cone production in balsam fir. Canadian Journal of Forest Research 51:638-646. |
| 2559 | Smith, S.J. 2019. Population-level and Individual-level Drivers of Reproduction in Chestnut Oak (Quercus montana Willd.) and Black Oak (Q. velutina Lam.) in Southeast Ohio. http://rave.ohiolink.edu/etdc/view?acc_num=ohiou1565785479095924 |
| 2560 | Le Ronce, I., Gavinet, J., Ourcival, J-M., Mouillot, F., Chuine, I., Limousin, J-M. 2021. Holm oak fecundity does not acclimate to a drier world. New Phytologist. 10.1111/nph.17412 |
| 2561 | Staudhammer, C.L., Wadt, L.H.O., Kainer, K.A., da Cunha, T.A. 2021. Comparative models disentangle drivers of fruit production variability of an economically and ecologically important long‑lived Amazonian tree. Scientific Reports. 10.1038/s41598-021-81948-4 |
| 2562 | Flowerdew, J. R., T. Amano, and W. J. Sutherland. 2017. Strong "bottom-up" influences on small mammal populations: State-space model analyses from long-term studies. Ecology and Evolution 7:1699-1711. |
| 2563 | Sütő, Siffer, Farkas, Katona. 2021. Problems related to oak regeneration in Central Europe: from acorn production to wild boar rooting. Unpublished poster |
| 2564 | Krebs, C. J., R. Boonstra, K. Cowcill, and A. J. Kenney. 2009. Climatic determinants of berry crops in the boreal forest of the southwestern Yukon. Botany-Botanique 87:401-408. |
| 2565 | Furness, E. N., and R. W. Furness. 2021. Effects of Sitka spruce masting on phenology and demography of siskins Spinus spinus. Scientific Reports 11. |
| 2566 | Shaw, G. 1990. TIMING AND FIDELITY OF BREEDING FOR SISKINS CARDUELIS-SPINUS IN SCOTTISH CONIFER PLANTATIONS. Bird Study 37:30-35. |
| 2567 | Petty, S. J., I. J. Patterson, D. I. K. Anderson, B. Little, and M. Davison. 1995. Numbers, breeding performance, and diet of the sparrowhawk Accipiter nisus and merlin Falco columbarius in relation to cone crops and seed-eating finches. Forest Ecology and Management 79:133-146. |
| 2568 | Doublet, V., C. Gidoin, F. Lefevre, and T. Boivin. 2019. Spatial and temporal patterns of a pulsed resource dynamically drive the distribution of specialist herbivores. Scientific Reports 9. |
| 2569 | Gamelon, M., L. Touzot, E. Baubet, J. Cachelou, S. Focardi, B. Franzetti, E. Nivois, L. Veylit, and B. E. Saether. 2021. Effects of pulsed resources on the dynamics of seed consumer populations: a comparative demographic study in wild boar. Ecosphere 12:19. |
| 2570 | Wang, J., B. Zhang, N. Han, T. Feng, X. Hou, X. L. An, X. N. Chen, and G. Chang. 2021. "Effects of mast seeding and insect infestation on predation and dispersal of Castanea mollissima nuts by rodents in the Qinling Mountains of China." Forest Ecology and Management 499:8. doi: 10.1016/j.foreco.2021.119630. |
| 2571 | Wright, B. R. "Evidence that predator satiation drives reproductive synchrony in the desert masting grass, soft spinifex (Triodia pungens)." Austral Ecology:12. doi: 10.1111/aec.13119. |
| 2572 | Pastana, D. N. B., E. D. S. Modena, L. H. D. Wadt, E. D. S. Neves, L. G. Martorano, A. C. Lira-Guedes, R. L. F. de Souza, F. F. Costa, A. P. B. Batista, and M. C. Guedes. 2021. "Strong El Nino reduces fruit production of Brazil-nut trees in the eastern Amazon." Acta Amazonica 51 (3):270-+. doi: 10.1590/1809-4392202003702. |
| 2573 | Szwagrzyk, J., G. Gratzer, H. Stepniewska, J. Szewczyk, and B. Veselinovic. 2015. "High reproductive effort and low recruitment rates of European beech: Is there a limit for the superior competitor?" Polish Journal of Ecology 63 (2):198-212. doi: 10.3161/15052249pje2015.63.2.004. |
| 2574 | Tonini, H., A.B. Baldoni, and S. de Carvalho Campos Botelho. 2020. "DIAMETER STRUCTURE AND ITS RELATIONSHIP WITH FRUIT AND SEED PRODUCTION IN A NATIVE BRAZIL NUT GROVE IN MATO GROSSO." FLORESTA 50:1399-1410. |
| 2575 | Hirayama, K., T. Imai, K. Enomoto, and C. Tachikawa. 2017. "Annual variability in acorn production and pre-dispersal damage to acorns of four fagaceous species in two adjacent forest stands with different mixed ratios in western Japan." Population Ecology 59 (4):343-354. doi: 10.1007/s10144-017-0595-0. |
| 2576 | Ida, H. 2021. "A 15-year study on the relationship between beech (Fagus crenata) reproductive-organ production and the numbers of nuisance Japanese black bears (Ursus thibetanus japonicus) killed in a snowy rural region in central Japan." Landscape and Ecological Engineering 17 (4):507-514. doi: 10.1007/s11355-021-00472-9. |
| 2577 | Goroshkevich, S., S. Velisevich, A. Popov, O. Khutornoy, and G. Vasilyeva. 2021. "30-year cone production dynamics in Siberian stone pine (Pinus sibirica) in the southern boreal zone: a causal interpretation." Plant Ecology and Evolution 154:321-331. doi: 10.5091/plecevo.2021.1793. |
| 2578 | Huang, L., C. Jin, L. H. Zhou, K. Song, S. H. Qian, D. M. Lin, L. Zhao, B. Chen, E. R. Yan, R. Michalet, and Y. C. Yang. 2021. "Benefit versus cost trade-offs of masting across seed-to-seedling transition for a dominant subtropical forest species." Journal of Ecology 109 (8):3087-3098. doi: 10.1111/1365-2745.13722. |
| 2579 | Shestakova, T. A., S. Mutke, J. Gordo, J. J. Camarero, E. Sin, J. Peman, and J. Voltas. 2021. "Weather as main driver for masting and stem growth variation in stone pine supports compatible timber and nut co-production." Agricultural and Forest Meteorology 298:14. doi: 10.1016/j.agrformet.2020.108287. |
| 2580 | Tissier, Mathilde L., Denis Reale, Dany Garant, and Patrick Bergeron. 2020. "Consumption of red maple in anticipation of beech mast-seeding drives reproduction in eastern chipmunks." Journal of Animal Ecology 89 (5):1190-1201. doi: 10.1111/1365-2656.13183. |
| 2581 | B. Angoboy Ilondea, H. Beeckman, D.-Y. Ouédraogo, N. Bourland, T. De Mil, J. Van Den Bulcke, J. Van Acker, C. Couralet, C. Ewango, W. Hubau, B. Toirambe, J.-L. Doucet, A. Fayolle. 2019. Une forte saisonnalité du climat et de la phénologie reproductive dans la forêt du Mayombe : l’apport des données historiques de la Réserve de Luki en République démocratique du Congo. Bois et Forêts des Tropiques, 341, 39-53, 10.19182/bft2019.341.a31753 |
| 2582 | Poncet, B.N., Garat, P., Manel, S., Bru, N., Sachet, J-M., Roque, A., Despres, L. 2009. The effect of climate on masting in the European larch and on its specific seed predators, Oecologia, 159, 527-537 |
| 2583 | Sanchez-Mejia, María Teresa. 2020. Crop production, recruitment and survival in Quercus ilex: density-dependent effects in a mast-seeding species. Unpublished MSc thesis. |
| 2584 | Khanduri, V.P., Kumar, K.S. Sharma, C.M., Riyal, M.K., Kar, K. (2019) Pollen limitation and seed set associated with year-to-year variation in flowering of Gmelinaarborea in a natural tropical forest, Grana, 58:2, 133-143, DOI: 10.1080/00173134.2018.1536164 |
| 2585 | Nakahata, R., M. Naramoto, M. Sato, and H. Mizunaga. 2021. Multifunctions of fine root phenology in vegetative and reproductive growth in mature beech forest ecosystems. Ecosphere 12(10):e03788. 10.1002/ecs2.3788 |
| 3001 | Abteilung Waldbau I b der Forstlichen Bundesversuchsanstalt Mariabrunn in Schönbrunn. 1960. Waldsamen-Ernteaussichten für 1960/61. Fachzeitschrift für das gesamte Forstwesen; Mitteilungsbl. D. forstl. Forstvereine u. Standesorganisation Österreichs.- Wien: Österr. Agrarverlag. Band 71 (19-20): 225-226. |
| 3002 | Prognosen der Waldsamenernte. Allgemeine Forstzeitschrift. |
| 3003 | unpublished data |
| 3004 | Andersen, S. T. 1980. Influence of climatic variation on pollen season severity in wind-pollinated trees and herbs. Grana. 19(1): 47-52. |
| 3005 | Anderson, M. L. 1949. Some observations on Belgian forestry. Empire Forestry Review. 28(2): 117-130. |
| 3006 | Andersson, E. 1965. Cone and seed studies in Norway spruce (Picea abies (L.) Karst.). Studia Forestalia Suecica. 23: 1-278. |
| 3007 | Agenzia regionale per la protezione ambientale della Toscana (ARPAT). 2015. Dati concentrazioni pollini e spore fungine in Toscana - anni 1996-2015. (http://www.arpat.toscana.it/datiemappe/dati/dati-concentrazioni-pollini-e-spore-fungine-in-toscana) |
| 3008 | Ascoli, D., Castagneri, D., Valsecchi, C., Conedera, M. and G. Bovio. 2013. Post-fire restoration of beech stands in the Southern Alps by natural regeneration. Ecological Engineering. 54: 210-217. |
| 3009 | Ascoli, D., Vacchiano, G., Maringer, J., Bovio, G. and M. Conedera. 2015. The synchronicity of masting and intermediate severity fire effects favors beech recruitment. Forest Ecology and Management. 353: 126-135. |
| 3010 | Ascoli, D.; Personal observation |
| 3011 | Barnekow, L., Loader, N. J., Hicks, S., Froyd, C. A., and T. Goslar. 2007. Strong correlation between summer temperature and pollen accumulation rates for Pinus sylvestris, Picea abies and Betula spp. in a high‐resolution record from northern Sweden. Journal of Quaternary Science. 22(7): 653-658. |
| 3011 | Barnekow, L., Loader, N. J., Hicks, S., Froyd, C. A., and T. Goslar. 2007. Strong correlation between summer temperature and pollen accumulation rates for Pinus sylvestris, Picea abies and Betula spp. in a high‐resolution record from northern Sweden. Journal of Quaternary Science. 22(7): 653-658. |
| 3013 | Bastide la, J. G. A. and C. L. H. van Vredenburch. 1970. The influence of weather conditions on the seed production of some forest trees in the Netherlands. Mededeling, Stichting Bosbouwproefstation 'De Dorschkamp', Wageningen. |
| 3014 | Beling. 1877. Über die Samenjahre der Eiche, Buche und Fichte. In: Monatsschrift für das Forst- und Jagdwesen.- Stuttgart: Schweizerbart. Heft 21: 49-81. |
| 3015 | Belmonte, J., Alarcón, M., Avila, A., Scialabba, E. and D. Pino. 2008. Long-range transport of beech (Fagus sylvatica L.) pollen to Catalonia (north-eastern Spain). International journal of biometeorology. 52(7): 675-687. |
| 3016 | Bergstedt, B. O. 1965. Distribution, reproduction, growth and dynamics of the rodent species Clethrionomys glareolus (Schreber), Apodemus flavicollis (Melchior) and Apodemus sylvaticus (Linne) in southern Sweden. Oikos. 16:132-160. |
| 3017 | Beudert, B. and H. Dieffenbach-Fries. 2016. UNECE Integrated Monitoring programme in the Forellenbach area of the Bavarian Forest National park - litterfall data from ongoing monitoring activities. On behalf of the German Environment Agency (UBA), Grafenau (Germany). |
| 3019 | Department of Forest Genetics, Federal Research and Training Centre for Forests, Natural Hazards and Landscape (BFW). 2015. Pollen- Samenproduktion österreichischer Waldbäume. (http://bfw.ac.at/rz/pollen.main?bart_in=01.0&jahr_in=2009) |
| 3020 | Bieber, C. and T. Ruf. 2005. Population dynamics in wild boar Sus scrofa: ecology, elasticity of growth rate and implications for the management of pulsed resource consumers. Journal of Applied Ecology. 42(6): 1203-1213. |
| 3021 | Bílek, L., Remeš, J. and D. Zahradník. 2009. Natural regeneration of senescent even-aged beech (Fagus sylvatica L.) stands under the conditions of Central Bohemia. Journal of Forest Science. 55: 145-155. |
| 3022 | Bisi, F., von Hardenberg, J., Bertolino, S., Wauters, L. A., Imperio, S., Preatoni, D. G., Provenzale A., Mazzamuto M. V., and A. Martinoli. 2016. Current and future conifer seed production in the Alps: testing weather factors as cues behind masting. European Journal of Forest Research. 135(4): 743-754. |
| 3023 | Bjedov, L., Svoboda, P., Tadin, A., Habuš, J., Štritof, Z., Labaš, N., Vucelja, M., Marcotić, A., Turk, N., and J. Margaletić. 2016. Utjecaj uroda sjemena obične bukve (Fagus sylvatica l.) na populacije sitnih glodavaca i pojavnosti hantavirusa u šumama nacionalnog parka “Plitvička Jezera” i parka prirode “Medvednica”. Šumarski list. 140(9-10): 455-463. |
| 3024 | Bundesanstalt für Landwirtschaft und Ernährung. Referat 324- Wald und Holz, Waldklimafonds. Bonn (Germany). Archive data. |
| 3025 | Borchers, K. 1958. Auswirkungen rezenter Klimaschwankungen auf die Häufigkeit von Buchen-Samenjahren in Niedersachsen. Forst-und Holzwirtschaft. 13: 330. |
| 3026 | Bouchard, M., Régnière, J., & Therrien, P. (2018). Bottom-up factors contribute to large-scale synchrony in spruce budworm populations. Canadian Journal of Forest Research, 48(3), 277-284. |
| 3027 | Braun, H. U.; Personal observation |
| 3028 | Broome, A., Hendry, S. and A. Peace. 2007. Annual and spatial variation in coning shown by the Forest Condition Monitoring programme data for Norway spruce, Sitka spruce and Scots pine in Britain. Forestry. 80(19): 17-28. |
| 3029 | Burckhardt, H. 1875. Das Mastjahr 1875. Von den Niedersächsischen Landesforsten: Schriftreihe "Aus dem Walde". 255-264. |
| 3030 | Burkart, A.; Personal observation |
| 3031 | Burri, A., Burkart, A., Moritzi, M., Moser, B., Wasem, U. and T. Wohlgemuth. 2016. Samenproduktion bei Waldbäumen: eine neue Webseite. Zürcher Wald. 1: 23-27. |
| 3032 | Burschel, P. 1966. Untersuchungen in Buchenmastjahren. Forstwissenschaftliches Centralblatt 85(7): 204-219. |
| 3033 | Survey of the "Foresta Demaniale Regionale del Cansiglio", Regione Veneto |
| 3034 | Čejková, A., and T. Kolář. 2009. Extreme radial growth reaction of Norway spruce along an altitudinal gradient in the Šumava Mountains. Geochronometria. 33(1): 41-47. |
| 3035 | CEMP long term dataset, shared by Prof C. Krebs |
| 3036 | Corpo Forestale dello Stato, Ministero delle Politiche Agricole, Alimentari e Forestali, Ufficio territoriale per la Biodiversità di Verona - CNBF di Peri, Italiano |
| 3037 | Conrad, B. 2005. Regenerationsdynamik buchendominierter Laubwälder auf Kalkstandorten. Inaugural-Dissertation zur Erlangung der Doktorwürde der Fakultät für Forst- und Umweltwissenschaften der Albert-Ludwigs-Universität Freiburg im Breisgau. |
| 3038 | Cornwallis, R. K. and A. D. Townsend. 1968. Waxwings in Britain and Europe during 1965/66. British Birds. 61: 97-118. |
| 3039 | Croatian forests, Ltd., Zagreb |
| 3040 | Cutini, A., Chianucci, F., and Giannini T. 2009. Effetti del trattamento selvicolturale su caratteristiche della copertura, produzione di lettiera e di seme in cedui di faggio in conversione. Annals of Silvicultural Research. (36): 109-124. |
| 3041 | Cutini, A., Chianucci, F., Chirichella, R., Donaggio, E., Mattioli, L. and M. Apollonio. 2013. Mast seeding in deciduous forests of the northern Apennines (Italy) and its influence on wild boar population dynamics. Annals of Forest Science. 70(5): 493-502. |
| 3042 | de Wavrin, H., Walravens, M. and D. Rabosee. 1991. Nidiﬁcations exceptionnelles du Hibou moyen-duc (Asio otus) et du Faucon Crécerelle (Falco tinnunculus) en 1991 en forêt de Soignes (Brabant). Aves. 28(4): 169-188. |
| 3043 | Dengler. A. 1944. Waldbau auf ökologischer Grundlage, ed. Springer-Verlag, Berlin and Heidelberg. |
| 3045 | Dobrowolska, D. 2015. Vitality of European beech (Fagus sylvatica L.) at the limit of its natural range in Poland. Polish Journal of Ecology. 63: 260-272. |
| 3046 | unpublished data |
| 3047 | Drobyshev, I., Niklasson, M., Mazerolle, M. J., and Y. Bergeron. 2014. Reconstruction of a 253-year long mast record of European beech reveals its association with large scale temperature variability and no long-term trend in mast frequencies. Agricultural and Forest Meteorology. 192: 9-17. |
| 3048 | EAN - European Aerobiological Network - Austria |
| 3049 | EGI - Edward Grey Institute of Field Ornithology, University of Oxford |
| 3050 | Eichhorn, J., Dammann, I., Schönfelder, E., Albrecht, M., Beck, W. and U. Paar. 2008. Untersuchungen zur Trockenheitstoleranz der Buche am Beispiel des witterungsextremen Jahres 2003. Beiträge aus der BW-FVA. Band 3. |
| 3051 | Feichtner, B. 1998. Ursachen der Streckenschwankungen beim Schwarzwild im Saarland. Zeitschrift für Jagdwissenschaft. 44(3): 140-150. |
| 3052 | Flade, M. and J. Schwarz. 2004. Ergebnisse des DDA-Monitoringprogramms, Teil II: Bestandsentwicklung von Waldvögeln in Deutschland. Vogelwelt. 125:177-213. |
| 3053 | Forst Thuringia unoublished data: C. Rösner, Referat Forstsaatgutberatung, forstliches Forschungs- und Kompetenzzentrum Gotha, Referat Monitoring, Klima und Forschung, Gotha (e-mail 2016-05-10) |
| 3054 | Zamorano, J.G., Hokkanen, T., and A. Lehikoinen. 2016. Climate driven synchrony in seed production of masting deciduous and conifer tree species. Journal of Plant Ecology. rtw117. |
| 3054 | Zamorano, J.G., Hokkanen, T., and A. Lehikoinen. 2016. Climate driven synchrony in seed production of masting deciduous and conifer tree species. Journal of Plant Ecology. rtw117. |
| 3055 | Gerasimidis, A., Panajiotidis, S., Hicks, S., and N. Athanasiadis. 2006. An eight-year record of pollen deposition in the Pieria mountains (N. Greece) and its significance for interpreting fossil pollen assemblages. Review of Palaeobotany and Palynology 141(3):231-243. |
| 3056 | Gloaguen, J. C. and J. Touffet. 1982. Production de litière dans une chenaie-hetraie Atlantique. Relations avec les caracteres climatiques. Revue forestière française. 34(2): 108-118. |
| 3057 | Granier, A., Bréda, N., Longdoz, B., Gross, P. and J. Ngao. 2008. Ten years of fluxes and stand growth in a young beech forest at Hesse, North-eastern France. Annals of Forest Science. 64(704): 1-13. |
| 3058 | Gross, H. 1934. Die Rotbuche in Ostpreussen. Zeitschrift für Forst-und Jagdwesen. 66/12: 622-651. |
| 3059 | Grulois, C., De Meersman, F., Loyen, S., Orfinger, C., Quivy, V., De Vos, B. and B. Van der AA. 2001. Régénération naturelle de la Forêt de Soignes. Rapport Final. Instituut voor Bosbouw en Wildbeheer and Centre de Recherches Agronomiques de Gembloux. |
| 3060 | Guggisberg, M., 2002. Walddynamik der letzten 70 Jahre bei Ponte Tresa/Tessin, Südalpen festgehalten in gewarvten Seesedimenten. MSc Thesis Phil. Nat. Fac. University of Bern, p. 83. |
| 3061 | Guitián, M. A. and F. J. da Costa. 2005. Primeros datos sobre la variabilidad interanual de la producción de semilla de Fagus sylvatica L. en el extremo occidental de la Cornisa Cantábrica. In Proceedings IV Congreso Forestal Nacional. SECF-DGA. 26-27 September Zaragoza, ES. |
| 3062 | Gurnell, J. 1993. Tree seed production and food conditions for rodents in an oak wood in southern England. Forestry. 66(3): 291-315. |
| 3064 | Hansson, L. 1971. Small rodent food, feeding and population dynamics. A comparison between granivorous and herbivorous species in Scandinavia. Oikos. 22(2): 183-198. |
| 3065 | Haredasht, S. A., Taylor, C. J., Maes, P., Verstraeten, W. W., Clement, J., Barrios, M., Lagrou, K., Van Ranst, M., Coppin, P., Berckmans, D. and J. M. Aerts. 2013. Model‐based prediction of Nephropathia epidemica outbreaks based on climatological and vegetation data and bank vole population dynamics. Zoonoses and Public Health. 60(7): 461-477. |
| 3066 | Hartig, R. 1889. Über den Einfluß der Samenproduktion auf Zuwachsgrösse und Reservestoffvorrathe der Bäume . Allg. Forst Jagdztg. 65, pp. 13–17 |
| 3067 | Hase, W. 1964. Die Buchenmast in Schleswig-Holstein und ihre Abhängigkeit von der Witterung. Mitt. Deutsch. Wetterdienst. 31: 31/3-31/45 |
| 3068 | Herbst, M., Mund, M., Tamrakar, R., and A. Knohl. 2015. Differences in carbon uptake and water use between a managed and an unmanaged beech forest in central Germany. Forest Ecology and Management 355: 101-108. |
| 3069 | Herget, K.; Personal observation |
| 3070 | Hermansson S., Cato N., and U. Lamberth U. 2014. Skogsstyrelsen. Online: http://www.skogsstyrelsen.se/Myndigheten/Press-och-information/Pressmeddelanden1/Pressrelease/?releaseId=1643579 |
| 3071 | Heroldová, M., Suchomel, J., Purchart, L. and L. Čepelka. 2013. Beech-mast crop evaluation in Kněhyně forest complex (Beskydy Mts. Czech Republic) as a food supply for granivorous rodents. Beskydy. 6(1): 27-32. |
| 3072 | Hess, R. A. 1905. Die Eigenschaften und das forstliche Verhalten der wichtigeren in Deutschland vorkommenden Holzarten: Ein Leitfaden für Studierende, Praktiker und Waldbesitzer. Parey, Berlin. |
| 3073 | Heyman, P., Thoma, B. R., Marié, J. L., Cochez, C. and S. S. Essbauer. 2012. In search for factors that drive hantavirus epidemics. Frontiers in Physiology. 3(237): 1-23. |
| 3074 | Hilton, G. M. and J. R. Packham. 2003. Variation in the masting of common beech (Fagus sylvatica L.) in northern Europe over two centuries (1800–2001). Forestry. 76(3): 319-328. |
| 3075 | Hoch, G., Siegwolf, R. T., Keel, S. G., Körner, C. and Q. Han. 2013. Fruit production in three masting tree species does not rely on stored carbon reserves. Oecologia. 171(3): 653-662. |
| 3076 | Hoelzl, F., Bieber, C., Cornils, J. S., Gerritsmann, H., Stalder, G. L., Walzer, C. and T. Ruf. 2015. How to spend the summer? Free-living dormice (Glis glis) can hibernate for 11 months in non-reproductive years. Journal of Comparative Physiology. 185(8): 931-939. |
| 3077 | Hofmann, G., Anders, S., Beck, W., Chzron, S. and B. Matthes. 1992. Buchenwälder in der ehemaligen DDR und ihr Vitalitätszustand. NZ NRW-Seminar. 12: 23-34. |
| 3079 | Holmsgaard, E., and H. C. Olsen. 1960. The influence of weather on beech mast. Forstlige Forsøgsvaesen I Danmark. 26: 347-370. |
| 3080 | Huss, J. 1964. Untersuchungen über die natürliche Verjüngung der Buche: die Entwicklung des Aufschlages nach der Mast 1960. Dissertation der Forstwissenschaftlichen Fakultät der Georg-AugustUniversität zu Göttingen in Hann. Münden, 187 S. |
| 3081 | Huss, J., Kratsch, H. D. and E. Röhrig. 1972. Ein Erfahrungsbericht über Maßnahmen zur Förderung der Buchennaturverjüngung, bei der Mast 1970 in acht Forstämtern Südniedersachsens. Forst- und Holzwirt. 27: 365-370. |
| 3082 | Hyde, H. A. 1963. Pollen-fall as a means of seed prediction in certain trees. Grana. 4(2): 217-230. |
| 3083 | Ihrig. 1860. Ueber Wiederkehr der Mastjahre. Natürliche Verjüngung und Eckernertrag in Buchenhochwaldungen. Allgemeine Forst- und Jagdzeitung, 36. Jg., H. September, S. 341-350 |
| 3084 | Jacamon, M. 1987. Le parc de l'École forestière à Nancy (France). Bulletin de la Société Botanique de France. Lettres Botaniques. 134(1): 29-34. |
| 3085 | Jacobsen, E. M. 2001. Punkttællinger af ynglefugle i eng, by og skov 2000: Naturovervågning. Danmarks Miljøundersøgelser, Aarhus Universitet. |
| 3086 | Jenni, L. 1987. Mass concentration of Bramblings Fringilla montifringilla in Europe 1900–1983: Their dependence upon beech mast and the effect of snow cover. Ornis Scandinavica. 18: 84-94. |
| 3088 | Juday, G., Barber, V., & Zasada, J. (2003). A 200‐year perspective of climate variability and the response of white spruce in Interior Alaska. |
| 3089 | Kager, T. and J.Fietz. 2009. Food availability in spring influences reproductive output in the seed-preying edible dormouse (Glis glis). Canadian Journal of Zoology. 87(7): 555-565. |
| 3090 | Källander, H. 1993. Food caching in the European nuthatch Sitta europaea. Ornis Svecica 3(2): 49-58. |
| 3091 | Kämpfer-Lauenstein, A. and W. Lederer. 2010. Populationsdynamik des Raufußkauzes Aegolius funereus im Arnsberger Wald. Charadrius. 46(1-2): 69-78. |
| 3092 | Kaplunovskyy С. 1972. Особенности плодоношения буковых лесов. Лесоведение 1:51-61. |
| 3095 | Keller, H. 1875. Waldsamenernte von 1874/75. Allgemeine Forst- and Jagdzeitung. 33. |
| 3095 | Keller, H. 1875. Waldsamenernte von 1874/75. Allgemeine Forst- and Jagdzeitung. 33. |
| 3096 | Kisházi, Z. 1982. Makktermés utáni vizsgálatok véghasználati bükkösökben. Erdészeti Lapok. 31 (117.): 393-396. |
| 3097 | Kleef, H. L. and H.J. Wijsman. 2015. Mast, mice and pine marten (Martes martes): the pine marten’s reproductive response to wood mouse (Apodemus sylvaticus) fluctuations in the Netherlands. Lutra. 58(1): 23-33. |
| 3098 | Klhare J, Roth H.U. 1996. Nutritional preferences of Abruzzo brown bears before hibernation. Proceedings of the II Conferenze of the Italian Association of Teriologia, III Italian Simposium on Carnivorous. Perugia, October 1996. |
| 3099 | Konnert, M., Schneck, D., and Zollner, A. (2014). Blühen und Fruktifizieren unserer Waldbäume in den letzten 60 Jahren. LWF Wissen, 74, 37-45. |
| 3100 | Konnert, M.; Personal observation |
| 3101 | CEMP long term dataset, shared by Prof C. Krebs |
| 3101 | CEMP long term dataset, shared by Prof C. Krebs |
| 3101 | CEMP long term dataset, shared by Prof C. Krebs |
| 3101 | CEMP long term dataset, shared by Prof C. Krebs |
| 3102 | Kulzer, E., von Lindeiner-Wildau, A. and I.-M. Wolters. 1993. Säugetiere im Naturpark Schönbuch. Landesanstalt für Umweltschutz Baden-Württemberg, Karlsruhe. |
| 3103 | Latte, N., Lebourgeois, F. and H. Claessens. 2016. Growth partitioning within beech trees (Fagus sylvatica L.) varies in response to summer heat waves and related droughts. Trees. 30: 189–201. |
| 3104 | Lauprecht, G . 1874. Buchen- und Eichen-Samenjahre im Vergleich mit der Witterung. Zeitschrift für Forst- und Jagdwesen. 7: 246-266 |
| 3105 | Le Tacon, F., Oswald, H., Perrin, R., Picard, J. F. and J.P. Vincent. 1976. Les causes de l'échec de la régénération naturelle du hêtre à la suite de la fainée de 1974. Revue forestière française. 28(6): 426-446. |
| 3107 | Lebret, M., Nys, C. and F. Forgeard. 2001. Litter production in an Atlantic beech (Fagus sylvatica L.) time sequence. Annals of Forest Science. 58(7): 755-768. |
| 3108 | Leemans, R. 1991. Canopy gaps and establishment patterns of spruce (Picea abies (L.) Karst.) in two old-growth coniferous forests in central Sweden. Vegetatio. 93(2): 157-165. |
| 3109 | LFD - Landesforstdirektion Südtirol |
| 3110 | Liesebach, M. 2012. Der Internationale Herkunftsversuch mit Rot-Buche von 1993/95–Beschreibung der ausgewählten sechs Herkünfte und zwei Versuchsflächen. Landbauforschung. 62(4): 159-168. |
| 3111 | Lithner, S., and K.I. Jonsson. 2002. Abundance of owls and bramblings Fringilla montifringilla in relation to mast seeding in south-eastern Sweden. Ornis Svecica. 12(1-2): 35-45. |
| 3112 | Litschauer, R. 2001. Blüh-und Fruktifikationsverhalten der Waldbäume. FBVA—Berichte. 123: 45-66. |
| 3113 | Ljungström, M., Gyllin, M. and B. Nihlgård. 1990. Effects of liming on soil acidity and beech (Fagus sylvatica L.) regeneration on acid soils in south Swedish beech forests. Scandinavian Journal of Forest Research. 5(1-4): 243-254. |
| 3114 | Le Louarn, H. and A. Schmitt. 1972. Relations observées entre la production de Faines et la dynamique de population du Mulot, Apodemus sylvaticus L. en forêt de Fontainebleau. In Annales des Sciences Forestieres. 29(2): 205-214. |
| 3115 | Madsen, P. 1995. Effects of seedbed type on wintering of beech nuts (Fagus sylvatica) and deer impact on sprouting seedlings in natural regeneration. Forest Ecology and Management. 73(1): 37-43. |
| 3116 | Majer, A. 1982. A bükkösök makk termésének időszakossága. Erdészeti Lapok. 31(9): 388-392. |
| 3117 | Mancini N.M., Mancini G.M., Travaglini D., Nocentini S. and R. Giannini. 2016. Prime osservazioni sulla struttura e la produzione di seme dei boschi cacuminali di faggio nei Monti della Laga (Parco Nazionale del Gran Sasso e dei Monti della Laga). L’Italia Forestale e Montana. 71 (1): 31-47 |
| 3118 | Marteau, M. and M. Sarà. 2015. Habitat preferences of edible dormouse, Glis glis italicus: implications for the management of arboreal mammals in Mediterranean forests. Folia Zoologica 64 (2): 136-150 |
| 3119 | Martin-DeMoor, J., V. J. Lieffers, and S. E. Macdonald. 2010. Natural regeneration of white spruce in aspen-dominated boreal mixedwoods following harvesting. Canadian Journal of Forest Research-Revue Canadienne De Recherche Forestiere 40:585-594. |
| 3120 | Martin, H. 2009. Caractérisation des fructifications des chênaies et hêtraies du réseau Renecofor. Diplôme de l’École Pratique des Hautes Études. |
| 3121 | Martínez, I. and F. González-Taboada. 2009. Seed dispersal patterns in a temperate forest during a mast event: performance of alternative dispersal kernels. Oecologia. 159(2): 389-400. |
| 3122 | Forschungsanstalt für Wald, Schnee und Landschaft WSL http://www.wsl.ch/mastweb/resultate/index_DE |
| 3123 | Matic, S., Orsanic, I. and I. Anic. 2003. Establishing forests of Common beech. In: Common beech (Fagus sylvatica L.) in Croatia. Academy of Forestry Sciences. 326-339. |
| 3124 | Matthysen, E. 1989. Nuthatch Sitta europaea demography, beech mast, and territoriality. Ornis Scandinavica. 20(4):278-282. |
| 3125 | Maurer, E. 1964. Buchen-und Eichensamenjahre in Unterfranken wahrend der letzten 100 Jahre. Allgemeine Forstzeitschrift. 31: 469-470. |
| 3126 | Meiffren, I. 1988. Airborne pollen of Toulouse, southern France: comparison with Bordeaux and Montpellier. Grana. 27(3):183-201. |
| 3127 | Mellström, G. 1918. Skogsträdens frösättning år 1917. Statens Skogsforsoksanstalt. 15 :43-68. |
| 3128 | Mencuccini, M., and P. Piussi. 1995. Production of seed and cones and consequences for radial increment in Norway spruce (Picea abies (L.) Karst.). Giornale Botanico Italiano. 129(3): 797-812. |
| 3129 | Mencuccini, M., Piussi, P., and A. Z. Sulli. 1995. Thirty years of seed production in a subalpine Norway spruce forest: patterns of temporal and spatial variation. Forest Ecology and Management. 76(1): 109-125. |
| 3130 | Federal Office of Meteorology and Climatology (MeteoSwiss), Switzerland |
| 3131 | Mezzavilla F. 2014. Il faggio e la fauna. Indagini ecologiche nella riserva naturale biogenetica campo di mezzo - Pian Parrocchia. Foresta del Cansiglio. Ministero delle Politiche Agricole e Forestali. |
| 3132 | Michaelis, 1911. Einiges zur Buchenmast 1909. Zeitschrift f. Forst- und Jagdwesen. 43: 267-283. |
| 3133 | Millerón, M., De Heredia, U. L., Lorenzo, Z., Perea, R., Dounavi, A., Alonso, J., Gil, L. and N. Nanos. 2012. Effect of canopy closure on pollen dispersal in a wind-pollinated species (Fagus sylvatica L.). Plant Ecology. 213(11): 1715-1728. |
| 3134 | Mašková, P. and P. Adamík. 2012. Poznámky o výskytu arborealních hlodavců (Mammalia: Rodentia) v budkách na Sovinecku, Nízký Jeseník. Zprávy Vlastivědného muzea v Olomouci. 303: 13–21. |
| 3135 | Mountford, E. P., Savill, P. S. and D.P. Bebber. 2006. Patterns of regeneration and ground vegetation associated with canopy gaps in a managed beechwood in southern England. Forestry. 79(4): 389-408. |
| 3136 | Müller-Haubold, H., Hertel, D. and C. Leuschner. 2015. Climatic drivers of mast fruiting in European Beech and resulting C and N allocation shifts. Ecosystems. 18(6): 1083-1100. |
| 3137 | Mund, M., Kutsch, W. L., Wirth, C., Kahl, T., Knohl, A., Skomarkova, M. V. and E.D. Schulze. 2010. The influence of climate and fructification on the inter-annual variability of stem growth and net primary productivity in an old-growth, mixed beech forest. Tree Physiology. 30: 689-704. |
| 3138 | Neckařová, M. 2012. Velkoplošná variabilita plodování buku lesního: semenné roky a prostorová synchronie. Univerzita Palackého v Olomouci. |
| 3139 | Newson, R. 1963. Differences in numbers, reproduction and survival between two neighboring populations of bank voles (Clethrionomys glareolus). Ecology. 44(1): 110-120. |
| 3141 | Nielsen, J. T. 2005. Yngletidspunktets betydning for produktionen af unger og deres overlevelse hos Spurvehøgen Accipiter nisus i Vendsyssel 1977-97. Dansk Orn. Foren. Tidsskr. 99: 107-114. |
| 3142 | Nielsen, A. B., Møller, P. F., Giesecke, T., Stavngaard, B., Fontana, S. L. and R. H. Bradshaw. 2010. The effect of climate conditions on inter-annual flowering variability monitored by pollen traps below the canopy in Draved Forest, Denmark. Vegetation History and Archaeobotany. 19(4): 309-323. |
| 3143 | Niklasson, M. 2003. En undersökning av trädåldrar i halländska skogsreservat. Information från. Länsstyrelsen halland. |
| 3145 | Nilsson, S. G. 1985. Ecological and evolutionary interactions between reproduction of beech Fagus silvatica and seed eating animals. Oikos. 44(1): 157-164. |
| 3146 | Nopp‐Mayr, U., Kempter, I., Muralt, G. and G. Gratzer. 2012. Seed survival on experimental dishes in a central European old‐growth mixed‐species forest–effects of predator guilds, tree masting and small mammal population dynamics. Oikos. 121(3):337-346. |
| 3147 | Nováková, P., Štípek, K., Ježek, M., Červený, J. and V. Ešner. 2011. Effect of diet supply and climatic conditions on population dynamics of the wild boar (sus scrofa) in the křivoklát region (Central Bohemia,Czech Republic). Scientia Agriculturae Bohemica. 42(1): 24-30. |
| 3148 | Nussbaumer, A., Waldner, P. and S. Etzold. 2014. Fruiting occurrence of Beech, Spruce, Pine and Oak trees in Europe. Report I. Swiss Federal Institute for Forest, Snow and Landscape Research WSL, Birsmendorf. |
| 3149 | Nussbaumer, A., Waldner, P., Etzold, S., Gessler, A., Benham, S., Thomsen, I. M., Jørgensen, B.B., Timmermann, V., Verstraeten, A., Sioen, g., Rautio, P., Ukonmaanaho, L., Skudnik, M., Apuhtin, V., Braun, S. and A. Wauer. 2016. Patterns of mast fruiting of common beech, sessile and common oak, Norway spruce and Scots pine in Central and Northern Europe. Forest Ecology and Management. 363: 237-251. |
| 3149 | Nussbaumer, A., Waldner, P., Etzold, S., Gessler, A., Benham, S., Thomsen, I. M., Jørgensen, B.B., Timmermann, V., Verstraeten, A., Sioen, g., Rautio, P., Ukonmaanaho, L., Skudnik, M., Apuhtin, V., Braun, S. and A. Wauer. 2016. Patterns of mast fruiting of common beech, sessile and common oak, Norway spruce and Scots pine in Central and Northern Europe. Forest Ecology and Management. 363: 237-251. |
| 3150 | Nussbaumer, A., Etzold, S., Waldner, P. and Dobbertin, M. Carbon allocation to fruits and seeds in European forests as a function of climate, atmospheric deposition and nutrient supply. COST Action FP0903: Climate Change and Forest Migration and Adaption in a Polluted Environment. (http://bfw.ac.at/cms_stamm/430/pdf/ffcc/EP14/07_Nussbaumer.pdf) Access 2016-07-01 |
| 3151 | Oddou-Muratorio, S., Bontemps, A., Klein, E. K., Chybicki, I., Vendramin, G. G. and Y. Suyama. 2010. Comparison of direct and indirect genetic methods for estimating seed and pollen dispersal in Fagus sylvatica and Fagus crenata. Forest Ecology and Management. 259(11): 2151-2159. |
| 3152 | Olesen, C. R., and P. Madsen. 2008. The impact of roe deer (Capreolus capreolus), seedbed, light and seed fall on natural beech (Fagus sylvatica) regeneration. Forest Ecology and Managemen.t 255(12): 3962-3972. |
| 3153 | Ott, E., Conceprio, F. and A. Pedrini. 2003. Prime valutazioni sull'introduzione della rinnovazione naturale nel bosco ceduo di castagno misto a faggio nella foresta sperimentale e didattica della SPF di Zurigo a Novaggio, Cantone Ticino. Schweizerische Zeitschrift fur Forstwesen. 154(2):51-67. |
| 3154 | Övergaard, R., Gemmel, P. and M. Karlsson. 2007. Effects of weather conditions on mast year frequency in beech (Fagus sylvatica L.) in Sweden. Forestry. 80(5): 555-565. |
| 3155 | Overgaard, R.; Personal observation |
| 3156 | Paar, U., Guckland, A., Dammann, I., Albrecht, M. and J. Eichhorn. 2011. Häufigkeit und Intensität der Fruktifikation der Buche. AFZ-Der Wald. 6: 26-29. |
| 3157 | Pakenham, R. 1996. Natural regeneration of beech in the Chilterns. Quarterly Journal of Forestry. 90(2): 143-149. |
| 3158 | Palaghianu, C.; Personal observation |
| 3159 | Parmigiani, S. 2007. Spatial behaviour of the yellow-necked mouse (Apodemus flavicollis, melchior 1834) at contrasting population density and resource availability. P.h.D Thesis, University of Parma. |
| 3160 | Perdeck, A. C., Visser, M. E. and J. H. Van Balen. 2000. Great tit Parus major survival and the beech-crop. Ardea. 88: 99-106. |
| 3161 | Perrin, R. 1978. Etude de la sporulation de Nectria Ditissima Tul. Agent du chancre du hêtre. Annales des Sciences Forestières. 35(3): 213-228. |
| 3162 | Perrins, C. M. 1965. Population fluctuations and clutch-size in the Great Tit, Parus major L. The Journal of Animal Ecology. 34(3): 601-647. |
| 3163 | Peters, V. S., S. E. MacDonald, and M. R. T. Dale. 2005. The interaction between masting and fire is key to white spruce regeneration. Ecology 86:1744-1750. |
| 3164 | Petty, S. J., Patterson, I. J., Anderson, D. I. K., Little, B., and M. Davison. 1995. Numbers, breeding performance, and diet of the sparrowhawk Accipiter nisus and merlin Falco columbarius in relation to cone crops and seed-eating finches. Forest ecology and management. 79(1): 133-146. |
| 3165 | Pidek, I. A., Svitavská-Svobodová, H., van der Knaap, W. O., Noryśkiewicz, A. M., Filbrandt-Czaja, A., Noryśkiewicz, B., Filbrandt-Czaja, A., Noryśkiewicz, B., Latałowa, M., Zimny, M., Święta-Musznicka, J., Bozilova, E., Tonkov, S., Filipova-Marinova, M., Poska, A., Giesecke, T. and A. Gikov. 2010. Variation in annual pollen accumulation rates of Fagus along a N–S transect in Europe based on pollen traps. Vegetation History and Archaeobotany. 19(4): 259-270. |
| 3166 | Pilastro, A., Tavecchia, G. and G. Marin. 2003. Long living and reproduction skipping in the fat dormouse. Ecology. 84(7): 1784-1792. |
| 3167 | Pilegaard, K., Ibrom, A., Courtney, M. S., Hummelshøj, P. and N. O. Jensen. 2011. Increasing net CO 2 uptake by a Danish beech forest during the period from 1996 to 2009. Agricultural and Forest Meteorology. 151(7): 934-946. |
| 3168 | Piovesan, G. and M. Bernabei. 1997. L’influenza delle precipitazioni estive sulla crescita e la riproduzione del faggio (Fagus sylvatica L.) in una stazione meridionale dell’areale. Italia Forestale e Montana. 6: 444-459. |
| 3169 | Piussi. P, Istituto di Selvicoltura, Facoltà di Agraria, Università di Firenze; unpublished data |
| 3171 | Potena, G., Di Marzio. M., Panella, M., Sammarone, L., Altea, T., Posillico, M., Roman, M., and M. Consalvo. 2009. Il monitoraggio delle produzione di faggiola: una risorsa trofica critica per l’orso bruno (Ursus arctos). VII |
| 3172 | Prach, K., J. Lepš, and J. Michalek. 1996. Establishment of Picea abies seedlings in a central European mountain grassland: an experimental study. Journal of Vegetation Science. 7: 681-684. |
| 3173 | Pucek, Z., Jędrzejewski, W., Jędrzejewska, B., and M. Pucek. 1993. Rodent population dynamics in a primeval deciduous forest (Białowieża National Park) in relation to weather, seed crop, and predation. Acta Theriologica. 38(2): 199-232. |
| 3174 | Pukkala, T., Hokkanen, T., and T. Nikkanen. 2010. Prediction models for the annual seed crop of Norway spruce and Scots pine in Finland. Silva Fennica. 44(4): 629-642. |
| 3175 | Purdy B.G., Macdonald S.E., Dale M.R. T. (2002). The regeneration niche of white spruce following fire in the mixedwood boreal forest. Silva Fennica vol. 36 no. 1 article id 564. |
| 3176 | Regierungspräsidium Freiburg |
| 3176 | Regierungspräsidium Freiburg |
| 3177 | Relazioni annuali della Sezione Forestale del Canton Ticino. Dipartimento del territorio, Repubblica e Cantone del Ticino, Bellinzona. |
| 3178 | French National Forest Office, National Network for Long-term FORest ECOsystem Monitoring |
| 3180 | Röhrig, E., Bartels, H., Gussone, H. A. and B. Ulrich. 1978. Untersuchungen zur natürlichen Verjüngung der Buche (Fagus sylvatica). Forstwissenschaftliches Centralblatt. 97/1: 121-131. |
| 3181 | Roland, C. A., J. H. Schmidt, and J. F. Johnstone. 2014b. Climate sensitivity of reproduction in a mast-seeding boreal conifer across its distributional range from lowland to treeline forests. Oecologia 174:665-677. |
| 3182 | Romanian National Forest Administration - Romsilva, Suceava County Division |
| 3183 | Rossa, G. 1992. Geschichtliche Entwicklung der Laubholzbestände. Heimatland Lippe. 85(3): 90. |
| 3184 | Rossi, S., H. Morin, F. Gionest, and D. Laprise. 2012. Episodic recruitment of the seedling banks in balsam fir and white spruce. American Journal of Botany 99:1942-1950. |
| 3186 | Salmaso, F., Molinari, A., Di Pierro, E., Ghisla, A., Martinoli, A., Preatoni, D., Serino, G., Tosi, G., Bertolino, S. and L. A. Wauters. 2009. Estimating and comparing food availability for tree‐seed predators in typical pulsed‐resource systems: Alpine conifer forests. Plant Biosystems. 143(2): 258-267. |
| 3187 | Schlund, W., Scharfe, F. and J. U. Ganzhorn. 2002. Long-term comparison of food availability and reproduction in the edible dormouse (Glis glis). Mammalian Biology-Zeitschrift für Säugetierkunde. 67(4): 219-232. |
| 3188 | Schmidt, W. 2006. Zeitliche Veränderung der Fruktifikation bei der Rotbuche (Fagus sylvatica) in einem Kalkbuchenwald (1981-2004). Allgemeine Forst- u. Jagd-Zeitung. 177: 9-19. |
| 3189 | Schneck, R.; unpublished data |
| 3190 | Schwappach, A. 1895. Die Samenproduktion der wichtigsten Waldholzarten in Preussen. Zeitschrift für Forst- und Jagdwesen. 27: 147-174. |
| 3191 | Königlich Preußischer Forstmeiser Schwarz von Erfurt. 1870. Ein Beitrag zur Buchenwirtschaft im höherem Gebirge des Thüringer Waldes. Allgemeine Forst- und Jagdzeitung. 55-95. |
| 3192 | Schwarz, A. C., Ranft, U., Piechotowski, I., Childs, J. E. and S. O. Brockman. 2009. Risk factors for human infection with Puumala virus, Southwestern Germany. Emerging Infectious Diseases. 15(7): 1032-1039. |
| 3193 | Seeger. 1913. Ein Beitrag zur Samenproduktion der Waldbäume im Großherzogtum Baden. Naturwissenschaftliche Zeitschrift für Forst- und Landwirtschaft. 11: 529-554. |
| 3194 | Selås, V., Framstad, E., and T. K. Spidsø. 2002. Effects of seed masting of bilberry, oak and spruce on sympatric populations of bank vole (Clethrionomys glareolus) and wood mouse (Apodemus sylvaticus) in southern Norway. Journal of Zoology. 258(4): 459-468. |
| 3195 | Silva, D. E., Mazzella, P. R., Legay, M., Corcket, E. and J. L. Dupouey. 2012. Does natural regeneration determine the limit of European beech distribution under climatic stress? Forest Ecology and Management. 266: 263-272. |
| 3196 | Simoleit, A., Wachter, R., Gauger, U., Werchan, M., Werchan, B., Zuberbier, T. and K. C. Bergmann. 2016. Pollen season of European beech (Fagus sylvatica L.) and temperature trends at two German monitoring sites over a more than 30-year period. Aerobiologia (in press). DOI:10.1007/s10453-016-9421-y. |
| 3197 | Sioen, G., Roskams, P., Verschelde P., Van der Aa, B. and A. Verstraeten. 2008. Monitoring the masting behaviour of beech (Fagus sylvatica) in Flanders (Belgium). In Proceedings of the Forest Adaptation Conference, FAO. Session 1. Physiological responses of trees to climate. Poster presentation. Umeå, Sweden from 25-28 August 2008. |
| 3198 | Slovenian Forest Service (Zavod za Gozdovie Slovenije). 2016. Poročilo zavoda za gozdove slovenije o gozdovih. Za leto 2015. Ljubljana. |
| 3199 | Smith, M. C. (1968). Red squirrel responses to spruce cone failure in interior Alaska. The Journal of Wildlife Management, 305-317. |
| 3200 | Spek, G.J. and A. Vliet. 2015. Veel eikels aan de bomen, record aantal zwijnen in 2016 verwacht. (https://www.naturetoday.com/intl/nl/nature-reports/message/?msg=21765) Access Date 2016-07-01. |
| 3201 | Survey of the Public Forest Enterprise "Sume Republike Srpske" (Forests of the Republic of Srpska; http://www.sumers.org/portal/index.php) |
| 3202 | Staatsklenge Forstamt Nagold |
| 3203 | unpublished data |
| 3204 | Suchomel, J. 2014. A study of the synusia of small terrestrial mammals (Insectivora, Rodentia) of the Kelečská pahorkatina Upland–Czech Republic. Acta Universitatis Agriculturae et Silviculturae Mendelianae Brunensis. 55(5): 165-170. |
| 3205 | Suchomel, J., Purchart, L., Čepelka, L. and M. Heroldová. 2014. Structure and diversity of small mammal communities of mountain forests in Western Carpathians. European Journal of Forest Research. 133(3): 481-490. |
| 3206 | Svardson, G. 1957. The "invasion" type of bird migration. British Birds. 50: 314-343. |
| 3207 | Szewczyk, J. and J. Szwagrzyk. 2010. Spatial and temporal variability of natural regeneration in a temperate old-growth forest. Annals of Forest Science. 67(2): 202. |
| 3208 | Szwagrzyk, J., Gratzer, G., Stepniewska, H., Szewczyk, J. and B. Veselinovic. 2015. High reproductive effort and low recruitment rates of European beech: Is there a limit for the superior competitor?. Polish Journal of Ecology. 63: 198-212. |
| 3209 | Tacon, F. Le and C. V. Malphettes. 1974. Germination et comportement de semis de hetre sur six stations de la Foret demaniale de Villers-Cotterets. Revue Forestière Française. 26: 111-123. |
| 3210 | Tersago, K., Verhagen, R., Servais, A., Heyman, P., Ducoffre, G. and H. Leirs. 2009. Hantavirus disease (nephropathia epidemica) in Belgium: effects of tree seed production and climate. Epidemiology and Infection. 137(02): 250-256. |
| 3212 | Tirén, L., 1935. Om granens kottsättning, dess periodicitet och samband med temperatur och nederbör (On the fruit setting of spruce, its periodicity and relation to temperature and precipitation). Meddelanden Från Statens Skogsförsöksanst Häfte. 28(4): 413-521. |
| 3213 | Tollefsrud, M. M. 2015. Blomstring hos gran gir frø til skogbruket. Norsk institutt for biookonomi. (http://www.skogoglandskap.no/nyheter/2015/blomstring_hos_gran_gir_fro_til_skogbruket). Date of access 2016-06-27. |
| 3214 | Topoliantz, S. and J. F. Ponge. 2000. Influence of site conditions on the survival of Fagus sylvatica seedlings in an old‐growth beech forest. Journal of Vegetation Science. 11(3): 369-374. |
| 3215 | Tosoni, E., Gentile, D., Altea, T., Latini, R. and P. Ciucci. 2014. Conta cumulativa delle unità familiari di orso bruno marsicano per la stima della produttività della popolazione: estate 2014. UE Project LifeNAT/IT/000160 "ARCTOS", Dept. Biology and Biotechnologies, University of Rome "La Sapienza", Roma, Italy. |
| 3216 | Trauboth, V.; Personal observation |
| 3217 | Vacchiano, G.; Personal observation |
| 3218 | Vacek, S. and M. Hejcman. 2012. Natural layering, foliation, fertility and plant species composition of a Fagus sylvatica stand above the alpine timberline in the Giant (Krkonoše) Mts., Czech Republic. European Journal of Forest Research. 131(3): 799-810. |
| 3219 | Vacek, S. and A. Jurasek. 1986. Fruktifikace bukovych porostů pod vlivem imis. Opera Corcontica. 23: 111-142. |
| 3220 | Van der Knaap, W.O., van Leeuwen, J.F., Svitavská-Svobodová, H., Pidek, I. A., Kvavadze, E., Chichinadze, M., Giesecke, T., Kaszewski, B.M., Oberli, F., Kalnina, L., Pardoe, H. S., Tinner, W. and B. Ammann. S. 2010. Annual pollen traps reveal the complexity of climatic control on pollen productivity in Europe and the Caucasus. Vegetation History and Archaeobotany. 19(4): 285-307. |
| 3221 | Van der Maaten, E. 2012. Climate sensitivity of radial growth in European beech (Fagus sylvatica L.) at different aspects in southwestern Germany. Trees. 26(3): 777-788. |
| 3222 | Vanha-Majamaa, I., Tuittila, E. S., Tonteri, T., and R. Suominen. 1996. Seedling establishment after prescribed burning of a clear-cut and a partially cut mesic boreal forest in southern Finland. Silva Fennica. 30(1): 31-45. |
| 3223 | Wachter, H. 1964. Über die Beziehungen zwischen Witterung und Buchenmastjahren. Forstarchiv. 35(4): 69-78. |
| 3224 | Wauters, L. A., Vermeulen, M., Van Dongen, S., Bertolino, S., Molinari, A., Tosi, G. and E. Matthysen. 2007. Effects of spatio‐temporal variation in food supply on red squirrel Sciurus vulgaris body size and body mass and its consequences for some fitness components. Ecography. 30(1): 51-65. |
| 3225 | Wesołowski, T., Rowiński, P., and M. Maziarz. 2015. Interannual variation in tree seed production in a primeval temperate forest: does masting prevail?. European Journal of Forest Research. 134(1): 99-112. |
| 3226 | Swiss Federal Institute for Forest, Snow and Landscape Research (WSL) |
| 3227 | Wu, J., Larsen, K. S., van der Linden, L., Beier, C., Pilegaard, K. and A. Ibrom. 2013. Synthesis on the carbon budget and cycling in a Danish, temperate deciduous forest. Agricultural and Forest Meteorology. 181: 94-107. |
| 3228 | Zasada, J. C., and L. A. Viereck. 1970. White spruce cone and seed production in interior Alaska, 1957-68. Pacific Northwest Forest and Range Experiment Station. |
| 3229 | Zingg, A. and P. Brang. 2003. Sterben Buchen wegen der Trockenheit?. Wald und Holz. 9(03): 44-46. |
| 3230 | Zwander H., Klagenfurt; EAN-Datenbank Wien. Bearbeitung: R. Litschauer, Genetik, BFW-Wien |
| 3231 | Zwolak, R., Bogdziewicz, M. and L. Rychlik. 2016. Beech masting modifies the response of rodents to forest management. Forest Ecology and Management. 359: 268-276. |
| 4001 | unpublished, shared by co-author |
| 4002 | unpublished, shared by co-author |
| 4003 | Leeper, A. C., B. A. Lawrence, and J. M. LaMontagne. 2020. Plant-available soil nutrients have a limited influence on cone production patterns of individual white spruce trees. Oecologia 194:101-111. |
| 4004 | CEMP long term dataset, shared by Prof C. Krebs |
| 4005 | Godman, R. M., and G. A. Mattson. 1976. Seed crops and regeneration problems of 19 species in northeastern Wisconsin. |
| 4006 | LAMONTAGNE, J.M. and BOUTIN, S. 2007. Local-scale synchrony and variability in mast seed production patterns of Picea glauca. Journal of Ecology, 95: 991-1000. |
| 4007 | K. Van Cleve, F.S. Chapin, R.W. Ruess, and Bonanza Creek LTER. 2018. Bonanza Creek LTER: Yearly Seedfall Summary from 1985 to Present in the Bonanza Creek Experimental Forest near Fairbanks, Alaska ver 27. Environmental Data Initiative. https://doi.org/10.6073/pasta/9bad1efcad707ae23767ba2fadd2fea2 |
| 5001 | Abrahamson, W. G., and J. N. Layne. 2003. Long-term patterns of acorn production for five oak species in xeric Florida uplands. Ecology 84:2476-2492. |
| 5002 | Agren, J. 1988. BETWEEN-YEAR VARIATION IN FLOWERING AND FRUIT-SET IN FROST-PRONE AND FROST-SHELTERED POPULATIONS OF DIOECIOUS RUBUS-CHAMAEMORUS. Oecologia 76:175-183. |
| 5004 | Allen, R. B., N. W. H. Mason, S. J. Richardson, and K. H. Platt. 2012. Synchronicity, periodicity and bimodality in inter-annual tree seed production along an elevation gradient. Oikos 121:367-376. |
| 5005 | Ashton, D. H. 1975. STUDIES OF FLOWERING BEHAVIOR IN EUCALYPTUS-REGNANS F MUELL. Australian Journal of Botany 23:399-411. |
| 5008 | Barnes, B. V., Bingham, R. T., Schenk, J. A.. 2016. Insect caused loss to western white pine cones. USDA Forest Service Intermountain Forest and Range Experiment Station Research Note No. 102 |
| 5009 | Bate, C.G. 1930. The production, extraction, and germination of lodgepole pine seed. USDA Technical Bulletin No. 191. |
| 5010 | unpublished, shared by co-author |
| 5010 | Beck, D.E. Twelve-year acorn yield in southern Appalachian oaks. USDA Forest Service Research Note SE-244. |
| 5011 | unpublished, shared by co-author |
| 5012 | unpublished, shared by co-author |
| 5012 | Beveridge, A.E. 1973. Regeneration of podocarps in a central North Island forest. New Zealand Journal of Forestry 18, 23 (1973). |
| 5013 | unpublished, shared by co-author |
| 5013 | Brockie, R.E. 1986. Periodic heavy flowering of New Zealand flax (Phormium, Agavaceae). New Zealand Journal of Botany 24, 381 (1986). |
| 5014 | unpublished, shared by co-author |
| 5014 | Burns, K. C. 2012. Masting in a temperate tree: Evidence for environmental prediction? Austral Ecology 37:175-182. |
| 5015 | P. Y. Burns, D. M. Christisen, J. M. Nichols, Acorn production in the Missouri ozarks. University of Missouri Agricultural Experiment Station Bulletin 611, (1954). |
| 5016 | Burrows, L. E., and R. B. Allen. 1991. SILVER BEECH (NOTHOFAGUS-MENZIESII (HOOK F) OERST) SEEDFALL PATTERNS IN THE TAKITIMU RANGE, SOUTH-ISLAND, NEW-ZEALAND. New Zealand Journal of Botany 29:361-365. |
| 5017 | Cain, M. D., and M. G. Shelton. 2001. Secondary forest succession following reproduction cutting on the Upper Coastal Plain of southeastern Arkansas, USA. Forest Ecology and Management 146:223-238. |
| 5018 | G. D. Campbell, A study of jack pine seedfall on the Sandilands Forest Reserve, Manitoba. Government of Canada, Department of Forestry and Rural Development, Forest Research Laboratory, Winnipeg, Manitoba. Internal Report MS 71, 1 (1968). |
| 5019 | Canham, C. D., W. A. Ruscoe, E. F. Wright, and D. J. Wilson. 2014. Spatial and temporal variation in tree seed production and dispersal in a New Zealand temperate rainforest. Ecosphere 5:14. |
| 5020 | Caron, G. E., and G. R. Powell. 1989. PATTERNS OF SEED-CONE AND POLLEN-CONE PRODUCTION IN YOUNG PICEA-MARIANA TREES. Canadian Journal of Forest Research-Revue Canadienne De Recherche Forestiere 19:359-364. |
| 5021 | Cecich, R. A., and N. H. Sullivan. 1999. Influence of weather at time of pollination on acorn production of Quercus alba and Quercus velutina. Canadian Journal of Forest Research-Revue Canadienne De Recherche Forestiere 29:1817-1823. |
| 5023 | Clotfelter, E. D., A. B. Pedersen, J. A. Cranford, N. Ram, E. A. Snajdr, V. Nolan, and E. D. Ketterson. 2007. Acorn mast drives long-term dynamics of rodent and songbird populations. Oecologia 154:493-503. |
| 5024 | Cowan, P. E., and D. C. Waddington. 1990. SUPPRESSION OF FRUIT PRODUCTION OF THE ENDEMIC FOREST TREE, ELAEOCARPUS-DENTATUS, BY INTRODUCED MARSUPIAL BRUSHTAIL POSSUMS, TRICHOSURUS-VULPECULA. New Zealand Journal of Botany 28:217-224. |
| 5025 | Crone, E. E., and P. Lesica. 2004. Causes of synchronous flowering in Astragalus scaphoides, an iteroparous perennial plant. Ecology 85:1944-1954. |
| 5026 | Curran, L. M., and M. Leighton. 2000. Vertebrate responses to spatiotemporal variation in seed production of mast-fruiting dipterocarpaceae. Ecological Monographs 70:101-128. |
| 5027 | J. T. Curtis, The Vegetation of Wisconsin: An Ordination of Plant Communities. (University of Wisconsin Press, Madison, 1959). |
| 5028 | W. G. Dahms, J. W. Barrett, Seed production of central Oregon ponderosa and lodgepole pines. USDA Forest Service Research Paper PNW-191, (1975). |
| 5029 | R. Daubenmire, A seven-year study of cone production as related to xylem layers and temperature in Pinus ponderosa. American Midland Naturalist 64, 187 (1960). |
| 5030 | Davies, S. 1976. STUDIES OF FLOWERING SEASON AND FRUIT PRODUCTION OF SOME ARID ZONE SHRUBS AND TREES IN WESTERN AUSTRALIA. Journal of Ecology 64:665-687. |
| 5033 | Despland, E., and G. Houle. 1997. Climate influences on growth and reproduction of Pinus banksiana (Pinaceae) at the limit of the species distribution in eastern North America. American Journal of Botany 84:928-937. |
| 5035 | A. A. Downs, W. E. McQuilkin, Seed production of southern Appalachian oaks. Journal of Forestry 42, 913 (1944). |
| 5036 | Dunham, K. M. 1990. FRUIT PRODUCTION BY ACACIA-ALBIDA TREES IN ZAMBEZI RIVERINE WOODLANDS. Journal of Tropical Ecology 6:445-457. |
| 5037 | S. Eis, Association of western white pine cone crops with weather variables. Canadian Journal of Forest Research 6, 6 (1976). |
| 5038 | S. Eis, E. H. Garman, L. F. Ebell, Relation between cone production and diameter increment of Douglas fir (Pseudotsuga menziesii (Mirb.) Franco), grand fir (Abies grandis (Dougl.) Lindl.), and western white pine (Pinus monticola Dougl.). Canadian Journal of Botany 43, 1553 (1965). |
| 5038 | S. Eis, E. H. Garman, L. F. Ebell, Relation between cone production and diameter increment of Douglas fir (Pseudotsuga menziesii (Mirb.) Franco), grand fir (Abies grandis (Dougl.) Lindl.), and western white pine (Pinus monticola Dougl.). Canadian Journal of Botany 43, 1553 (1965). |
| 5039 | Elias, S. P., J. W. Witham, and M. L. Hunter. 2004. Peromyscus leucopus abundance and acorn mast: Population fluctuation patterns over 20 years. Journal of Mammalogy 85:743-747. |
| 5040 | Elkassaby, Y. A., and H. J. Barclay. 1992. COST OF REPRODUCTION IN DOUGLAS-FIR. Canadian Journal of Botany-Revue Canadienne De Botanique 70:1429-1432. |
| 5041 | Elmqvist, T., J. Agren, and A. Tunlid. 1988. SEXUAL DIMORPHISM AND BETWEEN-YEAR VARIATION IN FLOWERING, FRUIT-SET AND POLLINATOR BEHAVIOR IN A BOREAL WILLOW. Oikos 53:58-66. |
| 5042 | Enright, N. J. 1992. FACTORS AFFECTING REPRODUCTIVE-BEHAVIOR IN THE NEW-ZEALAND NIKAU PALM, RHOPALOSTYLIS-SAPIDA WENDL-ET-DRUDE. New Zealand Journal of Botany 30:69-80. |
| 5043 | Espelta, J. M., P. Cortes, R. Molowny-Horas, B. Sanchez-Humanes, and J. Retana. 2008. Masting mediated by summer drought reduces acorn predation in mediterranean oak forests. Ecology 89:805-817. |
| 5044 | Flowerdew, J. R., and G. Gardner. 1978. SMALL RODENT POPULATIONS AND FOOD-SUPPLY IN A DERBYSHIRE ASHWOOD. Journal of Animal Ecology 47:725-740. |
| 5045 | Forcella, F. 1981. ESTIMATING PINYON CONE PRODUCTION IN NEW-MEXICO AND WESTERN OKLAHOMA. Journal of Wildlife Management 45:553-557. |
| 5046 | H. A. Fowells, G. H. Schubert, Seed crops of forest trees in the pine region of California. USDA Technical Bulletin 1150, (1956). |
| 5047 | J. F. Franklin, Cone production by upper slope conifers. Pacific NW Forest Range Experiment Station Research Paper No. PNW-60, (1968). |
| 5048 | Garcia-Mozo, H., E. Dominguez-Vilches, and C. Galan. 2012. A model to account for variations in holm-oak (Quercus ilex subsp ballota) acorn production in southern Spain. Annals of Agricultural and Environmental Medicine 19:403-408. |
| 5049 | J. S. Gashwiler, Seed fall of three conifers in west-central Oregon. Forest Science 15, 290 (1969). |
| 5050 | P. D. Goodrum, V. H. Reid, C. E. Boyd, Acorn yields, characteristics, and management criteria of oaks for wildlife. Journal of Wildlife Management 35, 520 (1971). |
| 5051 | R. E. Graber, W. B. Leak, Seed Fall in an old-growth northern hardwood forest. USDA Forest Service Northeastern Forest Experiment Station Research Paper NE-663, 1 (1992). |
| 5052 | Greenberg, C. H. 2000. Individual variation in acorn production by five species of southern Appalachian oaks. Forest Ecology and Management 132:199-210. |
| 5053 | Greenberg, C. H., D. J. Levey, C. Kwit, J. P. McCarty, S. F. Pearson, S. Sargent, and J. Kilgo. 2012. Long-Term Patterns of Fruit Production in Five Forest Types of the South Carolina Upper Coastal Plain. Journal of Wildlife Management 76:1036-1046. |
| 5054 | unpublished data |
| 5055 | Guitian, J., P. Guitian, and L. Navarro. 1996. Fruit set, fruit reduction, and fruiting strategy in Cornus sanguinea (Cornaceae). American Journal of Botany 83:744-748. |
| 5056 | Guitian, J., and T. Bermejo. 2006. Dynamics of plant-frugivore interactions: a long-term perspective on holly-redwing relationships in northern Spain. Acta Oecologica-International Journal of Ecology 30:151-160. |
| 5057 | Gurnell, J., P. W. W. Lurz, M. D. F. Shirley, S. Cartmel, P. J. Garson, L. Magris, and J. Steele. 2004. Monitoring red squirrels Sciurus vulgaris and grey squirrels Sciurus carolinensis in Britain. Mammal Review 34:51-74. |
| 5058 | L. W. Gysel, W. A. Lemmien, An eight-year record of fruit production. Journal of Wildlife Management 28, 175 (1964). |
| 5059 | L. W. Gysel, Acorn production on good, medium, and poor oak sites in southern Michigan. Journal of Forestry 55, 570 (1957). |
| 5060 | L. W. Gysel, A 10-year analysis of beechnut production and use in Michigan. Journal of Wildlife Management 35, 516 (1971). |
| 5061 | S. Hagner, Cone crop fluctuations in Scots pine and Norway spruce. Studia Forestalia Suecica 22, 1 (1965). |
| 5062 | Healy, W. M., A. M. Lewis, and E. F. Boose. 1999. Variation of red oak acorn production. Forest Ecology and Management 116:1-11. |
| 5063 | A. F. Hedlin, A six-year plot study on Douglas-fir cone insect population fluctuations. Forest Science 10, 124 (1964). |
| 5064 | Tonini H and Pedrozo CA (2014). Variações anuais na produção de frutos e sementes de castanheira-do-brasil (Bertholletia |
| 5065 | C. M. Herrera, Long-term dynamics of Mediterranean frugivorous birds and fleshy fruits: a 12-year study. Ecological Monographs 68, 511 (1998). |
| 5066 | J. E. Hickey, G. R. Wilkinson, Long-term regeneration trends from a silvicultural systems trial in lowland cool temperate rainforest in Tasmania. Tasforests 11, 1 (1999). |
| 5068 | Hiroki, S., and T. Matsubara. 1995. FLUCTUATION OF NUT PRODUCTION AND SEEDLING APPEARANCE OF A JAPANESE BEECH (FAGUS-CRENATA BLUME). Ecological Research 10:161-169. |
| 5069 | G. Hoch, R. T. W. Siegwolf, S. G. Keel, C. Körner, Q. Han, Fruit production in three masting tree species does not rely on stored carbon reserves. Oecologia 171, 653 (2013). |
| 5070 | Hofgaard, A. 1993. SEED RAIN QUANTITY AND QUALITY, 1984-1992, IN A HIGH-ALTITUDE OLD-GROWTH SPRUCE FOREST, NORTHERN SWEDEN. New Phytologist 125:635-640. |
| 5071 | Van Cleve, Keith; Chapin, F Stuart; Ruess, Roger. 2021. Bonanza Creek LTER: Yearly Seedfall Summary from 1957 to Present in the Bonanza Creek Experimental Forest near Fairbanks, Alaska, Bonanza Creek LTER - University of Alaska Fairbanks. BNZ:14, http://www.lter.uaf.edu/data/data-detail/id/14. doi:10.6073/pasta/8ecb7a0eca30148914bcf9cc1472d327 |
| 5072 | S.-V. Holm, Reproductive patterns of Betula pendula and B. pubescens coll. along a regional altitudinal gradient in northern Sweden. Ecography 17, 60 (1994). |
| 5073 | Houle, G., and L. Filion. 1993. INTERANNUAL VARIATIONS IN THE SEED PRODUCTION OF PINUS-BANKSIANA AT THE LIMIT OF THE SPECIES DISTRIBUTION IN NORTHERN QUEBEC, CANADA. American Journal of Botany 80:1242-1250. |
| 5074 | Houle, G. 1999. Mast seeding in Abies balsamea, Acer saccharum and Betula alleghaniensis in an old growth, cold temperate forest of north-eastern North America. Journal of Ecology 87:413-422. |
| 5075 | H. F. Howe, Consequences of seed dispersal by birds: a case study from Central America. Journal of the Bombay Natural History Society 83, 19 (1986). |
| 5077 | M. Imada, T. Nakai, T. Nakamura, T. Mabuchi, Y. Takahashi, Acorn dispersal in natural stands of mizunara (Quercus mongolica var. grosseserrata) for twenty years. Journal of the Japanese Forestry Society 72, 426 (1990). |
| 5078 | I. L. James, D. A. Franklin, Recruitment, growth and survival of rimu seedlings in selectively logged terrace rimu forest. New Zealand Journal of Forestry Science 8, 207 (1978). |
| 5079 | D. H. Janzen, in Tropical Trees as Living Systems, P. B. Tomlinson, M. H. Zimmerman, Eds. (Cambridge University Press, New York, 1978), pp. 83-128. |
| 5080 | D. H. Janzen, in Advances in Legume Biology, C. H. Stirton, J. L. Zarucchi, Eds. (Missouri Botanical Garden, St. Louis, 1989), pp. 293-376. |
| 5081 | P. G. Jensen, C. L. Demers, S. A. McNulty, W. J. Jakubas, M. M. Humphries, Marten and fisher responses to fluctuations in prey populations and mast crops in the northern hardwood forest. Journal of Wildlife Management 76, 489 (2012). |
| 5083 | P. Jordano, Geographical ecology and variation of plant-seed disperser interactions: southern Spanish junipers and frugivorous thrushes. Vegetatio 107/108, 85 (1993). |
| 5084 | 226–250). Oxford University Press. |
| 5085 | K. A. Kainer, L. H. O. Wadt, C. L. Staudhammer, Explaining variation in Brazil nut fruit production. Forest Ecology and Management 250, 244 (2007). |
| 5086 | H. Kang, A five-year study of mast seeding in Pinus densiflora. Journal of Plant Biology 48, 159 (2005). |
| 5087 | T. N. Kaye, “Population monitoring and preliminary viability model of Snake River goldenweed, Haplopappus radiatus: tenth year progress report” (Bureau of Land Management Vale District and Institute for Applied Ecology, Corvalis, OR, 2001). |
| 5088 | Kelly, D., A. L. Harrison, W. G. Lee, I. J. Payton, P. R. Wilson, and E. M. Schauber. 2000. Predator satiation and extreme mast seeding in 11 species of Chionochloa (Poaceae). Oikos 90:477-488. |
| 5088 | Kelly, D., M. H. Turnbull, R. P. Pharis, and M. S. Sarfati. 2008. Mast seeding, predator satiation, and temperature cues in Chionochloa (Poaceae). Population Ecology 50:343-355. |
| 5089 | K. S. Kim, H. M. Kwon, S. Y. Shim, Y. J. Kim, Effects of tree age and meteorological factors on the seed production of Larix leptolepis Gordon. Research Report Institute Forestry General Korea 25, 41 (1989). |
| 5090 | C. M. King, The relationships between beech (Nothofagus Sp.) seedfall and populations of mice (Mus musculus), and the demographic and dietary responses of stoats (Mustela erminea), in three New Zealand forests. Journal of Animal Ecology 52, 141 (1983). |
| 5092 | Koenig, W. D., and J. M. H. Knops. 2013. Large-scale spatial synchrony and cross-synchrony in acorn production by two California oaks. Ecology 94:83-93. |
| 5093 | unpublished, shared by co-author |
| 5095 | Kraft, K. J. 1968. ECOLOGY OF CONE MOTH LASPEYRESIA TOREUTA IN PINUS BANKSIANA STANDS. Annals of the Entomological Society of America 61:1462-&. |
| 5096 | CEMP long term dataset, shared by Prof C. Krebs |
| 5099 | M. M. Larson, G. H. Shubert, Cone crops of ponderosa pine in central Arizona, including the influence of Abert squirrels. USDA Forest Service Research Paper RM-58, (1970). |
| 5100 | Layne, J. N., and W. G. Abrahamson. 2004. Long-term trends in annual reproductive output of the scrub hickory: Factors influencing variation in size of nut crop. American Journal of Botany 91:1378-1386. |
| 5101 | W. B. Leak, R. E. Graber, Six-year beechnut production in New Hampshire. Forest Service Northeastern Forest Experiment Station Research Paper NE-677, (1993). |
| 5102 | Lester, D. T. 1967. VARIATION IN CONE PRODUCTION OF RED PINE IN RELATION TO WEATHER. Canadian Journal of Botany 45:1683-&. |
| 5103 | Lobo, N., and J. S. Millar. 2013. Indirect and mitigated effects of pulsed resources on the population dynamics of a northern rodent. Journal of Animal Ecology 82:814-825. |
| 5104 | Maeto, K., and K. Ozaki. 2003. Prolonged diapause of specialist seed-feeders makes predator satiation unstable in masting of Quercus crispula. Oecologia 137:392-398. |
| 5106 | M. Martínez-Ramos, J. Sarukhán, D. Piñero, in Plant Population Ecology, A. J. Davy, M. J. Hutchings, A. R. Watkinson, Eds. (Blackwell Scientific Publications, Oxford, 1988), pp. 293-313. |
| 5107 | A. Martín Vicente, J. M. Infante, J. García Gordo, J. Merino, R. Fernández Alés, Producción de bellotas en montes y dehesas del suoeste Español. Pastos 28, 237 (1998). |
| 5109 | Masaka, K., and H. Sato. 2002. Acorn production by Kashiwa oak in a coastal forest under fluctuating weather conditions. Canadian Journal of Forest Research-Revue Canadienne De Recherche Forestiere 32:9-15. |
| 5110 | Masaki, T., T. Oka, K. Osumi, and W. Suzuki. 2008. Geographical variation in climatic cues for mast seeding of Fagus crenata. Population Ecology 50:357-366. |
| 5111 | Mattson, W. J. 1971. RELATIONSHIP BETWEEN CONE CROP SIZE AND CONE DAMAGE BY INSECTS IN RED PINE SEED-PRODUCTION AREAS. Canadian Entomologist 103:617-&. |
| 5112 | B. F. McLemore, Cone and seed characteristics of fertilized and unfertilized longleaf pines. USDA Forest Service Research Paper SO-109, (1975). |
| 5113 | W. M. McNeill, Observatoins on cone and seed production in plantations of scots pine in Scotland. Forestry 27, 122 (1954). |
| 5114 | McQuilkin, R. A., and R. A. Musbach. 1977. PIN OAK ACORN PRODUCTION ON GREEN TREE RESERVOIRS IN SOUTHEASTERN MISSOURI. Journal of Wildlife Management 41:218-225. |
| 5115 | McShea, W. J. 2000. The influence of acorn crops on annual variation in rodent and bird populations. Ecology 81:228-238. |
| 5118 | Mooney, K. A., Y. B. Linhart, and M. A. Snyder. 2011. Masting in ponderosa pine: comparisons of pollen and seed over space and time. Oecologia 165:651-661. |
| 5120 | Newbery, D. M., G. B. Chuyong, and L. Zimmermann. 2006. Mast fruiting of large ectomycorrhizal African rain forest trees: importance of dry season intensity, and the resource-limitation hypothesis. New Phytologist 170:561-579. |
| 5121 | M. G. Neyland, L. G. Edwards, N. J. Kelly, Seedfall of Eucalyptus obliqua at two sites within the Forestier silvicultural systems trial, Tasmania. Tasforests 14, 23 (2003). |
| 5122 | A. B. Nielsen et al., The effect of climate conditions on inter-annual flowering variability monitored by pollen traps below the canopy in Draved Forest, Denmark. Vegetation History and Archaeobotany 19, 309 (2010). |
| 5123 | Nixon, C. M., M. W. McClain, and R. W. Donohoe. 1975. EFFECTS OF HUNTING AND MAST CROPS ON A SQUIRREL POPULATION. Journal of Wildlife Management 39:1-25. |
| 5123 | C. M. Nixon, M. W. McClain, R. W. Donohoe, Effects of hunting and mast crops on a squirrel population. Journal of Wildlife Management 39, 1 (1975). |
| 5124 | D. L. Noble, F. Ronco Jr., Seedfall and establishment of Engelmann spruce and subalpine fir in clearcut openings in Colorado. USDA Forest Service Research Paper RM-200, (1978). |
| 5125 | Norghauer, J. M., C. A. Nock, and J. Grogan. 2011. The Importance of Tree Size and Fecundity for Wind Dispersal of Big-Leaf Mahogany. Plos One 6:12. |
| 5126 | Norton, D. A., and D. Kelly. 1988. Mast seeding over 33 years by Dacrydium cupressinum Lamb. (rimu) (Podocarpaceae) in New Zealand: the importance of economies of scale. Functional Ecology 2:399-408. |
| 5127 | W. H. Olson, D. E. Ramos, R. G. Snyder, Alternate-year walnut pruning can boost yields, cut costs. California Agriculture 48, 20 (1994). |
| 5128 | Ostfeld, R. S., C. D. Canham, K. Oggenfuss, R. J. Winchcombe, and F. Keesing. 2006. Climate, deer, rodents, and acorns as determinants of variation in Lyme-disease risk. Plos Biology 4:1058-1068. |
| 5129 | Rosenberyy, C.S., Fleegle, J.T., Wallingford, B. D. 2009. MANAGEMENT AND BIOLOGY OF WHITE-TAILED DEER IN PENNSYLVANIA 2009-2018. Deer and Elk Section Bureau of Wildlife Management, Pennsylvania Game Commission |
| 5130 | Perez-Ramos, I. M., J. M. Ourcival, J. M. Limousin, and S. Rambal. 2010. Mast seeding under increasing drought: results from a long-term data set and from a rainfall exclusion experiment. Ecology 91:3057-3068. |
| 5131 | K. B. Pomeroy, C. F. Korstian, Further results on loblolly pine seed production and dispersal. Journal of Forestry 47, 968 (1949). |
| 5132 | Pons, J., and J. G. Pausas. 2012. The coexistence of acorns with different maturation patterns explains acorn production variability in cork oak. Oecologia 169:723-731. |
| 5133 | M. B. Presendorfer et al., Stand density and acorn production of the island scrub-oak (Quercus pacifica). Western North American Naturalist, (2015). |
| 5134 | Pucek, Z., W. Jedrzejewski, B. Jedrzejewska, and M. Pucek. 1993. RODENT POPULATION-DYNAMICS IN A PRIMEVAL DECIDUOUS FOREST (BIALOWIEZA-NATIONAL-PARK) IN RELATION TO WEATHER, SEED CROP, AND PREDATION. Acta Theriologica 38:199-232. |
| 5135 | G. E. Rehfeldt, A. R. Stage, R. T. Bingham, Strobili development in western white pine: periodicity, prediction, and association with weather. Forest Science 17, 454 (1971). |
| 5136 | Reukema, D. L. 1982. SEEDFALL IN A YOUNG-GROWTH DOUGLAS-FIR STAND - 1950-1978. Canadian Journal of Forest Research-Revue Canadienne De Recherche Forestiere 12:249-254. |
| 5137 | Roland, C. A., J. H. Schmidt, and J. F. Johnstone. 2014a. Climate sensitivity of reproduction in a mast-seeding boreal conifer across its distributional range from lowland to treeline forests. Oecologia 174:665-677. |
| 5140 | Rossi, S., H. Morin, D. Laprise, and F. Gionest. 2012. Testing masting mechanisms of boreal forest species at different stand densities. Oikos 121:665-674. |
| 5141 | D. F. Roy, Douglas-fir seed dispersal in northwestern California. Pacific SW Forest and Range Experiment Station Technical Paper No. 49, (1960). |
| 5142 | H. Saito, M. Takeoka, Pollen production rates in a young Japanese red pine forest. Japanese Journal of Ecology 35, 67 (1985). |
| 5143 | H. Saito, T. Itsubo, M. Takeoka, Production rates of reproductive organ in Quercus serrata stands: the investment of photosynthates in seed production. Bulletin Kyoto Prefectural University, Forestry 35, 1 (1991). |
| 5144 | H. Saito, H. Imai, M. Takeoka, Peculiarities of sexual reproduction in Fagus crenata forests in relation to annual production of reproductive organs. Ecological Research 6, 277 (1991). |
| 5145 | Saito, H., H. Imai, and M. Takeoka. 1991. PECULIARITIES OF SEXUAL REPRODUCTION IN FAGUS-CRENATA FORESTS IN RELATION TO ANNUAL PRODUCTION OF REPRODUCTIVE-ORGANS. Ecological Research 6:277-290. |
| 5146 | H. Saito et al., A comparison of different ages for the male flower, pollen, female flower and seed production of Quercus mongolica var. grosseserata stands. Scientific. Scientific Reorts of the Kyoto Prefectural University, Agriculture 41, 46 (1989). |
| 5147 | H. Saito, H. Imai, M. Takeoka, Peculiarities of sexual reproduction in Fagus crenata forests in relation to annual production of reproductive organs. Ecological Research 6, 277 (1991). |
| 5148 | Sanguinetti, J., and T. Kitzberger. 2008. Patterns and mechanisms of masting in the large‐seeded southern hemisphere conifer Araucaria araucana. Austral Ecology 33:78-87. |
| 5150 | Schauber, E. M., D. Kelly, P. Turchin, C. Simon, W. G. Lee, R. B. Allen, I. J. Payton, P. R. Wilson, P. E. Cowan, and R. E. Brockie. 2002. Masting by eighteen New Zealand plant species: The role of temperature as a synchronizing cue. Ecology 83:1214-1225. |
| 5151 | Schulz, K., and J. Zasada. 2004. Annual, local, and individual variation in the inflorescence and fruit production of eastern leatherwood (Dirca palustris L. Thymelaeaceae). Journal of the Torrey Botanical Society 131:292-304. |
| 5152 | Seki, T. 2008. Influence of annually fluctuating seed-cone production and climatic factors on the upper-crown expansion of canopy trees of Abies mariesii Masters (Pinaceae). Plant Species Biology 23:129-139. |
| 5153 | Shearer, R. C., and W. C. Schmidt. 1971. PONDEROSA PINE CONE AND SEED LOSSES. Journal of Forestry 69:370-&. |
| 5154 | R. C. Shearer, Western larch seed dispersal over clear-cut blocks in northwestern Montana. Proceedings of the Montana Academy of Sciences 19, 130 (1960). |
| 5155 | Shepperd, W. D., C. B. Edminster, and S. A. Mata. 2006. Long-term seedfall, establishment, survival, and growth of natural and planted ponderosa pine in the Colorado front range. Western Journal of Applied Forestry 21:19-26. |
| 5156 | Simon, B. E., F. Latorre, and C. Rotundo. 2018. Study of the reproductive phenology of Araucaria angustifolia in two environments of Argentina: Its application to the management of a species at risk. Global Ecology and Conservation 16:12. |
| 5158 | Sork, V. L., J. Bramble, and O. Sexton. 1993. ECOLOGY OF MAST-FRUITING IN 3 SPECIES OF NORTH-AMERICAN DECIDUOUS OAKS. Ecology 74:528-541. |
| 5159 | Souza, A. F., D. U. de Matos, C. Forgiarini, and J. Martinez. 2010. Seed crop size variation in the dominant South American conifer Araucaria angustifolia. Acta Oecologica-International Journal of Ecology 36:126-134. |
| 5160 | Sperens, U. 1997. Fruit production in Sorbus aucuparia L (Rosaceae) and pre-dispersal seed predation by the apple fruit moth (Argyresthia conjugella Zell). Oecologia 110:368-373. |
| 5161 | Summers, R. W. 2011. Patterns of exploitation of annually varying Pinus sylvestris cone crops by seed-eaters of differing dispersal ability. Ecography 34:723-728. |
| 5162 | Sundahl, W. E. 1971. SEEDFALL FROM YOUNG - GROWTH PONDEROSA PINE. Journal of Forestry 69:790-&. |
| 5163 | Suzuki, W., K. Osumi, and T. Masaki. 2005. Mast seeding and its spatial scale in Fagus crenata in northern Japan. Forest Ecology and Management 205:105-116. |
| 5164 | K. Tallqvist, Results of long-time measurements of the quality of flowering and seed crop of trees. Folia Forestali 364`, 1 (1978). |
| 5165 | Tanaka, H. 1995. Seed demography of three co-occurring Acer species in a Japanese temperate deciduous forest. Journal of Vegetation Science 6:887-896. |
| 5165 | Tapper, P. G. 1992. IRREGULAR FRUITING IN FRAXINUS-EXCELSIOR. Journal of Vegetation Science 3:41-46. |
| 5165 | Tapper, P. G. 1996. Long-term patterns of mast fruiting in Fraxinus excelsior. Ecology 77:2567-2572. |
| 5166 | E. H. Tryon, K. L. Carvell, Acorn production and damage. West Virginia University Agricultural Experiment Station Bulletin 466T, (1962). |
| 5167 | Tsuji, Y., S. Fujita, H. Sugiura, C. Saito, and S. Takatsuki. 2006. Long-term variation in fruiting and the food habits of wild Japanese macaques on Kinkazan Island, northern Japan. American Journal of Primatology 68:1068-1080. |
| 5167 | Tsuji, Y., and S. Takatsuki. 2009. Effects of Yearly Change in Nut Fruiting on Autumn Home-range Use by Macaca fuscata on Kinkazan Island, Northern Japan. International Journal of Primatology 30:169-181. |
| 5168 | R. M. Waldron, Cone production and seedfall in a mature white spruce stand. Forestry Chronicle 41, 316 (1965). |
| 5169 | J. A. Wardle, The New Zealand beeches: ecology, utilization and management. (New Zealand Forest Service, Christchurch, New Zealand, 1984). |
| 5170 | D. A. Way et al., Greater seed production in elevated CO2 is not accompanied by reduced seed quality in Pinus taeda L. Global Change Biology 16, 1046 (2010). |
| 5171 | M. J. Weaver, F. Forcella, in Proceedings of a symposium on conifer tree seeds in the Inland Mountain west. (USDA Forest Service General Technical Report INT-203, 1986), pp. 68-76. |
| 5172 | K. F. Wenger, Annual variation in the seed crops of loblolly pine. Journal of Forestry 55, 567 (1957). |
| 5175 | Wilson, P. R., B. J. Karl, R. J. Toft, J. R. Beggs, and R. H. Taylor. 1998. The role of introduced predators and competitors in the decline of kaka (Nestor meridionalis) populations in New Zealand. Biological Conservation 83:175-185. |
| 5176 | O. M. Wood, A brief record of seed productivity for chestnut oak in southern New Jersey. Journal of Forestry 32, 1014 (1934). |
| 5177 | Woodward, A., D. G. Silsbee, E. G. Schreiner, and J. E. Means. 1994. INFLUENCE OF CLIMATE ON RADIAL GROWTH AND CONE PRODUCTION IN SUB-ALPINE FIR (ABIES-LASIOCARPA) AND MOUNTAIN HEMLOCK (TSUGA-MERTENSIANA). Canadian Journal of Forest Research-Revue Canadienne De Recherche Forestiere 24:1133-1143. |
| 5178 | Wright, S. J., H. C. Muller-Landau, O. Calderon, and A. Hernandez. 2005. Annual and spatial variation in seedfall and seedling recruitment in a neotropical forest. Ecology 86:848-860. |
| 5179 | Yasaka, M., M. Takiya, I. Watanabe, Y. Oono, and N. Mizui. 2008. Variation in seed production among years and among individuals in 11 broadleaf tree species in northern Japan. Journal of Forest Research 13:83-88. |
| 5180 | R. I. Zlotin, R. R. Parmenter, Patterns of mast production in pinyon and juniper woodlands along a precipitation gradient in central New Mexico (Sevilleta National Wildlife Refuge). Journal of Arid Environments 72, 1562 (2008). |
| 6001 | Department of Forest Genetics, Federal Research and Training Centre for Forests, Natural Hazards and Landscape (BFW). |
| 6002 | unpublished, shared by co-author |
| 6003 | unpublished, shared by co-author |
| 6004 | unpublished, shared by co-author |
| 6005 | unpublished, shared by co-author |
| 6006 | Bogdziewicz, M., Fernández-Martínez, M., Espelta, J.M., Ogaya, R. and Penuelas, J. (2020), Is forest fecundity resistant to drought? Results from an 18-yr rainfall-reduction experiment. New Phytol, 227: 1073-1080. https://doi.org/10.1111/nph.16597 |
| 6007 | Bogdziewicz, M., Pesendorfer, M., Crone, E.E., Pérez-Izquierdo, C. and Bonal, R. (2020), Flowering synchrony drives reproductive success in a wind-pollinated tree. Ecol. Lett., 23: 1820-1826. https://doi.org/10.1111/ele.13609 |
| 6008 | M. Żywiec, M. Ledwoń, J. Holeksa, P. Seget, B. Łopata and J. M. Fedriani. 2018. Rare events of massive plant reproductive investment lead to long‐term density‐dependent reproductive success. Journal of Ecology, 106, 1307-1318 |
| 6009 | unpublished, shared by co-author |
| 6181 | RENECOFOR, shared by co-authors |
| 6182 | unpublished, shared by co-author |
| 6183 | unpublished, shared by co-author (Christie Klimas). Klimas, C.A., Wadt, L.H.D., de Castilho, C.V., Lira-Guedes, A.C., da Costa, P., da Fonseca, F.L., 2021. Variation in Seed Harvest Potential of *Carapa guianensis* Aublet in the Brazilian Amazon: A Multi-Year, Multi-Region Study of Determinants of Mast Seeding and Seed Quantity. Forests 12, 1-20. |
| 6184 | unpublished, shared by co-author |
| 6185 | upublished, shared by co-author (Felipe Carevic) |
| 6186 | upublished, shared by co-author (Justin DeRose) |
| 6187 | https://doi.org/10.5061/dryad.4qrfj6q9m |
| 6188 | https://doi.org/10.5061/dryad.1s625 |
| 6189 | https://doi.org/10.5061/dryad.v6wwpzgrb |
| 6190 | https://doi.org/10.5061/dryad.772g3 |
| 6191 | https://doi.org/10.5061/dryad.pv608 |
| 6192 | https://doi.org/10.5061/dryad.stqjq2c0c |
| 6193 | https://doi.org/10.5061/dryad.61m318c |
| 6194 | https://doi.org/10.5061/dryad.75v7c |
| 6195 | Tutin, CEG; Abernethy, K; White, L; Dimoto, E; Dikangadissi, JT; Jeffery, KJ; Momont, L; Ukizintambara, T; Bush, ER (2029): Lopé Tree Phenology Dataset. Version 1.2. University of Stirling. Faculty of Natural Sciences. Dataset. http://hdl.handle.net/11667/152 |
| 6196 | unpublished, shared by co-authors Urs Kalbitzer and Colin Chapman |
| 6197 | unpublished, shared by co-authors Georg Gratzer and Mario Pesendorfer |
