# Supplementary material for: MASTREE+: Time‐series of plant reproductive effort from six continents
Source: Glob Chang Biol. 2022 Mar 5;28(9):3066–82. doi: 10.1111/gcb.16130 (PMC9314730; doi:10.1111/gcb.16130)
Supplement: Supplementary file 5 — Appendix S5 [file GCB-28-3066-s005.docx]

Appendix 5: Extended description of database variables

| **Variable**  **(Column header)** | **Definition** | **Data format** | **Description** | **Possible values** |
| --- | --- | --- | --- | --- |
| Alpha_Number | Unique code associated with each source of data. | Numeric, 4 characters. | An Alpha_Number is associated with each unique original source of data, and links to the reference list in Appendix 4. |  |
| Segment | Code to indicate a temporal segment of a time-series containing gaps. | Numeric, 2 characters. | Years with no observations are not recorded. Consequently, time-series that would otherwise contain gaps are indicated by sequential segments codes. Individual time-series can consist of multiple segments.  For example, the data from Kaye et al (2001) (Alpha_Number = 5087) includes a single time-series measured 1991-2000, but with no data in 1993. Consequently, observations (rows in the .csv file) corresponding to the years 1991-1992 have a Segment code = 1, and those for the years 1994-2000 have Segment code = 2.  The Segment variable can be used to split time-series when continuous series without data gaps are required.  Note that the Start, End and Length variables refer to time-series, not segments (see below). |  |
| Site_number | Code to differentiate multiple sites from the same original source. | Numeric, 3 characters | Where a single study (Study_ID/Alpha_number) contains data from multiple sample sites, the Site_number is used to differentiate sites. This will correspond to unique site names under the variable “Site”, but a numeric code is included to facilitate the creation of unique time-series IDs that correspond to unique of combinations of Alpha_number, Site_number, Variable_number and Species_code (and, optionally, “Segment” if unique time-series must not contain missing years of observations, see “Segment”). |  |
| Variable_number | Code to differentiate multiple measures of reproductive output from the same species-site combination. | Numeric, 1 character | In a small number of cases, reproductive effort is available for multiple measures or reproductive stages. For example, Boulanger-Lapointe et al (2017) (Alpha_number = 0187) includes data on annual flower and fruit production for *Vaccinium myrtillus* from the same study sites. These separate records are indicated by differing values for the variables “Unit”, “Variable” and/or “Collection_method”, but it is also coded under “Variable_number” to enable users to easily check for potential pseudo-replication of time-series. |  |
| Year | Year of observation. | Numeric, 4 characters | The calendar year associated with the observation, as indicated in the original record. Where species have a clearly defined phenological season for flowering or fruit/seed maturation (i.e., in the extratropics), this corresponds to the calendar year at the time of flowering or fruit/seed maturation as indicated in the original record.  In the tropics, or other instances where flowering/fruiting may occur throughout the year, annual production is summed across a 12-month period. By default, this corresponds to the calendar year, but in many instances a more biologically meaningful 12-month window is used. Where the seasonal sum was calculated by the original data collectors or authors, we used this. Otherwise, we consulted the collectors of the data, or the literature, to determine the optimal 12-month window. This information can be found under “Comments”. Where the 12-month window does not run January-December, we date the “Year” according to calendar year of the month the start of the window. |  |
| Species | Species identifier. | Alphanumeric, variable number of characters | Species identifiers are standardised according to The Plant List nomenclature.  “spp.” is used to indicate a record identified to the genus level only, e.g., Quercus spp. refers to a record where reproduction is only identified to the genus level.  “MIXED” indicates a non-species-specific community-level estimate of annual reproductive effort. |  |
| Species_code | Six-character species identifier. | Alphanumeric, 6 characters | A unique six-character code for each species. Assigned by automatically combining the first three characters from The Plant List-standardised genus and species names. Where separate species shared a Species_code, a unique combination was manually created.  For populations of a hybrid origin, the final character was changed to “X”.  SPP indicates a record only identified to the genus level, e.g., QUESPP (see “Species”).  MIXSPP indicates a non-species-specific community-level record of annual reproductive effort. |  |
| Mono_Poly | Monocarpic or polycarpic species. | Alphanumeric, 1 character | A code indicated whether the species is monocarpic (semelparous) or polycarpic (iteroparous). | M, P |
| Value | The measured value of annual reproductive output. | Numeric, variable number of characters | The measured value of annual reproductive output. |  |
| VarType | The variable type: Continuous or Ordinal. | Alphanumeric, 1 character | The dataset contains continuous and ordinal scale time-series. Continuous time-series are recorded on a continuous scale. Ordinal series are recorded on an ordered categorical scale. All ordinal series were rescaled to start at 1 (lowest reproductive effort) and to contain only integer values. For example, an ordinal series with reported values of 0, 1, 2, 3, 4 (5 levels in total) is rescaled to 1, 2, 3, 4, 5. | C, O |
| Unit | The unit of measurement, where VarType is continuous. | Alphanumeric and special characters, variable number of characters | The unit of measurement for the record. For continuous series, we converted data into a common unit where possible (e.g., we converted “seeds/ha” to “seeds/m^2^”).  “index” is used where reproductive effort is quantified on a unitless scale (e.g., standardised time-series).  Ordinal series are unitless, and are indicated by “NA”. |  |
| Max_Value | The maximum value in a time-series. | Numeric, variable number of characters | The maximum observed Value within the time-series (incorporating all Segments). |  |
| Variable | Categorical classification of the measured variable. | Alphanumeric, variable number of characters | A classification of the measured variable used to assess reproductive effort for the record.  Total reproductive organs refer to datasets where all reproductive organs were measured – e.g. the combined mass of flowers and fruits were reported. | cone, flower, fruit, seed, pollen, total reproduction organs |
| Collection_method | Classification of the method used to measure reproductive effort. | Alphanumeric, variable number of characters | A classification of the collection method used to measure reproductive effort for the record. If users require specific information on the collection methodology for individual sites, the original sources should be consulted.  Seed trap: estimates of reproductive effort based on trapping of reproductive organs in seed traps (litter traps). May include estimates based on counts or mass.  Cone count, flower count, fruit count, seed count: estimates based on counts of the specified reproductive organs either on the plant, or from the ground after dispersal.  Cone scar count, fruit scar count: estimates based on the counting of absolutely dated characteristic scars on branches or stems left by fruits or cones.  Harvest record: estimates based on fruit or seed harvest records, usually from a standardised collection area (often regional).  Visual crop assessment: estimates of reproductive effort based on a visual estimate of crop size. Usually associated with ordinal time-series, or continuous time-series where the unit is “% individs reproducing”.  Pollen count, lake sediment pollen count: estimates based on a method for counting pollen grains.  Dendrochronological reconstruction: estimates of reproductive effort based on tree-ring methodologies.    Other quantification: other methods for estimating reproductive effort. Further details may be included in “Comments”. Also used where details of the collection method are not provided, particularly in the oldest records (e.g., forestry records). | cone count, cone scar count, flower count, fruit count, fruit scar count, seed count, seed trap, pollen count, lake sediment pollen count, harvest record, visual crop assessment,  other quantification, dendrochronological reconstruction |
| Latitude | Latitude of the record. | Numeric, variable number of characters | Latitude of the sample location for the record, in decimal degrees. |  |
| Longitude | Longitude of the record. | Numeric, variable number of characters | Latitude of the sample location for the record, in decimal degrees. |  |
| Coordinate_flag | A flag to indicate the precision of the latitude and longitude. | Alphanumeric, 1 character | An indication of the precision of the latitude and longitude associated with the record. “A” and “B” can be considered to represent location information of high precision, but “C” might involve lower precision. Where high precision location information is necessary, records with Coordinate_flag = C might be excluded.  A = coordinates provided in the original source  B = coordinates estimated by the compiler based on a map or other location information provided in the original source  C= coordinates estimated by the compiler as the approximate centre point of the smallest clearly defined geographical unit provided in the original source (e.g., county, state, island), or estimated based on site description in the text (e.g., location relative to reported settlements or other geographic features). Potentially lower precision. | A, B, C |
| Site | A site name or description. | Alphanumeric and special characters, variable number of characters | A description or identifier of the sample site, based on information in the original source. “Site_number” can be used to create unique site-specific time-series ID codes, but “Site” enables users to identify specific datasets, or associated data with information in the original sources. |  |
| Country | The country where the observation was recorded. | Alphanumeric and special characters, variable number of characters | The country where the observation was made.  Country names are standardised to the English short name (ISO3166-1), using the “countrycode” package for R (v. 1.2.0) (Arel-Bundock, Enevoldsen, and Yetman 2018) |  |
| Elevation | The elevation of the sample site. | Numeric, variable number of characters | The elevation of the sample site in metres above sea level, where provided in the original source. Where the elevation was not provided, a missing value is indicated by “NA”. | Numeric, NA |
| Spatial_unit | Categorical classification of spatial scale represented by the record. | Alphanumeric and special characters, variable number of characters | Categorical classification of spatial scale represented by the record, estimated by the compiler based on information provided in the original source.  stand = <100 ha  patch = 100-10,000 ha  region = 10,000-1,000,000 ha  super-region = >1,000,000 ha | stand, patch, region, super-region |
| No_individuals | The number of individuals contributing to the population-level estimate of reproductive effort. | Numeric, variable number of characters | Either the number of monitored individual plants, or the number of litter traps.  9999 indicates that while the number of monitored individuals was not specified, the compiler concluded (based on information in the source) that the sample size was likely >=10 individuals or litter traps.  NA indicates no information on the sample size in the original source. | Numeric, NA |
| Start | The final year of observations for a time-series. | Numeric, 4 characters | The start year for a time-series, incorporating all segments |  |
| End | The final year of observations for a time-series. | Numeric, 4 characters | The end year for a time-series, incorporating all segments |  |
| Length | The number of years of observations for a time-series. | Numeric, variable number of characters | Length corresponds to the number of year of observations in any time-series.  Note that may not be equal to the number of years between the Start and End of the time-series, due to gaps in the time-series. |  |
| Reference | Identification for the original source of the data. | Alphanumeric and special characters, variable number of characters | The full list of references is included in Appendix 4.  The lead author retains PDF copies of original sources. |  |
| Record_type | Categorisation of type of original source. | Alphanumeric and special characters, variable number of characters | Category providing information on the type of original data source.  Peer-reviewed = data extracted from peer reviewed literature  Grey = data extracted from grey literature  Unpublished = previously unpublished data | peer-reviewed, grey, unpublished |
| ID_enterer | Identification of the original compiler of the data. | Alphanumeric, variable number of characters | Identification of the original compiler of the record. The compiler was responsible for extracting and entering the data and meta data into MASTREE+  AHP = Andrew Hacket-Pain; ES = Eliane Schermer; JVM = Jose Moris; XTT = Tingting Xue; TC = Thomas Caignard; DV = Davide Vecchio; DA = Davide Ascoli; IP = Ian Pearse; JL = Jalene LaMontagne; JVD = Joep van Dormolen | AHP, JVM, XTT, TC, DV, DA, IP, JL, JVD |
| Date_entry | Date of data entry into MASTREE+. | Numeric and special character, format yyyy-mm-dd | Date of data entry into MASTREE+ in the format yyyy-mm-dd. |  |
| Note on data location | Notes on the location of the data within the original source. | Alphanumeric and special characters, variable number of characters | Notes made by the compiler on the location of the data within the original source. This might include the page number, figure number or table number. Not always completed. Blank entries filled with “NA” | Alphanumeric, NA |
| Comments | Additional comments. | Alphanumeric and special characters, variable number of characters | Contextual notes made by the compiler during data entry, additional information provided by the data contributor, or notes made by by AHP or JJF during data validation. | Alphanumeric, NA |
| Study_ID | Unique code associated with each source of data. | Alphanumeric and special characters, 6 characters. | Study_ID can be used to identify the original source of the data. M = series are extracted from published literature; A = series are incorporated from Ascoli et al. (2017, 2020); P = series are incorporated from Pearse et al (2017); D = previously unpublished datasets. |  |
