# Supplementary material for: MASTREE+: Time‐series of plant reproductive effort from six continents
Source: Glob Chang Biol. 2022 Mar 5;28(9):3066–82. doi: 10.1111/gcb.16130 (PMC9314730; doi:10.1111/gcb.16130)
Supplement: Supplementary file 6 — Appendix S6 [file GCB-28-3066-s002.docx]

**Appendix 6: Reproducible examples using MASTREE+**

## Loading the dataset

To read in MASTREE+, either load the .RData file, or read the .csv file.

# Load readr package to facilitate data loading and dplyr for the pipe
library(readr)

## Warning: package 'readr' was built under R version 4.0.5

library(dplyr)

## Warning: package 'dplyr' was built under R version 4.0.5

# Load .RData file
load(file = "C:/Users/jjfoest/Downloads/MASTREEplus_2022-01-13_V1.RData") # Replace this file path

# Or read .csv file (using the readr package)
mastree <- read_csv(file = "C:/Users/jjfoest/Downloads/MASTREEplus_2022-01-13_V1.csv", # Replace this file path
 col_types = cols(Year = col_integer())) # specify that Year should be of the integer type, not a double

## Filtering

We can subset the dataset in R, to focus on records or time-series of interest. Records can be filtered with functions such as dplyr::filter(). If you want to filter all time-series which *contain* a specific value, (e.g., they have a record in the year 2013) the mastree object should be grouped first with its key (i.e. indicators of a unique series) before filtering, adding any() around the to be contained value.

# filtering records ----

# we can save our selection to a new object (named abicepfor)
abicepfor <- mastree %>%
 filter(
 # For instance, only look at records with Continuous data
 VarType == "C" &
 # from Abies cephalonica or Abies forrestii
 Species_code %in% c("ABICEP", "ABIFOR")
 )

# show abicepfor
abicepfor

## # A tibble: 34 x 32
## Alpha_Number Segment Site_number Variable_number Year Species Species_code
## <chr> <chr> <chr> <chr> <int> <chr> <chr>
## 1 2280 01 001 01 2008 Abies fo~ ABIFOR
## 2 2280 01 001 01 2009 Abies fo~ ABIFOR
## 3 2280 01 001 01 2010 Abies fo~ ABIFOR
## 4 2280 01 002 01 2008 Abies fo~ ABIFOR
## 5 2280 01 002 01 2009 Abies fo~ ABIFOR
## 6 2280 01 002 01 2010 Abies fo~ ABIFOR
## 7 2280 01 003 01 2008 Abies fo~ ABIFOR
## 8 2280 01 003 01 2009 Abies fo~ ABIFOR
## 9 2280 01 003 01 2010 Abies fo~ ABIFOR
## 10 2280 01 004 01 2008 Abies fo~ ABIFOR
## # ... with 24 more rows, and 25 more variables: Mono_Poly <chr>, Value <dbl>,
## # VarType <chr>, Max_value <dbl>, Unit <chr>, Variable <chr>,
## # Collection_method <chr>, Latitude <dbl>, Longitude <dbl>,
## # Coordinate_flag <chr>, Site <chr>, Country <chr>, Elevation <dbl>,
## # Spatial_unit <chr>, No_indivs <dbl>, Start <dbl>, End <dbl>, Length <dbl>,
## # Reference <chr>, Record_type <chr>, ID_enterer <chr>, Date_entry <date>,
## # Note on data location <chr>, Comments <chr>, Study_ID <chr>

# filtering time-series that contain a specific value ----

# to minimise typing, save the key as a list
key <- c("Alpha_Number", "Site_number", "Variable_number", "Species_code")

# Saving our selection to a new object (named abicepfor_2013)
abicepfor_2013 <- mastree %>%
 group_by_at(key) %>% # group by the key
 filter(
 # Let's look at continuous series from Abies cephalonica or Abies forrestii
 VarType == "C" &
 Species_code %in% c("ABICEP", "ABIFOR") &
 # that have a record in 2013
 any(Year == 2013)
 )

# show abicepfor_2013
abicepfor_2013

## # A tibble: 10 x 32
## # Groups: Alpha_Number, Site_number, Variable_number, Species_code [2]
## Alpha_Number Segment Site_number Variable_number Year Species Species_code
## <chr> <chr> <chr> <chr> <int> <chr> <chr>
## 1 2503 01 001 01 2013 Abies ce~ ABICEP
## 2 2503 01 001 01 2014 Abies ce~ ABICEP
## 3 2503 01 001 01 2015 Abies ce~ ABICEP
## 4 2503 01 001 01 2016 Abies ce~ ABICEP
## 5 2503 01 001 01 2017 Abies ce~ ABICEP
## 6 2503 01 001 02 2013 Abies ce~ ABICEP
## 7 2503 01 001 02 2014 Abies ce~ ABICEP
## 8 2503 01 001 02 2015 Abies ce~ ABICEP
## 9 2503 01 001 02 2016 Abies ce~ ABICEP
## 10 2503 01 001 02 2017 Abies ce~ ABICEP
## # ... with 25 more variables: Mono_Poly <chr>, Value <dbl>, VarType <chr>,
## # Max_value <dbl>, Unit <chr>, Variable <chr>, Collection_method <chr>,
## # Latitude <dbl>, Longitude <dbl>, Coordinate_flag <chr>, Site <chr>,
## # Country <chr>, Elevation <dbl>, Spatial_unit <chr>, No_indivs <dbl>,
## # Start <dbl>, End <dbl>, Length <dbl>, Reference <chr>, Record_type <chr>,
## # ID_enterer <chr>, Date_entry <date>, Note on data location <chr>,
## # Comments <chr>, Study_ID <chr>

## Basic plot

Now we have some subsets to work with, we can make a plot.

# load the ggplot2 package to facilitate plotting
library(ggplot2)

## Warning: package 'ggplot2' was built under R version 4.0.5

# plot
abiplot <- abicepfor %>%
 # preparation
 group_by_at(key) %>% # group by the key
 mutate(
 time_series = cur_group_id(), # make a variable that captures unique key combinations
 Year = parse_date(as.character(Year), format = "%Y") # consider saving the Year column to a date for clean axis labels
 ) %>%
 # plotting
 ggplot(
 aes(
 # draw a canvas with x and y axes, specifying that each time-series as a group that belongs together
 x = Year, y = Value, group = time_series,
 # we can add different legend mappings here too
 colour = Species, linetype = Unit
 )
 ) +
 geom_line() + # draw the lines
 theme_bw() # show the plot on a black white graph (default is grey)

# show
abiplot


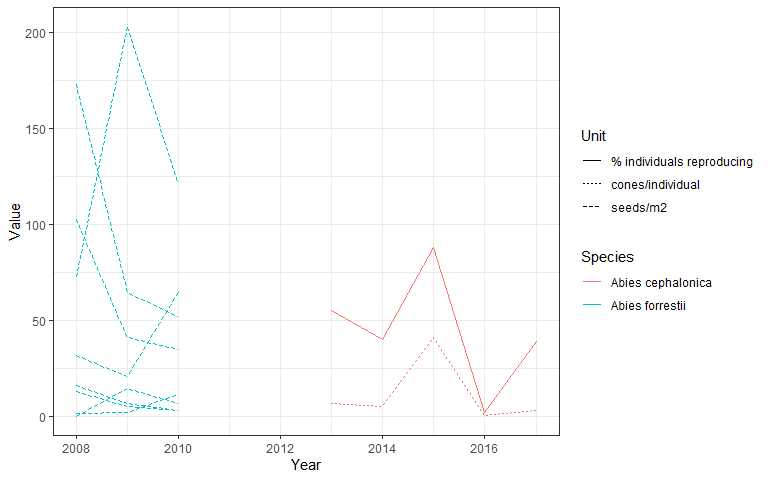


# plot, after drawing a separate graph space for each species
abiplot +
 facet_wrap(~Species, scales = "free")+ # break the plot up by Species name
 scale_x_date(date_labels = "%Y") # specify how you want the date to plot. In this case, we want to show the year (%Y) only.


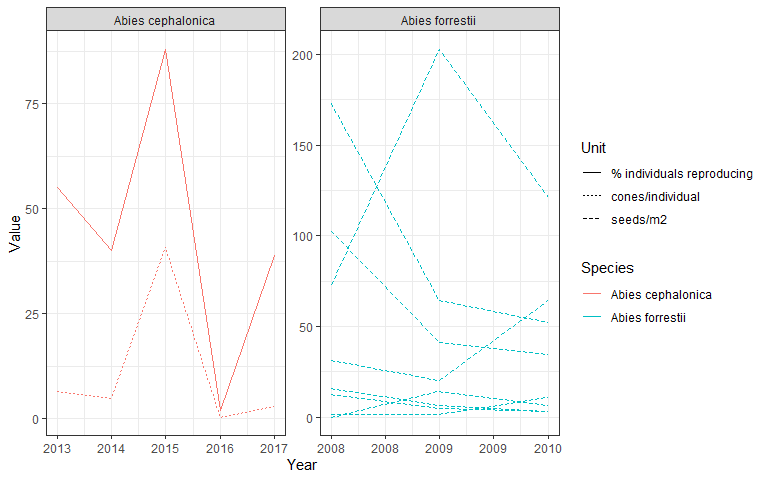


## Reformatting

It is also possible to quickly change the data layout, using tidyr’s pivot_*() commands.

For instance:

abicepfor %>%
 # only select some columns, purely for aesthetic/illustration purposes
 select(all_of(key), Year, Value) %>%
 # reformat
 tidyr::pivot_wider(
 names_from = Year,
 values_from = Value
 )

## # A tibble: 10 x 12
## Alpha_Number Site_number Variable_number Species_code `2008` `2009` `2010`
## <chr> <chr> <chr> <chr> <dbl> <dbl> <dbl>
## 1 2280 001 01 ABIFOR 173. 64.5 51.9
## 2 2280 002 01 ABIFOR 102. 40.9 34.6
## 3 2280 003 01 ABIFOR 12.6 4.7 3.1
## 4 2280 004 01 ABIFOR 15.7 6.3 3.1
## 5 2280 005 01 ABIFOR 72.3 203. 121.
## 6 2280 006 01 ABIFOR 31.4 20.4 64.5
## 7 2280 007 01 ABIFOR 1.3 1.6 11
## 8 2280 008 01 ABIFOR 0 14.2 6.3
## 9 2503 001 01 ABICEP NA NA NA
## 10 2503 001 02 ABICEP NA NA NA
## # ... with 5 more variables: 2013 <dbl>, 2014 <dbl>, 2015 <dbl>, 2016 <dbl>,
## # 2017 <dbl>
